# *Valeriana pilosa* Roots Essential Oil: Chemical Composition, Antioxidant Activities, and Molecular Docking Studies on Enzymes Involved in Redox Biological Processes

**DOI:** 10.3390/antiox11071337

**Published:** 2022-07-07

**Authors:** Patricia Minchán-Herrera, Roberto O. Ybañez-Julca, Ivan M. Quispe-Díaz, Edmundo A. Venegas-Casanova, Rafael Jara-Aguilar, Felipe Salas, Liz Zevallos-Escobar, Osvaldo Yáñez, Ricardo Pino-Rios, Pedro Buc Calderon, Julio Benites

**Affiliations:** 1 Facultad de Farmacia y Bioquímica, Universidad Nacional de Trujillo, Trujillo 13011, Peru; pminchan@unitru.edu.pe (P.M.-H.); iquispe@unitru.edu.pe (I.M.Q.-D.); evenegas@unitru.edu.pe (E.A.V.-C.); djara@unitru.edu.pe (R.J.-A.); 2 Química y Farmacia, Facultad de Ciencias de la Salud, Universidad Arturo Prat, Casilla 121, Iquique 1100000, Chile; felisala@unap.cl (F.S.); pedro.buccalderon@uclouvain.be (P.B.C.); 3 Escuela de Farmacia y Bioquímica, Universidad Católica Los Ángeles de Chimbote, Chimbote 02801, Peru; lzevallose@uladech.edu.pe; 4 Facultad de Ingeniería y Negocios, Universidad de las Américas, Santiago 7500000, Chile; oyanez@udla.cl; 5 Instituto de Ciencias Químicas Aplicadas, Facultad de Ingeniería, Universidad Autónoma de Chile, Santiago 7500912, Chile; ricardopinor@gmail.com; 6 Research Group in Metabolism and Nutrition, Louvain Drug Research Institute, Université Catholique de Louvain, 73 Avenue E. Mounier, GTOX 7309, 1200 Brussels, Belgium

**Keywords:** *Valeriana pilosa*, antioxidant activities, molecular docking, antioxidant enzyme, oxidative stress

## Abstract

*Valeriana pilosa* is usually employed in Peruvian folk medicine in the form of infusion to treat stomach pain, and has antispasmodic, relaxing, sleep-promoting, and sedative properties, as well as is an anti-inflammatory. In this study, *Valeriana pilosa* essential oil (VPEO) was obtained by hydrodistillation, analyzed by GC and GC/MS, and 47 compounds were identified. Major oil components were α-patchoulene (5.8%), α-humulene (6.1%), seychellene (7.6%), and patchoulol (20.8%). Furthermore, we assessed the in vitro antioxidant activities, molecular docking, and Ligand Efficiency studies on enzymes involved in cellular redox pathways such as CYP2C9, catalase, superoxide dismutase, and xanthine oxidase. Essential oil antioxidant activities were assessed by FRAP, ABTS^•+^, and DPPH^•^ radical scavenging activity. VPEO displays high antioxidant activity as compared to essential oils of *Valeriana jatamansi* and *Valeriana officinalis* oil roots. In addition, molecular docking and ADMET prediction was employed to compare the absorption, metabolism, and toxicity properties of *Valeriana pilosa* compounds. In the molecular docking studies, limonene, *p*-cimene, carvone, α-cubebene, cyclosativene, α-guaiene, allo-aromadendrene, valencene, and eremophyllene were the compounds with the best docking score on CYP2C9 and xanthine oxidase. Thus, volatile components of *Valeriana pilosa* could be associated with the detected antioxidant activity, acting as putative inhibitors of CYP2C9 and xanthine oxidase.

## 1. Introduction

Sociocultural and health care necessities of rural people of emerging and developing countries are mainly assured by the use of curative and aromatic plants. Despite advances in modern medicine, it is estimated that over 80% of the developing world’s population still relies on traditional medicines (mainly herbs) to provide their health care needs, a tendency essentially imputed to strong cultural beliefs, accessibility, and low costs [1].

Natural compounds such as essential oils are complex mixtures, including typically volatile compounds, which have been employed in traditional and modern medicines as well as in perfumes and cosmetics manufacturing, aside from pharmaceutical therapies and herbal beverages [2,3].

The sub-family Caprifoliaceae of the genus *Valeriana* contains more than 350 species distributed throughout the temperate Northern Hemisphere, Africa, and South America. In the Andean region, it represents an important center of secondary diversification. Numerous species are currently employed worldwide as medicines [4,5]. In Peru, about 73 species have been reported, and among them 45 species are endemic [6]. *Valeriana pilosa* has been widely used by local inhabitants for stomach distress and epilepsy, for its antispasmodic, relaxing, sleep-promoting, and sedative properties, and even as an anti-inflammatory [7,8,9]. Peruvian folk medicine often refers to *Valeriana pilosa* as “Valeriana”, “Coche coche’’, ‘‘Valeriana de paramo’’, ‘‘Ornamo’’, or “Babilla”.

The volatile components of plant species from Chilean and Peruvian Andean highlands communities have been one of the focuses of our chemical and biological research projects [10,11,12,13,14,15]. Since the chemical composition of VPEO has not yet been investigated, the aim of this study was to identify and quantify the components of VPEO from roots of *Valeriana pilosa*. In addition to the chemical composition, we investigated its antioxidant activities, and performed molecular docking studies on some redox enzymes and ADMET profiles.

Briefly, the present investigation aims to (i) determine the chemical composition of VPEO; (ii) evaluate the in vitro antioxidant activities; (iii) carry out in silico studies about the inhibitory effect of VPEO volatile phytochemicals on the crystal structure of some critical proteins; and (iv) perform ADMET prediction of VPEO compounds.

The results obtained in this study may supply further guidance for the correct use of *Valerian pilosa*. Moreover, we advocate for better protection of this herb within the context of a growing demand in the market.

## 2. Materials and Methods

### 2.1. Plant Material

*Valeriana pilosa* R & P plants were collected in January 2021 in the Community of San Juan de Corralpampa at 3500 m above sea level, in the Hualgayoc Province, Department of Cajamarca, Peru. The specimen was identified and deposited in the “Herbarium Truxillense de la Facultad de Ciencias Biologicas de la Universidad Nacional de Trujillo” (voucher specimen HUT 61241–61242).

### 2.2. Essential Oil Isolation

Essential oil of the roots (50 g) was extracted through hydrodistillation for 3 h using a Clevenger-type apparatus. The yield was determined based on a moisture-free basis as 0.20% (*w*/*w*). The oil obtained was dried over anhydrous Na_2_SO_4_. VPEO was filtered, and the sample container was tightly sealed and stored at +4 °C until analysis.

### 2.3. Gas Chromatography Analysis (GC)

All chemicals were of analytical reagent grade, and they were obtained from Sigma-Aldrich-Fluka (St. Louis, MO, USA), Merck (Darmstadt, Germany) and were employed as supplied. The VPEO was studied on a Perkin Elmer Clarus 600 gas chromatograph according to procedures reported by Benites et al. [15].

### 2.4. Gas Chromatography-Mass Spectrometry (GC-MS)

The GC-MS analysis of the VPEO was carried out as reported previously [15]. Briefly, analyses were performed on a Perkin Elmer Clarus 600 gas chromatograph, consisting of a DB-1 fused-silica column (30 m × 0.25 mm i.d., film thickness 0.25 μM; J & W Scientific, Folsom, CA, USA), and interfaced with a Perkin Elmer Clarus 600T mass spectrometer (software version 4.1, Perkin Elmer, Shelton, CT, USA). Both injector and oven temperatures were as above; transfer line temperature, 280 °C; ion source temperature, 220 °C; carrier gas, helium, adapted to a linear rate of 30 cm/s; split ratio, 1:40; ionization energy, 70 eV; scan range, 40–300 *m*/*z*; scan time, 1 s. The identification of components was achieved by comparing their retention indices, relative to C_9_–C_21_ *n*-alkane indices and GC-MS spectra from a homemade library, made by analyses of reference oils, laboratory-synthesized components, and commercial sample standards.

### 2.5. Antioxidant Capacity Assays

#### 2.5.1. Ferric-Reducing Antioxidant Power (FRAP) Assay

The FRAP assay was performed as previously reported [16] with the following adjustments. Briefly, the FRAP stock solutions included 300 mM acetate buffer pH 3.6, 10 mM TPTZ (2,4,6-tripyridyl-*_S_*-triazine) solution in 40 mM HCl, and 20 mM FeCl_3_ × 6H_2_O. The working solution was made by mixing 25 mL of acetate buffer, 2.5 mL of TPTZ, and 2.5 mL of FeCl_3_ × 6H_2_O. Prior to use the solution was heated at 3 °C. A stock solution of 0.5 mM of the Trolox^®^ (Sigma-Aldrich-Fluka, St. Louis, MO, USA) was further prepared by serial dilutions (0.05, 0.1, 0.2, 0.3, 0.4, and 0.5 mM).

Aliquots of VPEO (8 μL) were let to react with 200 μL of the fresh FRAP solution for 30 min in the dark. Afterwards, the absorbance of colored product ferrous tripyridyltriazine complex was read at 593 nm (*n* = 3). The standard curve was made with the standard antioxidant Trolox^®^. Results are expressed as mM of TEAC (Trolox^®^ equivalents)/mL of VPEO.

#### 2.5.2. ABTS^•+^ Free Radical Scavenging Activity

The ABTS^•+^ radical assay was performed according to procedures reported by Re et al. [17]. First, Trolox^®^ solution (1 mg/mL) was prepared by dissolving it in ethanol (EtOH) and further stored in the dark. Stock solutions were successively diluted in 96-well microplates to final concentrations of 50, 100, 200, 300, 400, 600, 700, and 800 μM. The radical discoloration was initiated by adding 10 μL of each dilution into 300 μL ABTS radical cation solution, and the resulting absorbance was measured at 750 nm. For essential oil analysis, 10 μL of VPEO was used instead of 10 μL of Trolox^®^. Afterwards, a curve of % ABTS^•+^ radical versus concentration was plotted and IC_50_ values were extrapolated. IC_50_ implies the concentration of sample required to scavenge 50% of ABTS radical cation.

#### 2.5.3. DPPH Free Radical Scavenging Activity

Free radical scavenging activity was assessed by using a stable free radical, namely DPPH (2,2-diphenyl-1-picrylhydrazyl), following the modified method reported by Baran et al. [18]. Trolox^®^ solution (1 mg/mL) was made by dissolution in EtOH and further kept in the dark. Final concentrations of 1, 0.8, 0.6, 0.4, 0.2, and 0.1 mM were made in 96-well microplates by consecutive dilution of stock solutions. A mix of 300 μL DPPH radical solution and 20 μL of each dilution was incubated for 30 min at room temperature, and the absorbance was measured at 517 nm. For the essential oil analysis, 20 μL of VPEO was used instead of 20 μL of Trolox^®^. Results are expressed as IC_50_. All experiments were conducted in triplicate, and data are expressed as mean values ± SD.

### 2.6. In Silico Studies

#### 2.6.1. Molecular Docking and Ligand Efficiency

To explore the ability of VPEO to act as potential protein inhibitor, compounds **1** to **47** were subjected to a molecular docking analysis looking for their binding modes on the following proteins: CYP2C9 [19], catalase [20], superoxide dismutase [21], and xanthine oxidase [22]. For all docking studies conducted in this study, AutoDock (v 4.2.1, Scripps Research Institute, San Diego, CA, USA) and AutoDock Vina (v 1.0.2, Scripps Research Institute, San Diego, CA, USA) were employed [23]. The three-dimensional coordinates of all structures were optimized using MOPAC2016 [24] software by PM6-D3H4 semi-empirical method [25,26] (see Tables S1, S2, and S6 for smiles and mol2 files in the Supplementary Material). AutoDockTools package was used to prepare the ligand files [27]. The crystal structure of CYP2C9 (PDB Code: 1OG5), catalase (PDB Code: 1TGU), superoxide dismutase (PDB Code: 2SOD), and xanthine oxidase (PDB Code: 3NRZ) were downloaded from the Protein Data Bank [28]. These four proteins were treated with the Schrödinger’s Protein Preparation Wizard [29]; polar hydrogen atoms were included, non-polar hydrogen atoms were merged, and charges were assigned. Docking was treated as rigid and performed using the empirical free energy function and the Lamarckian Genetic Algorithm provided by AutoDock Vina [30]. The grid map dimensions were 20 × 20 × 20 Å^3^. The creation of the binding pocket of superoxide dismutase was based on the center coordinates −13.910, 34.868, and 14.639, while the binding pocket of xanthine oxidase was based on the coordinates 19.480, 19.305, and 18.151. These binding sites were established in previous literature [31,32,33,34]. All other parameters were set as default defined by AutoDock Vina. Dockings were repeated 20 times with space search exhaustiveness set to 50. The best binding energy (kcal·mol^−1^) was selected for evaluation. For docking result 3D representations, the Discovery Studio [35] 3.1 (Accelrys, San Diego, CA, USA) molecular graphics system was used.

Ligand Efficiency (*LE*) was calculated by using *K_d_*, a dissociation constant indicating the bond strength between the ligand/protein [36,37,38]. *K_d_* was calculated by applying the following equations:

(1) $$\Delta G^{0}=-2.303\mathrm{RTlog}\left(K_{d}\right)$$

(2) $$K_{d}=10^{\frac{\Delta G^{0}}{2.303\mathrm{RT}}}$$

where ∆G^0^ is the binding energy (BE, in kcal·mol^−1^) found from docking experiments, R is the gas constant, and T is the temperature in Kelvin, in standard conditions of aqueous solution at 298.15 K, neutral pH, and remaining concentrations of 1 M. As indicated in Equation (3), *LE* allows the comparison of molecules according to their average binding energy [38,39]. Thus, it is determined as the ratio of binding energy per non-hydrogen atom, as follows [36,37,38,40]:

(3) $$\;LE=-\frac{2.303\mathrm{RT}}{\mathrm{HAC}}\log\left(K_{d}\right)$$

where *K_d_* is obtained from Equation (2) and HAC denotes the heavy atom count (i.e., number of non-hydrogen atoms) in a ligand.

On the other hand, Binding Efficiency Index (*BEI*) and Lipophilic Ligand Efficiency (*LLE*) are calculated using the *K_d_* obtained from molecular docking. *BEI* allows to calculate the binding capacity weighted by molar mass (Equation (4)), whereas *LLE* (Equation (5)) determines the binding capacity with respect to its lipophilicity (clogP obtained from SwissADME webserver) [41,42].

(4) $$BEI=\frac{-\log\left(K_{d}\right)}{MW}\;$$

(5) $$LLE=-\log\left(K_{d}\right)-\mathrm{clogP}$$

To complement this Ligand Efficiency study, an additional analysis of the size of the molecules in relation to the binding energy was implemented, the score normalization based on the number of non-hydrogen atoms. This score-based approach ($IE_{norm,\;\mathrm{binding}}$) is biased toward the selection of high molecular weight compounds because of the contribution of the compound size to the energy score [43]. Such biasing behavior was observed to depend on the shape and chemical properties of the binding pocket. The procedure starts with the normalization of the binding energy ($IE_{\mathrm{binding}}$) by the number of heavy atoms (HAC) or by a selected power of HAC in each respective compound. This normalization approach shifts the MW distribution of selected compounds into better agreement with that of the VPEO database. In the present study, the following equation was used to calculate the normalized binding energy value.

(6) $$IE_{norm,\;\mathrm{binding}\;}=\frac{IE_{\mathrm{binding}\;}}{{\mathrm{HAC}}^{\frac{1}{2}}}$$

#### 2.6.2. Non-Covalent Interactions

To qualitatively identify regions where intermolecular interactions such as steric repulsion, hydrogen bonds, and Van der Waals interactions predominate in the structural protein–ligand, the non-covalent interaction index (NCI) was employed [44,45]. For biological systems studies, the NCI is based on the promolecular electron density, its derivatives, and the reduced density gradient, as reported elsewhere [46,47]. Molecular visualization of the systems was conducted by using the VMD software package [48].

### 2.7. ADMET Prediction

The pkCSM online tool (http://biosig.unimelb.edu.au/pkcsm/prediction, accessed on 7 February 2022) [49], was utilized to predict absorption, distribution, metabolism, excretion, and toxicity (ADMET) of VPEO.

### 2.8. Statistical Analysis

GraphPad Prism 8.0.2 software (San Diego, CA, USA) was used for statistical analysis. The IC_50_ value was established by a nonlinear regression analysis.

## 3. Results and Discussion

### 3.1. Chemical Composition of VPEO Roots

Data from gas chromatography (GC) and GC-MS analysis of plant root VPEO are shown in Table 1. The VPEO components were identified by comparing the GC retention indices (RI) on polar and non-polar columns. Such constituents were determined according to the retention time of a series of *n*-alkanes with linear interpolation with those standards and our essential oils database. Forty-seven compounds were revealed by the GC analysis of the essential oil, accounting for 87.5% of the total composition. The major constituents were sesquiterpene hydrocarbons (37.7%), while the monoterpene hydrocarbons were present in concentrations of 9.5%. The oxygen-containing sesquiterpenes were prevalent (26.6%) as compared to oxygen-containing monoterpenes (8.3%). In addition, other compounds were present in low concentrations in oil (5.7%). Notable differences in valerian root oil composition have been reported, a fact likely due to a different geographical environment, crop type, season, plant physiological age, and the method of oil isolation [50,51].

**Figure 1:**
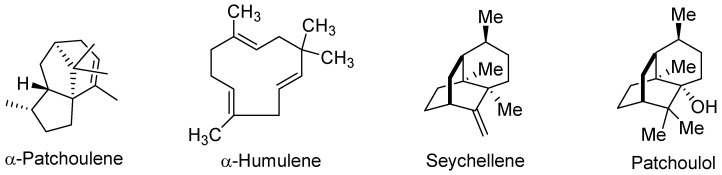
Chemical structures of abundant compounds identified in the essential oil of *Valeriana pilosa* roots.

Figure 1 shows the major constituents of the VPEO, which included natural sesquiterpenes such as α-patchoulene (5.8%), α-humulene (6.1%), seychellene (7.6%), and patchoulol (20.8%). However, different VPEO constituents have been shown by chemical analysis; for instance, the essential oil of *Valeriana jatamansi* roots from India contained only seven major sesquiterpene components, which were identified as β-vatirenene (28.07%), β-patchoulene (20.18%), dehydroaromadendrene (15.92%), β-gurjunene (13.0%), patchouli alcohol (11.72%), β-guaiene (5.88%), and α-muurolene (5.20%) [52]. In Vietnam, root essential oils of *Valeriana hardwickii* reported sixty-two components representing 81.6% of total oil, and the major compounds in the root oil were camphene (12.9%), bornyl acetate (17.6%), and maaliol (10.6%) [53].

Several molecules have been identified as basic oil constituents from about 15 studied *Valerian officinalis* root samples from different European countries (Belgium, Czech, Estonia, France, Germany, Greece, Hungary, Latvia, Lithuania, Moldova, Russia, Scotland, Ukraine). They contain 86 identified compounds (>90% of the total oil) such as bornyl acetate (2.9–33.7%), α-fenchene (0–28.3%), valerianol (0.2–18.2%), valerenal (tr-15.6%), isovaleric acid (0–13.1%), camphene (0–11.1%), valeranone (0.5–10.9%), valerenic acid (0–9.8%), sesquiterpene alcohol C (tr-8.0%), spathulenol (0.3–7.3%), and allo-aromadendrene (0.3–6.9%) [54]. Kessyl acetate, kessanyl acetate, and patchouli alcohol are the main constituents in many Valeriana species [55], but only the latter molecule is present in the Valeriana species under study in this work.

### 3.2. Antioxidant Capacity of VPEO

Table 2 includes VPEO antioxidant activities, which were determined by using various chemical-based methodologies. These assays have been developed on different approaches providing evidence about free radicals and essential oil interactions. Herein, the antioxidant activity of essential oils was assessed using three different assays, namely FRAP, ABTS^•+^, and DPPH.

In the FRAP assay, when the colorless Fe^3+^-TPTZ complex interacts with a potential antioxidant, it is reduced to an intense blue Fe^2+^-TPTZ. This assay has been shown to be suitable for screening antioxidant capacities and to compare the efficacy of different compounds [56]. Results from the FRAP assay show a low reducing activity of the essential oil (TEAC = 0.0421 mM) as compared to quercetin (TEAC = 143 mM), used as an antioxidant standard molecule.

The ABTS^•+^ coloring assay is currently employed to determine the antioxidant activity of a wide variety of compounds, such as hydrogen-donating antioxidants or scavengers of aqueous phase radicals and chain-breaking antioxidants or scavengers of lipid peroxyl radicals [57]. In this radical scavenging assay, the VPEO displayed a good activity (IC_50_ of 0.30 μg/mL) when compared to IC_50_ values of standards, quercetin (IC_50_ of 0.07 μg/mL) and Trolox^®^ (IC_50_ of 0.012 μg/mL).

In the DPPH assay, the reduction of the stable radical DPPH to the yellow-colored DPPH-H is employed to measure the capability of an antioxidant molecule to act as a donor of hydrogen atoms or electrons. Table 2 shows that VPEO reduced DPPH with a IC_50_ of 0.38 μg/mL; a high value as compared with essential oils of *Valeriana jatamansi* and *Valeriana officinalis* oil roots, displaying a weak radical scavenging activity with IC_50_ values 876 μg/mL [52] and 493.40 μg/mL, respectively [58]. Such high antioxidant activity of VPEO is likely due to the presence of functionalized sesquiterpenes such as patchoulol (one of the major constituents), as well as spathulenol, T-cadinol, and γ-cadinol (minor constituents).

Since essential oils are complicated mixtures composed of huge amounts of molecules, their whole biological activity is hard to be explained. Thus, numerous reports about antioxidant activity of essential oils usually refer to concepts such as synergism, antagonism, and additivity [59]. In addition, discerning the real mechanism of antioxidant activity is not an easy task. To this end, several mechanism-based explanations have been provided: free radicals scavenging; hydrogen donation; and metallic ion chelation by antioxidants [60]. Due to the high reduction ability displayed by VPEO, it can be inferred that their components might be potent natural antioxidants.

### 3.3. Molecular Docking and Ligand Efficiency Analysis of VPEO

Molecular docking is a key tool that can show insights for understanding plausible mechanisms of action displayed by in vitro biological active molecules. In this context, molecular docking was used to find a protein target as a possible mechanism that could be correlated with (and likely explain) the observed in vitro antioxidant activity of VPEO. An in-silico-based approach was used to explore whether some VPEO constituents may inhibit some proteins involved in redox biological processes. The targeted proteins were CYP2C9 (a phase I enzyme involved in oxidation of xenobiotics), catalase (enzyme regulating hydrogen peroxide intracellular levels), superoxide dismutase (enzyme catalyzing the dismutation of superoxide anion into hydrogen peroxide and molecular oxygen), and xanthine oxidase (a key enzyme regulating the formation of uric acid and superoxide anion).

**Figure 2:**
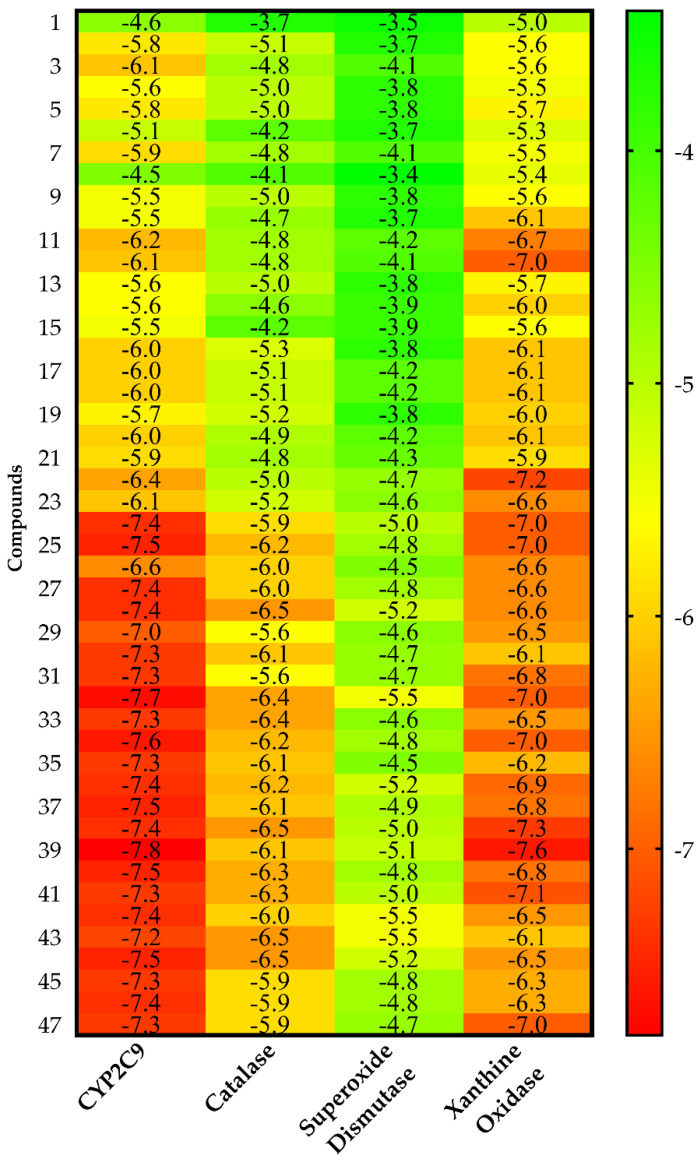
Heat map of the intermolecular docking energy values (kcal·mol^−1^) of VPEO components on CYP2C9, catalase, superoxide dismutase, and xanthine oxidase proteins. Values are listed as a three-colored scheme from red (high energy) to green (low energy).

Figure 2 shows the heat map of the intermolecular docking energy values of 47 VPEO components. The values are listed as a three-colored scheme (red-yellow-green) showing a clear trend of a set of compounds acting as putative inhibitors for a given protein. For each protein target, the range was set from red color (as the energy value corresponding to the native ligand) to green, spanning a 5 kcal·mol^−1^ interval. This approach is appropriate especially for sets of compounds sharing high structural resemblance.

Molecular dockings, *K_d_* values, Ligand Efficiency (*LE*), Binding Efficiency Index (*BEI*), and Lipophilic Ligand Efficiency (*LLE*) analyses are summarized in Table 3, as well as in Tables S2–S5. Results show that all VPEO constituents act as potential inhibitors of CYP2C9 while about 65% may be considered inhibitors of xanthine oxidase. Less than 50% of VPEO constituents appeared as rather weak inhibitors of catalase, but the vast majority of them have no effect on superoxide dismutase.

**Figure 3:**
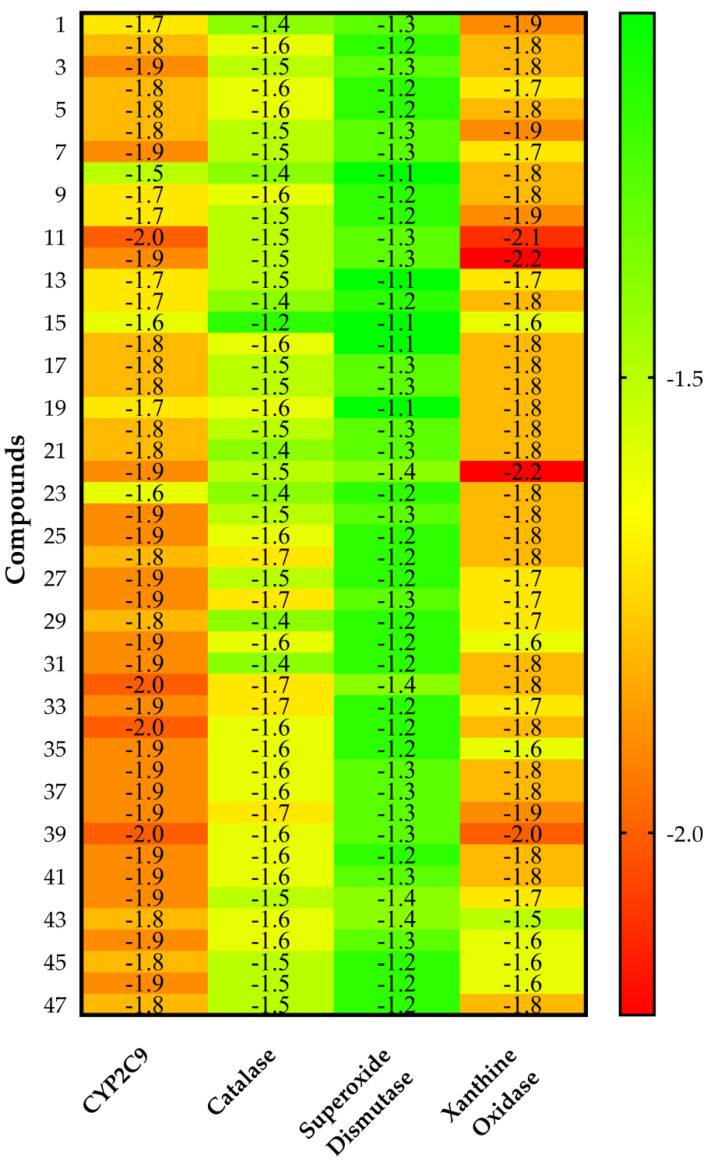
Heat map of the score normalization of the binding energy based on the number of non-hydrogen atom values (kcal·mol^−1^) of VPEO components on CYP2C9, catalase, superoxide dismutase, and xanthine oxidase proteins. Values are listed as a three-colored scheme from red (high energy) to green (low energy).

CYP2C9 appeared as the best protein target for all VPEO constituents, as shown by their intermolecular docking energy and Ligand Efficiency values. Indeed, by using the values obtained by all compounds, for CYP2C9 analysis the average values of both Binding Efficiency (*BE*) and Ligand Efficiency were −6.56 and 0.52 kcal·mol^−1^, respectively, while compared to the xanthine oxidase protein target, the average values of *BE* and Ligand Efficiency were −6.33 and 0.50 kcal·mol^−1^, respectively. Therefore, it may be assumed that CYP2C9 and xanthine oxidase are targeted proteins likely involved in VPEO effects. These results are supplemented by the calculations obtained from score normalization based on the number of non-hydrogen atoms (Figure 3). The proteins CYP2C9 and xanthine oxidase appear with a similar score of −1.8 kcal·mol^−1^, which coincide as the best protein targets for all VPEO compounds, meaning that they might be involved in the antioxidant effects of VPEO compounds.

**Figure 4:**
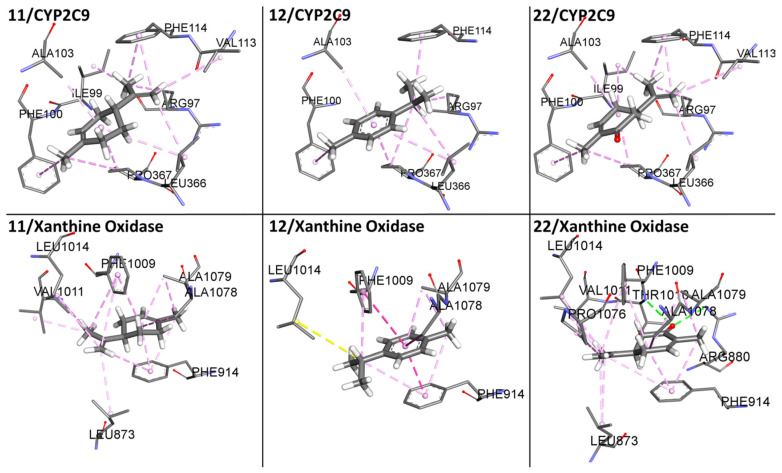
Schematic representation for the best three compounds (**11**, **12,** and **22**) of the score normalization of the binding energy based on the number of non-hydrogen atoms of VPEO bound to CYP2C9 and xanthine oxidase. The surrounding amino acid residues in the binding pocket of CYP2C9 and xanthine oxidase within 3 Å are shown.

An important aspect of normalizing binding energy is the ability to bias selection towards lower molar weight (MW) compounds, thereby identifying compounds more appropriate for lead optimization. Ligand-based postdocking structural clustering leads to the selection of diverse compounds, and many of them would have been lost through selection based on binding energy alone. Then, it is important to establish a relationship between binding energy and MW of VPEO components. Comparing the unnormalized energetic values in Figure 2 and the normalized energetic values, Figure 3 shows that there are three compounds that stand out in Figure 3, namely **11** (Limonene), **12** (*p*-Cymene), and **22** (Carvone). Figure 4 shows compounds that have low normalized energetic values of interaction with CYP2C9 (−2.0, −1.9, and −1.9 kcal·mol^−1^) and xanthine oxidase (−2.1, −2.2, and −2.2 kcal·mol^−1^). In addition, they can be considered as suitable lead molecules for a drug candidate, as they have low MW. This feature makes them advantageous because they generally exhibit better properties of being good lead candidates due to their simpler intrinsic chemical structures, rendering them suitable for further drug optimization.

In the case of non-covalent interactions between compounds **11**, **12**, and **22** with CYP2C9 and xanthine oxidase proteins (see Figure 4), weak interactions such as Van der Waals type interactions and aromatic (π—π stacking) and hydrophobic interactions are included, except for compound **22**, which forms a hydrogen bond at the binding site of xanthine oxidase.

In order to check the binding modes of the VPEO components, molecular docking simulations were performed with the co-crystallized ligand pose of warfarin bound to CYP2C9 (PDBID: 1OG5) and the co-crystallized ligand pose of quercetin bound to xanthine oxidase (PDBID: 3NVY). Such co-crystallized ligands were re-docked into the binding site with specific docking parameters and scoring functions, to check whether the docking software is reliable for the systems (Figures S1–S3). The conformation with the lowest binding energy of warfarin and quercetin was compared to the co-crystallized ligand pose. The binding energy value for warfarin was −9.8 kcal·mol^−1^ and for quercetin was −8.1 kcal·mol^−1^. The root mean square deviation (RMSD) value of the docked conformation with respect to the experimental conformation was 1.06 Å for warfarin and 1.51 Å for quercetin (Figures S1 and S2), indicating the reliability of the docking protocol, as the threshold of reliability is 2.0 Å for a good docking protocol.

Specifically, Table 3 shows the six molecules of the VPEO constituents having the best affinity for both proteins (CYP2C9 and xanthine oxidase): they include **24** (α-cubebene), **25** (cyclosativene), **32** (α-guaiene), **34** (allo-aromadendrene), **38** (valencene), and **39** (eremophyllene). Such compounds have low Δ*E_binding_* values in the range of −7.0 and −7.80 kcal·mol^−1^ but they are not abundant compounds of VPEO. In this context, we would like to stress that compounds **31** (seychellene), **33** (α-humulene), **35** (α-patchoulene), and **47** (patchoulol), which have a high abundance in VPEO, also have stable Δ*E_binding_* values: −7.3 kcal·mol^−1^ for the CYP2C9 protein and Δ*E_binding_* values ranging from −6.2 kcal·mol^−1^ to −7.0 kcal·mol^−1^ for the xanthine oxidase protein. These results show their binding tendency with regard to the CYP2C9 protein.

In addition to displaying a best affinity for both proteins, the six molecules of the VPEO constituents have strong ligand binding to the protein, as shown by their low *K_d_* values. Regarding *LE* values, they are in the range of 0.47 and 0.51 kcal·mol^−1^, compared to *LE* values greater than 0.3 kcal·mol^−1^ required to be considered as a reference [61]. According to such descriptors, compounds **24**, **25**, **32**, **34**, **38**, and **39** may be considered suitable lead molecules to a drug candidate due to their *LE*, binding energies, and their affinity for the cellular targets CYP2C9 and xanthine oxidase. Note that orally administered drugs have *LE* values between 0.50 and 0.52 kcal·mol^−1^ [62].

Regarding the Binding Efficiency Index (*BEI*), the reference values should be in the range of 20 and 27 kDa. Since *BEI* values of compounds **24**, **25**, **31**, **32**, **33**, **34**, **35**, **38**, **39**, and **47** are within such reference range, it appears that the ligands reveal a high structure–activity relationship with CYP2C9 and xanthine oxidase (see Tables S2 and S5). 

Another essential parameter to be considered is the Lipophilic Ligand Efficiency (*LLE*) index, which determines ligand-binding capacity to the protein and its lipophilic power [62]. Based on the properties of a standard oral drug, with a calculated LogP (cLogP) of ~2.5–3.0, ideal *LLE* values for an optimized drug candidate are in the range of 5 < *LLE* < 7, and were calculated based on oral administration of known drugs [63]. The *LLE* values for selected compounds, namely **24**, **25**, **31**, **32**, **33**, **34**, **35**, **38**, **39,** and **47**, are out of such range, having values lower than 5. Since cLogP had relatively high values, the ligands therefore display lipophilic properties.

**Figure 5:**
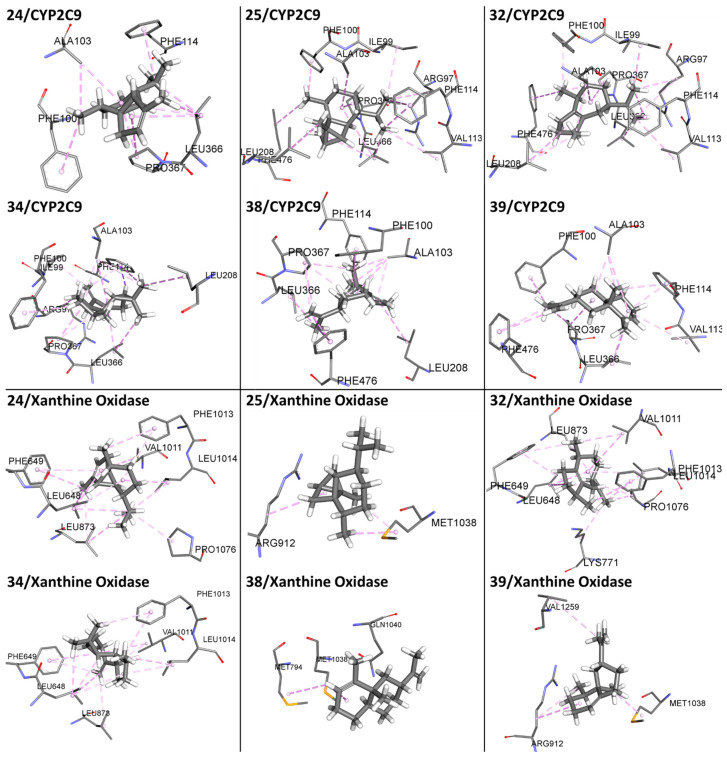
Molecular docking visualization for the best six compounds (**24**, **25**, **32**, **34**, **38**, and **39**) of VPEO bound to CYP2C9 and xanthine oxidase. The surrounding amino acid residues in the binding pocket of CYP2C9 and xanthine oxidase within 3 Å are shown.

Figure 5 shows interactions of compounds **24**, **25**, **32**, **34**, **38**, and **39** with the surrounding amino acid residues in the binding pocket of CYP2C9 and xanthine oxidase within 3 Å. They included non-covalent interactions, which are associated with weak Van der Waals type interactions and aromatic (π—π stacking) and hydrophobic interactions. It should be stressed that the main non-covalent interactions of compounds **24**, **25**, **32**, **34**, **38**, and **39** with CYP2C9 and xanthine oxidase binding sites are based on weak Van der Waals and hydrogen bond interactions. In the case of non-covalent interactions between compounds **31**, **33**, **35** and **47** with CYP2C9 and xanthine oxidase proteins (see Figure S3), weak interactions such as Van der Waals and hydrophobic interactions are included. Although compound **47** has a hydroxyl group, it does not form a hydrogen bond to stabilize this interaction, with weak Van der Waals type interactions prevailing.

Regarding aromatic and hydrophobic interactions, the most notable interaction of VPEO selected constituents with CYP2C9 residues included the following amino acids: Arg97, Ile99, Phe100, Leu102, Ala103, Val113, Phe114, Leu208, Ile213, Leu366, Pro367, and Phe476. In particular, residues Leu208, Leu366, and Phe476 form a hydrophobic patch in the active site [19].

In the case of xanthine oxidase, such interactions between VPEO selected constituents and enzyme residues were established with the following amino acids: Leu648, Phe649, Lys771, Met794, Leu873, Arg912, Val1011, Phe1013, Leu1014, Met1038, Gln1040, Pro1076, Ala1078, and Val1259.

**Figure 6:**
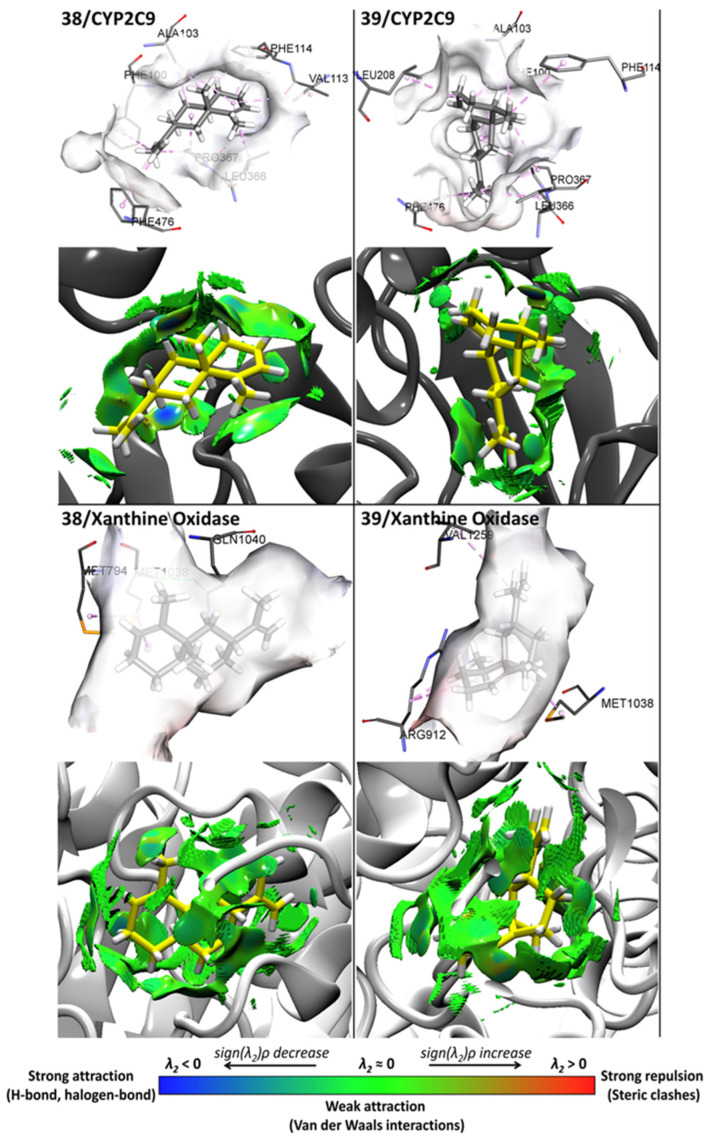
Docking and NCI analysis for the best two compounds, **38** and **39**, of VPEO essential oils bound to CYP2C9 and xanthine oxidase.

In addition to the identification of amino acid residues involved in non-covalent interactions between protein targets and selected VPEO constituents, Figure 6 shows the strengths of main non-covalent interactions for compounds **38** and **39** on the CYP2C9 and xanthine oxidase binding sites according to the NCI analysis. These interactions are mainly based on strong attraction, weak attraction, and strong repulsion (blue, green, and red colors, see bottom scale of Figure 6).

The activity of compound **38** at the CYP2C9 binding site involves Van der Waals type interactions with residues Phe100, Ala103, Phe114, Leu208, Leu366, Pro367, and Phe476. Compound **39** shows Van der Waals type interactions with residues Phe100, Ala103, Val113, Phe114, Leu366, Pro367, and Phe476. For compounds interacting at the xanthine oxidase binding site, compounds **38** and **39** display Van der Waals type interactions with residues Met794, Met1038, Gln1040, Arg912, and Val1259. Altogether, it may be concluded that hydrophobic forces emerge as the main interactions playing a key role in the mechanism of action of VPEO constituents.

Regarding the interaction between CYP2C9 (as protein target) and compound **38** (as the most active VPEO constituent), it is interesting to note that such a non-covalent interaction involves six amino acids, namely Phe100, Ala103, Phe114, Leu 366, Pro367, and Phe 476. Most of such amino acids are involved in CYP2C9 activity. Indeed, Phe114 points into the active site, playing an important role in forming interactions with substrates. Moreover, residues Phe100, Leu366, and Phe476 have been reported to form a hydrophobic patch in the active site. On the other hand, Pro367 is somehow involved in CYP2C9 hem stabilization [18].

### 3.4. ADMET Profiles of VPEO

During the processes of discovery and development of drugs, pesticides, food additives, and consumer and industrial chemicals, both pharmacokinetic and toxicity properties have a significant influence. This information is especially useful during environmental and human hazard assessment. Pharmacokinetic parameters and toxicity data were obtained by using the pkCSM Online Tool, and they are reported in Table 4.

From the ADMET results, it is found that all the structures had a molecular weight ranging between 102 and 222 g/mol, a good indicator for penetrability, because the upper limit to obtain this ability is molecular weight values of less than 500 g/mol [64]. All the molecules of VPEO show Caco−2 permeability values above 1.18 and high intestinal absorption (88.8–97.7%) as well, predicting that they would be absorbed in the small intestine [65]. VPEO compounds have skin permeability values ranging from −1.043 to −3.061 cm/h, but only the compounds **1**, **6**, **25,** and **44** have values >−2.5, suggesting that most volatile phytochemicals easily penetrate the skin adequately. Note that molecules will penetrate the skin with difficulty if the logKp value is greater than −2.5 cm/hour [66].

All the compounds have acceptable volume distribution (VDss), with values above −0.15. Since compounds **1** and **6** have log BB < 0.3 they are likely unable to penetrate the blood–brain barrier (BBB). All other compounds display mean values greater than 0.3 and are therefore able to access the brain. The data available so far indicate that permeability into the central nervous system (CNS) occurs with values ranging from −2.555 to −1.218; therefore, 46.8% of the VPEO compounds would be able to permeate the central nervous system [67].

Regarding metabolism, none of the VPEO components appeared to be CYP2D6 and CYP3A4 inhibitors and they will not interfere with CYP450 biotransformation reactions.

Excretion parameters are illustrated as total clearance. They showed that only compound **2** (tricyclene) reached a negative value (−0.073 log mL/min/kg), while the rest of the compounds have positive values, indicating a rapid excretion. In addition, the adverse interactions of all VPEO constituents with the organic cation transport 2 (OCT2) showed no potential contraindication (data not shown).

Finally, the acute oral toxicity in rats (LD_50_) ranged from 1.526 to 2.065 mol/kg, corresponding to a low toxicity. The hepatotoxicity descriptor showed that all compounds are devoid of liver toxicity.

## 4. Conclusions

A double GC and GC-MS approach was used to identify the chemical components of the essential oil from *Valeriana pilosa.* The VPEO showed to contain sesquiterpene hydrocarbons, monoterpene hydrocarbons, oxygenated monoterpenes, and oxygenated sesquiterpenes. After isolation of the in vitro antioxidant activities, molecular docking studies on enzymes involved in redox balance proteins such as CYP2C9, catalase, superoxide dismutase, and xanthine oxidase, as well as ADMET properties, were investigated. A high antioxidant activity of the oil was found as compared to values obtained with the essential oils of *Valeriana jatamansi* and *Valeriana officinalis* oil roots. In the molecular docking studies, α-cubebene, cyclosativene, α-guaiene, allo-aromadendrene, valencene, and eremophyllene were the compounds with the best docking score on CYP2C9 and xanthine oxidase. Nevertheless, when the molar weight was taken into account and energy values were normalized, three compounds, namely limonene, *p*-cymene, and carvone, were highlighted. Eremophyllene may be also included due to its improved binding to these proteins, making it suitable for further drug optimization.

Additionally, according to the ADMET prediction using the pkCSM online tool, it appeared that most of compounds display suitable pharmacokinetic properties, as shown by absorption, distribution, metabolism, excretion parameters, and low toxicities.

## Figures and Tables

**Table 1:** Percentage composition of the essential oil isolated from *Valeriana pilosa* R and P roots collected in Cajamarca, Peru.

N°	Components	RI ^a^	Relative Content (%)	Identification Method	RI Data ^b^
**1**	Isovaleric acid	886	2.6	RI,MS	827–888
**2**	Tricyclene	921	t	RI,MS	914–930
**3**	α-Thujene	924	0.1	RI,MS	905–948
**4**	α-Pinene	930	3.7	RI,MS	909–956
**5**	Camphene	938	1.4	RI,MS	929–978
**6**	3-Methyl valeric acid	947	3.1	RI,MS	941–968
**7**	Sabinene	958	0.4	RI,MS	944–980
**8**	1-Octen-3-ol	961	t	RI,MS	958–986
**9**	β-Pinene	963	0.6	RI,MS	952–986
**10**	Myrcene	975	0.1	RI,MS	962–993
**11**	Limonene	1009	3.2	RI,MS	995–1044
**12**	p-Cymene	1013	t	RI,MS	992–1072
**13**	1,8-Cineole	1015	4.3	RI,MS	1007–1046
**14**	Linalool	1074	0.1	RI,MS	1078–1107
**15**	Isopentyl isovalerate	1094	t	RI,MS	1094–1105
**16**	Camphor	1102	0.2	RI,MS	1105–1150
**17**	Menthone	1120	0.8	RI,MS	1124–1142
**18**	Isomenthone	1126	0.2	RI,MS	1132–1159
**19**	Borneol	1134	t	RI,MS	1140–1188
**20**	Neomenthol	1139	t	RI,MS	1153–1176
**21**	Menthol	1148	1.2	RI,MS	1141–1185
**22**	Carvone	1210	0.1	RI,MS	1210–1246
**23**	Menthyl acetate	1278	1.4	RI,MS	1276–1294
**24**	α-Cubebene	1345	0.2	RI,MS	1340–1360
**25**	Cyclosativene	1363	0.1	RI,MS	1363–1368
**26**	α-Copaene	1375	1.0	RI,MS	1351–1407
**27**	β-Patchoulene	1378	0.4	RI,MS	1375–1380
**28**	β-Bourbonene	1379	0.4	RI,MS	1346–1396
**29**	β-Elemene	1388	0.8	RI,MS	1362–1410
**30**	β-Caryophyllene	1414	3.5	RI,MS	1411–1421
**31**	Seychellene	1431	7.6	RI,MS	1457–1461
**32**	α-Guaiene	1437	4.1	RI,MS	1409–1490
**33**	α-Humulene	1447	6.1	RI,MS	1428–1489
**34**	allo-Aromadendrene	1456	2.2	RI,MS	1442–1474
**35**	α-Patchoulene	1457	5.8	RI,MS	1457–1486
**36**	γ-Muurolene	1469	1.0	RI,MS	1449–1502
**37**	Germacrene-D	1474	0.4	RI,MS	1451–1519
**38**	Valencene	1484	0.3	RI,MS	1458–1495
**39**	Eremophyllene	1490	0.3	RI,MS	1490–1492
**40**	γ-Cadinene	1500	0.2	RI,MS	1480–1531
**41**	7-epi-α-Selinene	1503	2.5	RI,MS	1503–1540
**42**	δ-Cadinene	1505	0.8	RI,MS	1486–1563
**43**	Spathulenol	1552	1.6	RI,MS	1552–1622
**44**	β-Caryophyllene oxide	1561	2.9	RI,MS	1549–1617
**45**	T-Cadinol	1616	0.5	RI,MS	1611–1644
**46**	δ-Cadinol	1618	0.5	RI,MS	1618–1652
**47**	Patchoulol	1625	20.8	RI,MS	1625–1666

^a^ RI—retention index as determined on the DB-1 column using the homologous series of *n*-alkanes (C_9_–C_21_); t—trace (<0.05). ^b^ RI data—retention index data reported in plant essential oils on non-polar column (www.webbook.nist.gov, accessed on 21 March 2022).

**Table 2:** Antioxidant activities of essential oil of *Valeriana pilosa*.

Samples	FRAP (mM TEAC)	ABTS^•+^ IC_50_	DPPH IC_50_
VPEO	0.0421 ± 0.02	0.30 ± 0.05	0.38 ± 0.07
Quercetin	143.00 ± 0.04	0.07 ± 0.03	0.06 ± 0.02
Trolox^®^	-	0.012 ± 0.07	0.011 ± 0.04

FRAP = ferric-reducing antioxidant power; ABTS^•+^ = 2,2′-azinobis (3-ethylbenzothiazoline-6-sulfonic acid); DPPH = 2,2-diphenyl-1-picrylhydrazyl radical; GAE = gallic acid equivalent; TEAC = Trolox^®^ equivalent antioxidant capacity. Results are expressed as mean values ± SEM (*n* = 3).

**Table 3:** Molecular docking results for the best six compounds of VPEO regarding CYP2C9 and xanthine oxidase. Intermolecular docking energy values (Δ*E_binding_*), *K_d_* values, Ligand Efficiency (*LE*), Binding Efficiency Index (*BEI*), and Lipophilic Ligand Efficiency (*LLE*) for the CYP2C9 and xanthine oxidase complexes.

	Docking and Ligand Efficiency Analysis				
Compounds	Δ*E_binding_* (kcal·mol^−1^)	*K_d_*	*LE* (kcal·mol^−1^)	*BEI* (kDa)	*LLE*
	CYP2C9				
**24**	−7.40	3.77 × 10^−6^	0.49	26.54	1.15
**25**	−7.50	3.19 × 10^−6^	0.50	26.90	1.54
**32**	−7.70	2.27 × 10^−6^	0.51	27.61	0.92
**34**	−7.60	2.69 × 10^−6^	0.51	27.26	1.30
**38**	−7.40	3.77 × 10^−6^	0.49	26.54	0.70
**39**	−7.80	1.92 × 10^−6^	0.52	27.97	0.99
	Xanthine Oxidase				
**24**	−7.00	7.41 × 10^−6^	0.47	25.10	0.86
**25**	−7.00	7.41 × 10^−6^	0.47	25.10	1.17
**32**	−7.00	7.41 × 10^−6^	0.47	25.10	0.40
**34**	−7.00	7.41 × 10^−6^	0.47	25.10	0.86
**38**	−7.30	4.47 × 10^−6^	0.49	26.18	0.62
**39**	−7.60	2.69 × 10^−6^	0.51	27.26	0.84

**Table 4:** ADMET properties of chemical constituents of the *Valeriana pilosa* essential oil.

		Property									
		Absorption			Distribution			Metabolism	Excretion	Toxicity	
		Model Name									
N°	Components	Caco−2	IA	SP	VD ss	BBB	CNS	CYP2D6/CYP3A4 Inhibitor	TC	Oral Rat Acute Tox.(LD_50_)	Oral Rat Chronic Tox.-LOAEL
**1**	Isovaleric acid	1.578	88.820	−2.730	−0.937	−0.227	−2.229	No/No	0.391	1.644	2.691
**2**	Tricyclene	1.353	93.922	−1.912	0.781	0.849	−1.924	No/No	−0.073	1.608	2.103
**3**	α-Thujene	1.386	95.256	−1.371	0.575	0.810	−1.793	No/No	0.077	1.589	2.243
**4**	α-Pinene	1.38	96.041	−1.827	0.667	0.791	−2.201	No/No	0.043	1.770	2.262
**5**	Camphene	1.387	94.148	−1.435	0.547	0.787	−1.710	No/No	0.049	1.554	2.247
**6**	3-Methyl valeric acid	1.574	95.413	−2.732	−0.752	−0.198	−2.512	No/No	0.441	1.656	2.632
**7**	Sabinene	1.404	95.356	−1.342	0.566	0.836	−1.463	No/No	0.071	1.549	2.309
**8**	1-Octen-3-ol	1.481	93.214	−1.760	0.134	0.514	−2.291	No/No	0.461	1.722	1.915
**9**	β-Pinene	1.385	95.525	−1.653	0.685	0.818	−1.857	No/No	0.030	1.673	2.28
**10**	Myrcene	1.400	94.696	−1.043	0.363	0.781	−1.902	No/No	0.438	1.643	2.406
**11**	Limonene	1.401	95.898	−1.721	0.396	0.732	−2.370	No/No	0.213	1.880	2.336
**12**	p-Cymene	1.527	93.544	−1.192	0.697	0.478	−1.397	No/No	0.239	1.827	2.328
**13**	1,8-Cineole	1.485	96.505	−2.437	0.491	0.368	−2.972	No/No	1.009	2.010	2.029
**14**	Linalool	1.493	93.163	−1.737	0.152	0.598	−2.339	No/No	0.446	1.704	2.024
**15**	Isopentyl isovalerate	1.182	95.333	−1.745	−0.036	0.602	−1.818	No/No	0.481	1.582	2.271
**16**	Camphor	1.499	95.965	−2.002	0.331	0.612	−2.158	No/No	0.109	1.653	1.981
**17**	Menthone	1.520	96.739	−1.909	0.201	0.593	−2.117	No/No	0.244	1.691	2.095
**18**	Isomenthone	1.229	97.324	−1.872	0.174	0.607	−2.155	No/No	0.244	1.796	2.028
**19**	Borneol	1.484	93.439	−2.174	0.337	0.646	−2.331	No/No	1.035	1.707	1.877
**20**	Neomenthol	1.505	94.213	−2.087	0.207	0.573	−2.290	No/No	1.182	1.733	1.991
**21**	Menthol	1.376	95.257	−1.919	0.137	0.584	−2.119	No/No	1.182	1.946	2.017
**22**	Carvone	1.413	97.702	−2.145	0.179	0.588	−2.478	No/No	0.225	1.860	1.972
**23**	Menthyl acetate	1.698	96.497	−2.208	0.125	0.539	−2.390	No/No	1.207	1.823	2.040
**24**	α-Cubebene	1.389	95.964	−1.997	0.717	0.860	−1.552	No/No	0.980	1.568	1.364
**25**	Cyclosativene	1.360	95.698	−2.526	0.747	0.946	−1.422	No/No	0.771	1.689	1.366
**26**	α-Copaene	1.374	96.221	−2.225	0.806	0.887	−1.659	No/No	0.950	1.644	1.356
**27**	β-Patchoulene	1.400	95.658	−1.730	0.786	0.791	−1.959	No/No	0.941	1.569	1.387
**28**	β-Bourbonene	1.395	95.668	−2.205	0.624	0.879	−1.218	No/No	0.967	1.601	1.431
**29**	β-Elemene	1.410	94.359	−1.279	0.601	0.809	−1.714	No/No	0.251	1.535	1.309
**30**	β-Caryophyllene	1.423	94.845	−1.580	0.652	0.733	−2.172	No/No	1.088	1.617	1.416
**31**	Seychellene	1.386	96.161	−2.249	0.787	0.866	−1.606	No/No	0.983	1.675	1.409
**32**	α-Guaiene	1.420	95.512	−1.538	0.682	0.763	−2.235	No/No	1.219	1.679	1.365
**33**	α-Humulene	1.421	94.682	−1.739	0.505	0.663	−2.555	No/No	1.282	1.766	1.336
**34**	Allo-Aromadendrene	1.395	95.302	−1.828	0.753	0.822	−1.769	No/No	0.926	1.526	1.332
**35**	α-Patchoulene	1.394	94.515	−1.833	0.751	0.818	−1.759	No/No	0.973	1.552	1.334
**36**	γ-Muurolene	1.427	96.475	−1.561	0.67	0.809	−1.631	No/No	1.188	1.540	1.473
**37**	Germacrene-D	1.436	95.59	−1.429	0.544	0.723	−2.138	No/No	1.420	1.634	1.413
**38**	Valencene	1.434	96.587	−1.473	0.692	0.779	−1.955	No/No	1.205	1.604	1.480
**39**	Eremophyllene	1.401	94.127	−1.461	0.686	0.776	−1.865	No/No	1.211	1.543	1.351
**40**	γ-Cadinene	1.427	96.475	−1.561	0.67	0.809	−1.631	No/No	1.188	1.540	1.473
**41**	7-epi-α-Selinene	1.373	94.846	−1.989	0.674	0.804	−3.226	No/No	1.183	1.912	1.129
**42**	δ-Cadinene	1.422	96.128	−1.462	0.689	0.773	−1.945	No/No	1.182	1.552	1.448
**43**	Spathulenol	1.388	93.235	−2.141	0.522	0.600	−2.447	No/No	0.895	1.687	1.390
**44**	β-Caryophyllene oxide	1.414	95.669	−3.061	0.564	0.647	−2.521	No/No	0.905	1.548	1.224
**45**	T-Cadinol	1.352	96.460	−2.285	0.543	0.565	−3.299	No/No	1.147	2.065	0.895
**46**	δ-Cadinol	1.479	94.296	−1.923	0.420	0.596	−2.151	No/No	1.085	1.918	1.475
**47**	Patchoulol	1.475	92.467	−2.397	0.668	0.649	−2.303	No/No	0.871	1.707	1.238

**Caco–2**: Caucasian colon adenocarcinoma permeability (Log Papp in 10^−6^cm/s). **IA**: intestinal absorption (% Absorbed). **SP**: skin permeability (logKp). **VDss**: steady state Volume of Distribution (Log L/kg). **BBB**: blood–brain barrier permeability (log BB). CNS: central nervous system (Log PS). **CYP2D6**: Cytochrome P450 2D6 inhibitor; **CYP3A4**: Cytochrome P450 3A4 inhibitor. **TC**: total clearance (Log mL/min/kg). **LD_50_**: lethal dose, 50% (mol/Kg). **LOAEL**: Lowest Observed Adverse Effect Level (Log mg/kg bw/day).

## Data Availability

The data presented in this study are available in the article.

## Supplementary Materials

The following supporting information can be downloaded at: https://www.mdpi.com/article/10.3390/antiox11071337/s1. Figure S1: Superposition of the co-crystal warfarin with its docked pose bound to CYP2C9. Figure S2: Superposition of the co-crystal quercetin with its docked pose bound to xanthine oxidase. Figure S3: Molecular docking visualization for the abundant compounds identified in the VPEO bound to CYP2C9 and xanthine oxidase. Table S1: Canonical SMILES of 47 *Valeriana pilosa* essential oils used for Ligand Efficiency studies. Table S2: Complete results for essential oils from *Valeriana pilosa* with CYP2C9 target: intermolecular docking energy values (∆*E_binding_*), *K_d_* values, Ligand Efficiency (*LE*), Binding Efficiency Index (*BEI*), and Lipophilic Ligand Efficiency (*LLE*). Table S3: Complete results for essential oils from *Valeriana pilosa* with catalase target: intermolecular docking energy values (∆*E_binding_*), *K_d_* values, Ligand Efficiency (*LE*), Binding Efficiency Index (*BEI*), and Lipophilic Ligand Efficiency (*LLE*). Table S4: Complete results for essential oils from *Valeriana pilosa* with superoxide dismutase target: intermolecular docking energy values (∆*E_binding_*), *K_d_* values, Ligand Efficiency (*LEB*), Binding Efficiency Index (*BEI*), and Lipophilic Ligand Efficiency (*LLE*). Table S5: Complete results for essential oils from *Valeriana pilosa* with xanthine oxidase target: intermolecular docking energy values (∆*E_binding_*), *K_d_* values, Ligand Efficiency (*LE*), Binding Efficiency Index (*BEI*), and Lipophilic Ligand Efficiency (*LLE*) and Table S6: Mol2 files for all compounds studied in this work.
