# Supplementary material for: Valeriana pilosa Roots Essential Oil: Chemical Composition, Antioxidant Activities, and Molecular Docking Studies on Enzymes Involved in Redox Biological Processes
Source: Antioxidants (Basel). 2022 Jul 7;11(7):1337. doi: 10.3390/antiox11071337 (PMC9311991; doi:10.3390/antiox11071337)
Supplement: Supplementary file 1 [file antioxidants-11-01337-s001.zip › antioxidants-1782575-supplementary.pdf]

SUPPLEMENTARY MATERIAL

Figure	Pag.
**Figure S1.** Superposition of the co-crystal warfarine (cyan color) with its docked pose (green color) bound to CYP2C9. Here, complex alignment was performed with respect to ligand and position conformation of the aligned ligands was calculated in terms of RMSD values.	S4
**Figure S2.** Superposition of the co-crystal quercetin (cyan color) with its docked pose (green color) bound to Xanthine Oxidase. Here, complex alignment was performed with respect to ligand and position conformation of the aligned ligands was calculated in terms of RMSD values.	S4
**Figure S3.** Molecular Docking visualization for the abundant compounds identified in the VPEO bound to CYP2C9 and Xanthine Oxidase.	S5
**Figure S4.** Heat map of the score normalization based on the number of non-Hydrogen Atoms values (kcal·mol^{-1}) of VPEO components.	S6
**Table**	**Pag.**
**Table S1.** Canonical SMILES of 47 *Valeriana pilosa* essential oils used for ligand efficiency studies.	S7 – S8
**Table S2.** Complete results for essential oils from *Valeriana pilosa* with CYP2C9 target: Intermolecular docking energy values ($\Delta E_{binding}$), Kd values,	S9 – S10

Ligand Efficiency (*LE*), Binding Efficiency Index (*BEI*), and Lipophilic Ligand Efficiency (*LLE*)	
**Table S3**. Complete results for essential oils from *Valeriana pilosa* with Catalase target: Intermolecular docking energy values ($\Delta E_{binding}$), *Kd* values, Ligand Efficiency (*LE*), Binding Efficiency Index (*BEI*), and Lipophilic Ligand Efficiency (*LLE*)	S11 – S12
**Table S4.** Complete results for essential oils from *Valeriana pilosa* with Superoxide Dismutase target: Intermolecular docking energy values ($\Delta E_{binding}$), *Kd* values, Ligand Efficiency (*LE*), Binding Efficiency Index (*BEI*), and Lipophilic Ligand Efficiency (*LLE*)	S13 – S14
**Table S5**. Complete results for essential oils from *Valeriana pilosa* with Xanthine Oxidase target: Intermolecular docking energy values ($\Delta E_{\text{binding}}$), *Kd* values, Ligand Efficiency (*LE*), Binding Efficiency Index (*BEI*), and Lipophilic Ligand Efficiency (*LLE*)	S15 – S16
**Table S6.** mol2 files for all compounds studied in this work.	S16 – S72

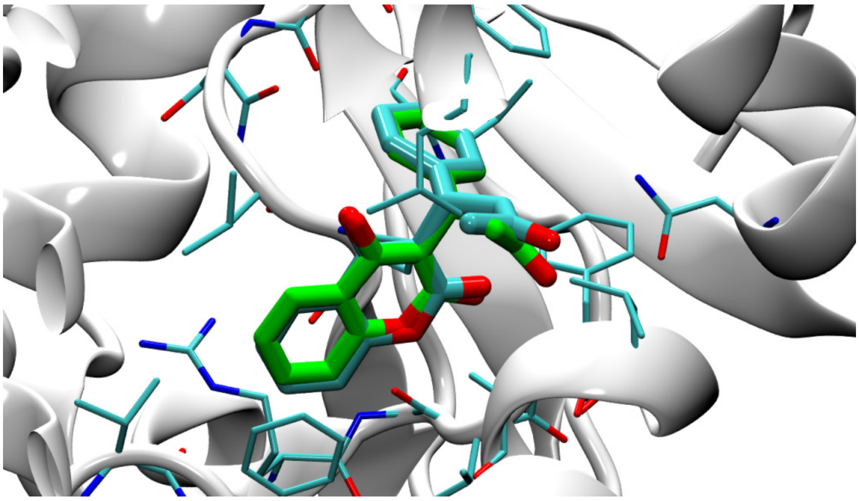

Figure S1. Superposition of the co-crystal warfarine (cyan color) with its docked pose (green color) bound to CYP2C9. Here, complex alignment was performed with respect to ligand and position conformation of the aligned ligands was calculated in terms of RMSD values.

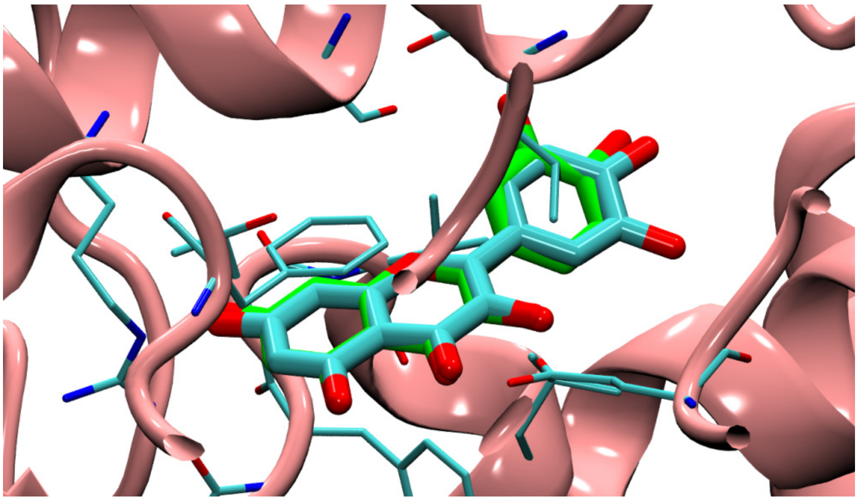

Figure S2. Superposition of the co-crystal quercetin (cyan color) with its docked pose (green color) bound to Xanthine Oxidase. Here, complex alignment was performed with respect to ligand and position conformation of the aligned ligands was calculated in terms of RMSD values.

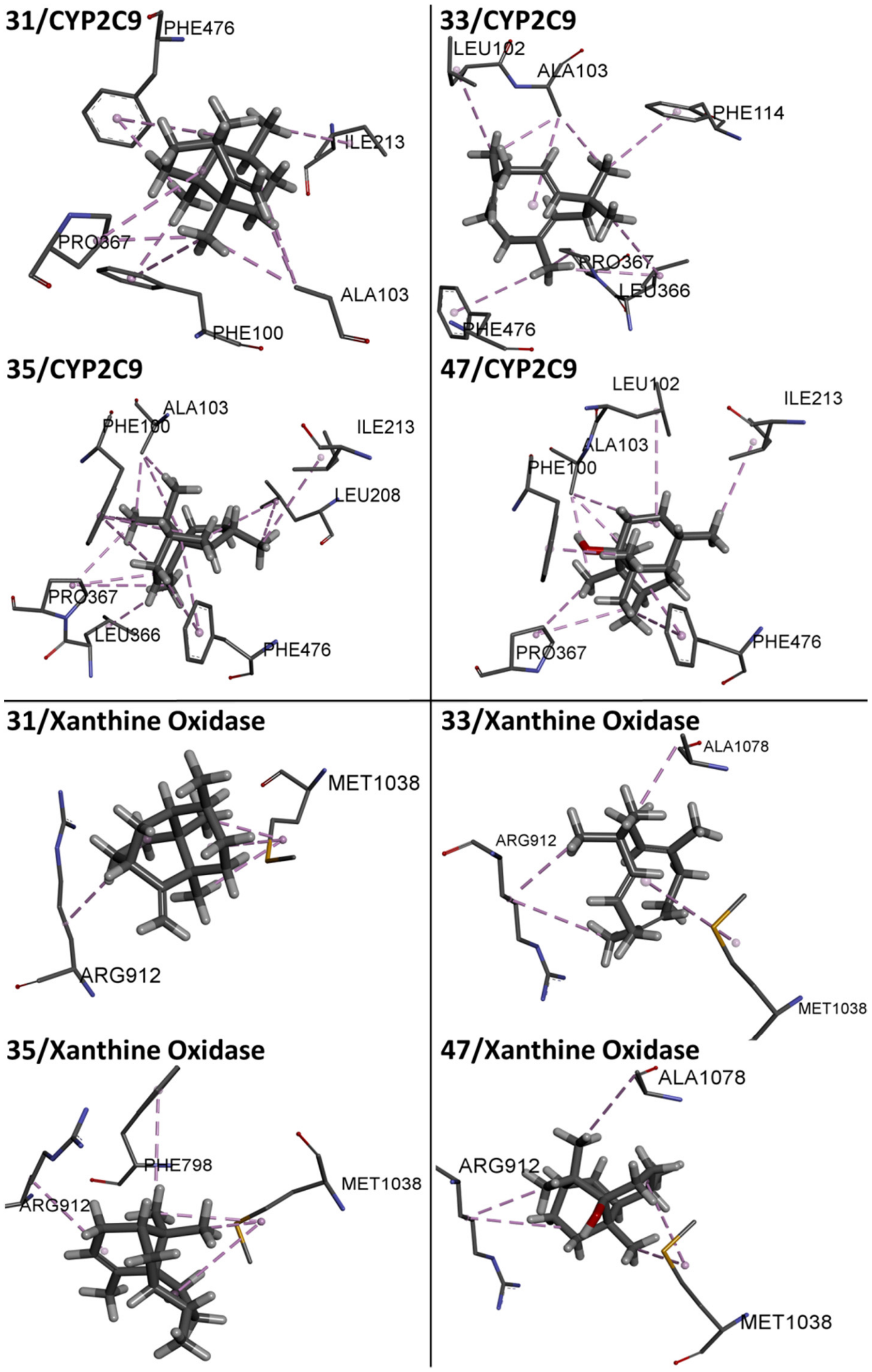

Figure S3. Molecular Docking visualization for the abundant compounds identified in the VPEO bound to CYP2C9 and Xanthine Oxidase.

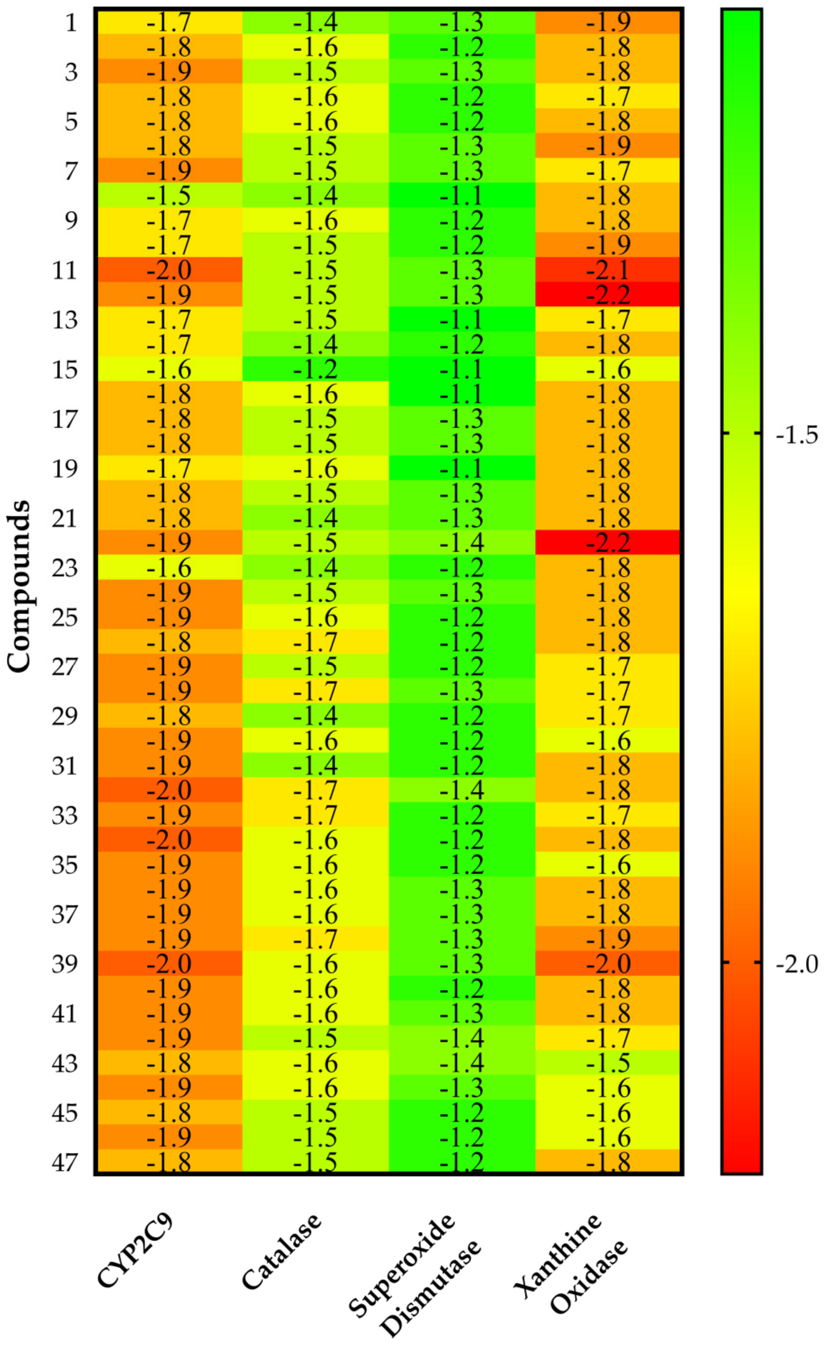

Figure S4. Heat map of the score normalization based on the number of non-Hydrogen Atoms values (kcal·mol^{-1}) of VPEO components.

Table S1. Canonical SMILES of 47 *Valeriana pilosa* essential oils used for ligand efficiency studies.

N°	Compound Name	Canonical SMILES
1	Isovaleric acid	CC(CC(=O)O)C
2	Tricyclene	C[C@]12[C@@H]3[C@H]1C[C@H](C2(C)C)C3
3	α-Thujene	CC1=CC[C@@]2([C@H]1C2)C(C)C
4	α-Pinene	CC1=CC[C@H]2C[C@@H]1C2(C)C
5	Camphene	C=C1[C@H]2CC[C@@H](C1(C)C)C2
6	3-Methyl valeric acid	C[C@H](CC)CC(=O)O
7	Sabinene	C=C1CC[C@@]2([C@H]1C2)C(C)C
8	1-Octen-3-ol	CCCCC[C@H](C=C)O
9	β-Pinene	C=C1CC[C@H]2C[C@@H]1C2(C)C
10	Myrcene	C=CC(=C)CCC=C(C)C
11	Limonene	CC1=CC[C@@H](CC1)C(=C)C
12	*p*-Cymene	Cc1ccc(cc1)C(C)C
13	1,8-Cineole	C[C@]12CC[C@H](CC1)C(O2)(C)C
14	Linalool	C=C[C@](CCC=C(C)C)(O)C
15	Isopentyl isovalerate	CC(CCOC(=O)CC(C)C)C
16	Camphor	O=C1C[C@H]2C([C@]1(C)CC2)(C)C
17	Menthone	C[C@@H]1CC[C@@H](C(=O)C1)C(C)C
18	Isomenthone	C[C@@H]1CC[C@@H](C(=O)C1)C(C)C
19	Borneol	O[C@@H]1C[C@H]2C([C@]1(C)CC2)(C)C
20	Neomenthol	C[C@H]1CC[C@H]([C@@H](C1)O)C(C)C
21	Menthol	C[C@@H]1CC[C@H]([C@H](C1)O)C(C)C
22	Carvone	CC(=C)[C@@H]1CC=C(C(=O)C1)C
23	Menthyl acetate	C[C@@H]1CC[C@H]([C@@H](C1)OC(=O)C)C(C)C
24	α-Cubebene	CC([C@H]1CC[C@H]([C@@]23[C@@H]1[C@@H]2C(=CC3)C)C)C
25	Cyclosativene	CC([C@H]1CC[C@@H]([C@@]23[C@@H]1[C@@H]1[C@@H]([C@]2 1C)C3)C)C
26	α-Copaene	C[C@@H]1CC[C@@]2([C@@H]3[C@H]1[C@H]2C(=CC3)C)C
27	β-Patchoulene	C[C@@H]1CCC2=C1C[C@H]1CC[C@@]2(C1(C)C)C
28	β-Bourbonene	CC([C@@H]1CC[C@@]2([C@H]1[C@@H]1C(=C)CC[C@H]21)C)C
29	β-Elemene	C=C[C@@]1(C)CC[C@H](C[C@H]1C(=C)C)C(=C)C
30	β-Caryophyllene	C[C@H]1CCCC(=C)[C@H]2[C@H](CC1)C(C2)(C)C
31	Seychellene	C[C@H]1CC[C@@]2([C@@]3([C@H]1C[C@H](C2=C)CC3)C)C
32	α-Guaiene	CC(=C)[C@@H]1CC[C@@H](C2=C(C1)[C@@H](C)CC2)C
33	α-Humulene	CC1=CCC(C)(C)C=CCC(=CCC1)C
34	allo-Aromadendrene	C[C@H]1CC[C@@H]2[C@H]1[C@@H]1[C@@H](C1(C)C)CCC2=C
35	α-Patchoulene	C[C@H]1CC[C@]23[C@@H]1C[C@@H](C3(C)C)CC=C2C

36	γ-Muurolene	CC1=C[C@H]2[C@@H](CC1)C(=C)CC[C@@H]2C(C)C
37	Germacrene-D	CC1=CCCC(=C)C=C[C@@H](CC1)C(C)C
38	Valencene	CC(=C)[C@@H]1CCC2=CCC[C@H]([C@]2(C1)C)C
39	Eremophyllene	CC(=C)[C@@H]1CC[C@@H]2[C@](C1)(C)C(=CCC2)C
40	γ-Cadinene	CC1=C[C@@H]2[C@H](CC1)C(=C)CC[C@@H]2C(C)C
41	7-epi-α-Selinene	CC1=CCC[C@]2([C@@H]1C[C@@H](CC2)C(=C)C)C
42	δ-Cadinene	CC1=C[C@H]2C(=C(C)CC[C@H]2C(C)C)CC1
43	Spathulenol	C=C1CC[C@H]2[C@@H]([C@@H]3[C@@H]1CC[C@]3(C)O)C2(C)C
44	β-Caryophyllene oxide	C=C1CC[C@@H]2O[C@@]2(CC[C@@H]2[C@@H]1CC2(C)C)C
45	T-Cadinol	CC1=C[C@H]2[C@@H](CC1)[C@@](C)(O)CC[C@H]2C(C)C
46	δ-Cadinol	CC1=C[C@H]2[C@@H](CC1)[C@@](C)(O)CC[C@H]2C(C)C
47	Patchoulol	C[C@H]1CC[C@@]2([C@@]3([C@H]1C[C@H](C2(C)C)CC3)C)O

Table S2. Complete results for essential oils from *Valeriana pilosa* with CYP2C9 target: Intermolecular docking energy values ($\Delta E_{binding}$), K_d values, Ligand Efficiency (*LE*), Binding Efficiency Index (*BEI*), and Lipophilic Ligand Efficiency (*LLE*)

Compound	$\Delta E_{binding}$ (kcal·mol⁻¹)	K_d	*LE* (kcal·mol⁻¹)	*BEI* (kDa)	*LLE*
Isovaleric acid	−4.6	4.25E-04	0.66	33.01	2.25
Tricyclene	−5.8	5.61E-05	0.58	31.20	1.56
α-Thujene	−6.1	3.38E-05	0.61	32.82	1.47
α-Pinene	−5.6	7.87E-05	0.56	30.13	1.11
Camphene	−5.8	5.61E-05	0.58	31.20	1.25
3-Methyl valeric acid	−5.1	1.83E-04	0.64	32.18	2.23
Sabinene	−5.9	4.74E-05	0.59	31.74	1.33
1-Octen-3-ol	−4.5	5.04E-04	0.50	25.72	1.18
β-Pinene	−5.5	9.32E-05	0.55	29.59	1.03
Myrcene	−5.5	9.32E-05	0.55	29.59	0.56
Limonene	−6.2	2.86E-05	0.62	33.35	1.23
p-Cymene	−6.1	3.38E-05	0.61	33.31	1.35
1,8-Cineole	−5.6	7.87E-05	0.51	26.61	1.36
Linalool	−5.6	7.87E-05	0.51	26.61	1.43
Isopentyl isovalerate	−5.5	9.32E-05	0.46	23.40	1.41
Camphor	−6.0	4.01E-05	0.55	28.88	2.00
Menthone	−6.0	4.01E-05	0.55	28.51	1.75
Isomenthone	−6.0	4.01E-05	0.55	28.51	1.75
Borneol	−5.7	6.65E-05	0.52	27.08	1.98
Neomenthol	−6.0	4.01E-05	0.55	28.14	1.96
Menthol	−5.9	4.74E-05	0.54	27.67	1.88
Carvone	−6.4	2.04E-05	0.58	31.22	2.20
Menthyl acetate	−6.1	3.38E-05	0.44	22.54	1.46
α-Cubebene	−7.4	3.77E-06	0.49	26.54	1.15
Cyclosativene	−7.5	3.19E-06	0.50	26.90	1.54
α-Copaene	−6.6	1.46E-05	0.51	27.44	1.20
β-Patchoulene	−7.4	3.77E-06	0.49	26.54	0.86
β-Bourbonene	−7.4	3.77E-06	0.49	26.54	1.15
β-Elemene	−7.0	7.41E-06	0.47	25.10	0.38
β-Caryophyllene	−7.3	4.47E-06	0.49	25.92	0.54
Seychellene	−7.3	4.47E-06	0.49	26.18	0.93
α-Guaiene	−7.7	2.27E-06	0.51	27.61	0.92
α-Humulene	−7.3	4.47E-06	0.49	26.18	0.31
allo-Aromadendrene	−7.6	2.69E-06	0.51	27.26	1.30

α-Patchoulene	−7.3	4.47E-06	0.49	26.18	0.93
γ-Muurolene	−7.4	3.77E-06	0.49	26.54	0.84
Germacrene-D	−7.5	3.19E-06	0.50	26.90	0.61
Valencene	−7.4	3.77E-06	0.49	26.54	0.70
Eremophyllene	−7.8	1.92E-06	0.52	27.97	0.99
γ-Cadinene	−7.5	3.19E-06	0.50	26.90	0.92
7-epi-α-Selinene	−7.3	4.47E-06	0.49	26.18	0.62
δ-Cadinene	−7.4	3.77E-06	0.49	26.54	0.70
Spathulenol	−7.2	5.29E-06	0.45	23.95	1.89
β-Caryophyllene oxide	−7.5	3.19E-06	0.47	24.94	1.56
T-Cadinol	−7.3	4.47E-06	0.46	24.06	1.57
δ-Cadinol	−7.4	3.77E-06	0.46	24.39	1.65
Patchoulol	−7.3	4.47E-06	0.46	24.06	1.74

Table S3. Complete results for essential oils from *Valeriana pilosa* with Catalase target: Intermolecular docking energy values ($\Delta E_{binding}$), K_d values, Ligand Efficiency (*LE*), Binding Efficiency Index (*BEI*), and Lipophilic Ligand Efficiency (*LLE*)

Compound	$\Delta E_{binding}$ (kcal·mol^{-1})	K_d	*LE* (kcal·mol^{-1})	*BEI* (kDa)	*LLE*
Isovaleric acid	−3.7	1.94E-03	0.53	26.55	1.59
Tricyclene	−5.1	1.83E-04	0.51	27.44	1.05
α-Thujene	−4.8	3.04E-04	0.48	25.82	0.52
α-Pinene	−5.0	2.17E-04	0.50	26.90	0.67
Camphene	−5.0	2.17E-04	0.50	26.90	0.67
3-Methyl valeric acid	−4.2	8.35E-04	0.53	26.50	1.57
Sabinene	−4.8	3.04E-04	0.48	25.82	0.52
1-Octen-3-ol	−4.1	9.89E-04	0.46	23.44	0.89
β-Pinene	−5.0	2.17E-04	0.50	26.90	0.67
Myrcene	−4.7	3.59E-04	0.47	25.28	-0.03
Limonene	−4.8	3.04E-04	0.48	25.82	0.21
p-Cymene	−4.8	3.04E-04	0.48	26.21	0.40
1,8-Cineole	−5.0	2.17E-04	0.45	23.76	0.92
Linalool	−4.6	4.25E-04	0.42	21.86	0.70
Isopentyl isovalerate	−4.2	8.35E-04	0.35	17.87	0.46
Camphor	−5.3	1.31E-04	0.48	25.51	1.48
Menthone	−5.1	1.83E-04	0.46	24.23	1.09
Isomenthone	−5.1	1.83E-04	0.46	24.23	1.09
Borneol	−5.2	1.55E-04	0.47	24.71	1.62
Neomenthol	−4.9	2.56E-04	0.45	22.98	1.15
Menthol	−4.8	3.04E-04	0.44	22.51	1.08
Carvone	−5.0	2.17E-04	0.45	24.39	1.18
Menthyl acetate	−5.2	1.55E-04	0.37	19.22	0.80
α-Cubebene	−5.9	4.74E-05	0.39	21.16	0.05
Cyclosativene	−6.2	2.86E-05	0.41	22.24	0.58
α-Copaene	−6.0	4.01E-05	0.46	24.94	0.76
β-Patchoulene	−6.0	4.01E-05	0.40	21.52	-0.16
β-Bourbonene	−6.5	1.72E-05	0.43	23.31	0.49
β-Elemene	−5.6	7.87E-05	0.37	20.08	-0.64
β-Caryophyllene	−6.1	3.38E-05	0.41	21.66	-0.33
Seychellene	−5.6	7.87E-05	0.37	20.08	-0.31
α-Guaiene	−6.4	2.04E-05	0.43	22.95	-0.03
α-Humulene	−6.4	2.04E-05	0.43	22.95	-0.35
allo-Aromadendrene	−6.2	2.86E-05	0.41	22.24	0.27

α-Patchoulene	−6.1	3.38E-05	0.41	21.88	0.06
γ-Muurolene	−6.2	2.86E-05	0.41	22.24	−0.04
Germacrene-D	−6.1	3.38E-05	0.41	21.88	−0.42
Valencene	−6.5	1.72E-05	0.43	23.31	0.04
Eremophyllene	−6.1	3.38E-05	0.41	21.88	−0.25
γ-Cadinene	−6.3	2.41E-05	0.42	22.59	0.04
7-epi-α-Selinene	−6.3	2.41E-05	0.42	22.59	−0.11
δ-Cadinene	−6.0	4.01E-05	0.40	21.52	−0.33
Spathulenol	−6.5	1.72E-05	0.41	21.62	1.38
β-Caryophyllene oxide	−6.5	1.72E-05	0.41	21.62	0.83
T-Cadinol	−5.9	4.74E-05	0.37	19.45	0.55
δ-Cadinol	−5.9	4.74E-05	0.37	19.45	0.55
Patchoulol	−5.9	4.74E-05	0.37	19.45	0.71

Table S4. Complete results for essential oils from *Valeriana pilosa* with Superoxide Dismutase target: Intermolecular docking energy values ($\Delta E_{binding}$), K_d values, Ligand Efficiency (*LE*B), inding Efficiency Index (*BEI*), and Lipophilic Ligand Efficiency (*LLE*)

Compound	$\Delta E_{binding}$ (kcal·mol⁻¹)	K_d	*LE* (kcal·mol⁻¹)	*BEI* (kDa)	*LLE*
Isovaleric acid	−3.5	2.72E-03	0.50	25.12	1.45
Tricyclene	−3.7	1.94E-03	0.37	19.90	0.02
α-Thujene	−4.1	9.89E-04	0.41	22.06	0.01
α-Pinene	−3.8	1.64E-03	0.38	20.44	−0.21
Camphene	−3.8	1.64E-03	0.38	20.44	−0.21
3-Methyl valeric acid	−3.7	1.94E-03	0.46	23.34	1.20
Sabinene	−4.1	9.89E-04	0.41	22.06	0.01
1-Octen-3-ol	−3.4	3.22E-03	0.38	19.43	0.38
β-Pinene	−3.8	1.64E-03	0.38	20.44	−0.21
Myrcene	−3.7	1.94E-03	0.37	19.90	−0.76
Limonene	−4.2	8.35E-04	0.42	22.59	−0.23
p-Cymene	−4.1	9.89E-04	0.41	22.39	−0.11
1,8-Cineole	−3.8	1.64E-03	0.35	18.05	0.04
Linalool	−3.9	1.39E-03	0.35	18.53	0.19
Isopentyl isovalerate	−3.9	1.39E-03	0.33	16.59	0.24
Camphor	−3.8	1.64E-03	0.35	18.29	0.38
Menthone	−4.2	8.35E-04	0.38	19.96	0.43
Isomenthone	−4.2	8.35E-04	0.38	19.96	0.43
Borneol	−3.8	1.64E-03	0.35	18.05	0.59
Neomenthol	−4.2	8.35E-04	0.38	19.70	0.64
Menthol	−4.3	7.06E-04	0.39	20.17	0.71
Carvone	−4.7	3.59E-04	0.43	22.93	0.96
Menthyl acetate	−4.6	4.25E-04	0.33	17.00	0.36
α-Cubebene	−5.0	2.17E-04	0.33	17.93	−0.61
Cyclosativene	−4.8	3.04E-04	0.32	17.21	−0.44
α-Copaene	−4.5	5.04E-04	0.35	18.71	−0.34
β-Patchoulene	−4.8	3.04E-04	0.32	17.21	−1.04

β-Bourbonene	−5.2	1.55E-04	0.35	18.65	−0.46
β-Elemene	−4.6	4.25E-04	0.31	16.50	−1.38
β-Caryophyllene	−4.7	3.59E-04	0.31	16.69	−1.36
Seychellene	−4.7	3.59E-04	0.31	16.86	−0.97
α-Guaiene	−5.5	9.32E-05	0.37	19.72	−0.69
α-Humulene	−4.6	4.25E-04	0.31	16.50	−1.66
allo-Aromadendrene	−4.8	3.04E-04	0.32	17.21	−0.75
α-Patchoulene	−4.5	5.04E-04	0.30	16.14	−1.12
γ-Muurolene	−5.2	1.55E-04	0.35	18.65	−0.77
Germacrene-D	−4.9	2.56E-04	0.33	17.57	−1.30
Valencene	−5.0	2.17E-04	0.33	17.93	−1.06
Eremophyllene	−5.1	1.83E-04	0.34	18.29	−0.99
γ-Cadinene	−4.8	3.04E-04	0.32	17.21	−1.06
7-epi-α-Selinene	−5.0	2.17E-04	0.33	17.93	−1.06
δ-Cadinene	−5.5	9.32E-05	0.37	19.72	−0.69
Spathulenol	−5.5	9.32E-05	0.34	18.29	0.64
β-Caryophyllene oxide	−5.2	1.55E-04	0.33	17.29	−0.13
T-Cadinol	−4.8	3.04E-04	0.30	15.82	−0.26
δ-Cadinol	−4.8	3.04E-04	0.30	15.82	−0.26
Patchoulol	−4.7	3.59E-04	0.29	15.49	−0.17

Table S5. Complete results for essential oils from *Valeriana pilosa* with Xanthine Oxidase target: Intermolecular docking energy values ($\Delta E_{binding}$), K_d values, Ligand Efficiency (*LE*), Binding Efficiency Index (*BEI*), and Lipophilic Ligand Efficiency (*LLE*)

Compound	$\Delta E_{binding}$ (kcal·mol⁻¹)	K_d	*LE* (kcal·mol⁻¹)	*BEI* (kDa)	*LLE*
Isovaleric acid	−5.0	2.17E-04	0.71	35.88	2.55
Tricyclene	−5.6	7.87E-05	0.56	30.13	1.42
α-Thujene	−5.6	7.87E-05	0.56	30.13	1.11
α-Pinene	−5.5	9.32E-05	0.55	29.59	1.03
Camphene	−5.7	6.65E-05	0.57	30.66	1.18
3-Methyl valeric acid	−5.3	1.31E-04	0.66	33.44	2.38
Sabinene	−5.5	9.32E-05	0.55	29.59	1.03
1-Octen-3-ol	−5.4	1.10E-04	0.60	30.87	1.84
β-Pinene	−5.6	7.87E-05	0.56	30.13	1.11
Myrcene	−6.1	3.38E-05	0.61	32.82	1.00
Limonene	−6.7	1.23E-05	0.67	36.04	1.60
p-Cymene	−7.0	7.41E-06	0.70	38.22	2.01
1,8-Cineole	−5.7	6.65E-05	0.52	27.08	1.43
Linalool	−6.0	4.01E-05	0.55	28.51	1.73
Isopentyl isovalerate	−5.6	7.87E-05	0.47	23.82	1.48
Camphor	−6.1	3.38E-05	0.55	29.37	2.07
Menthone	−6.1	3.38E-05	0.55	28.98	1.82
Isomenthone	−6.1	3.38E-05	0.55	28.98	1.82
Borneol	−6.0	4.01E-05	0.55	28.51	2.20
Neomenthol	−6.1	3.38E-05	0.55	28.61	2.03
Menthol	−5.9	4.74E-05	0.54	27.67	1.88
Carvone	−7.2	5.29E-06	0.65	35.13	2.79
Menthyl acetate	−6.6	1.46E-05	0.47	24.39	1.83
α-Cubebene	−7.0	7.41E-06	0.47	25.10	0.86
Cyclosativene	−7.0	7.41E-06	0.47	25.10	1.17
α-Copaene	−6.6	1.46E-05	0.51	27.44	1.20

β-Patchoulene	−6.6	1.46E-05	0.44	23.67	0.28
β-Bourbonene	−6.6	1.46E-05	0.44	23.67	0.57
β-Elemene	−6.5	1.72E-05	0.43	23.31	0.02
β-Caryophyllene	−6.1	3.38E-05	0.41	21.66	−0.33
Seychellene	−6.8	1.04E-05	0.45	24.39	0.57
α-Guaiene	−7.0	7.41E-06	0.47	25.10	0.40
α-Humulene	−6.5	1.72E-05	0.43	23.31	−0.27
allo-Aromadendrene	−7.0	7.41E-06	0.47	25.10	0.86
α-Patchoulene	−6.2	2.86E-05	0.41	22.24	0.13
γ-Muurolene	−6.9	8.77E-06	0.46	24.75	0.48
Germacrene-D	−6.8	1.04E-05	0.45	24.39	0.09
Valencene	−7.3	4.47E-06	0.49	26.18	0.62
Eremophyllene	−7.6	2.69E-06	0.51	27.26	0.84
γ-Cadinene	−6.8	1.04E-05	0.45	24.39	0.40
7-epi-α-Selinene	−7.1	6.26E-06	0.47	25.46	0.48
δ-Cadinene	−6.5	1.72E-05	0.43	23.31	0.04
Spathulenol	−6.1	3.38E-05	0.38	20.29	1.08
β-Caryophyllene oxide	−6.5	1.72E-05	0.41	21.62	0.83
T-Cadinol	−6.3	2.41E-05	0.39	20.76	0.84
δ-Cadinol	−6.3	2.41E-05	0.39	20.76	0.84
Patchoulol	−7.0	7.41E-06	0.44	23.07	1.52

Table S6. .mol2 files for all compounds studied in this work.

Compound 1

```
@<TRIPOS>MOLECULE
compuesto_1.out
 17 16 0 0 0
SMALL
MULLIKEN_CHARGES

@<TRIPOS>ATOM
      1 C        -11.9915    0.7844    0.2482 C.3     1  UNL1      -0.5082
      2 C        -10.7395   -0.1045    0.3425 C.3     1  UNL1       0.0630
      3 C         -9.4784    0.7163   -0.0274 C.3     1  UNL1      -0.4278
      4 C         -8.1768   -0.0237    0.1933 C.2     1  UNL1       0.6395
      5 O         -7.1808    0.5141   -0.5653 O.3     1  UNL1      -0.5153
      6 C        -10.8939   -1.3344   -0.5708 C.3     1  UNL1      -0.5064
      7 O         -7.9583   -0.9609    0.9228 O.2     1  UNL1      -0.5200
      8 H        -11.9483    1.6438    0.9599 H       1  UNL1       0.1533
      9 H        -12.1443    1.1612   -0.7794 H       1  UNL1       0.1557
     10 H        -12.9034    0.2139    0.5123 H       1  UNL1       0.1577
     11 H        -10.6382   -0.4536    1.3980 H       1  UNL1       0.1265
     12 H         -9.5728    1.0315   -1.0862 H       1  UNL1       0.1896
     13 H         -9.4255    1.6480    0.5912 H       1  UNL1       0.1859
     14 H         -6.3578    0.0111   -0.3942 H       1  UNL1       0.3337
     15 H        -11.7979   -1.9264   -0.2844 H       1  UNL1       0.1583
     16 H        -11.0005   -1.0392   -1.6312 H       1  UNL1       0.1516
     17 H        -10.0080   -1.9956   -0.5194 H       1  UNL1       0.1628
@<TRIPOS>BOND
     1    16     6    1
     2    12     3    1
     3     9     1    1
     4     6    17    1
     5     6    15    1
     6     6     2    1
     7     5    14    1
     8     5     4    1
     9     3     4    1
    10     3     2    1
    11     3    13    1
    12     4     7    2
    13     1     2    1
    14     1    10    1
    15     1     8    1
    16     2    11    1
```

Compound 2

```
@<TRIPOS>MOLECULE
compuesto_2.out
 26 28 0 0 0
SMALL
MULLIKEN_CHARGES

@<TRIPOS>ATOM
      1 C        -10.1093    0.8568    0.4249 C.3     1  UNL1      -0.1864
      2 C        -10.5753   -0.5750    0.1219 C.3     1  UNL1      -0.2730
      3 C         -9.3919   -0.9748   -0.8188 C.3     1  UNL1      -0.1189
      4 C         -8.1257   -0.6046    0.0610 C.3     1  UNL1       0.1815
      5 C         -8.5666    0.8584    0.3809 C.3     1  UNL1       0.0104
      6 C         -9.3773    1.3508   -0.8388 C.3     1  UNL1      -0.1862
      7 C         -9.4367    0.1966   -1.8517 C.3     1  UNL1      -0.2726
      8 C         -7.7263    1.7576    1.2218 C.3     1  UNL1      -0.4703
      9 C         -6.8189   -0.6776   -0.7293 C.3     1  UNL1      -0.5159
     10 C         -8.0014   -1.4769    1.3113 C.3     1  UNL1      -0.5161
     11 H        -10.6946    1.4837    1.0734 H       1  UNL1       0.1445
```

12 H -10.6469 -1.2042 1.0172 H 1 UNL1 0.1352
13 H -11.5514 -0.6040 -0.3801 H 1 UNL1 0.1347
14 H -9.4122 -1.9831 -1.2236 H 1 UNL1 0.1242
15 H -9.3854 2.3678 -1.1855 H 1 UNL1 0.1443
16 H -10.3622 0.2056 -2.4432 H 1 UNL1 0.1347
17 H -8.5993 0.1924 -2.5599 H 1 UNL1 0.1351
18 H -7.5566 1.3299 2.2177 H 1 UNL1 0.1574
19 H -6.7411 1.9254 0.7694 H 1 UNL1 0.1571
20 H -8.1921 2.7408 1.3604 H 1 UNL1 0.1528
21 H -6.7844 0.0598 -1.5396 H 1 UNL1 0.1585
22 H -5.9554 -0.4826 -0.0846 H 1 UNL1 0.1530
23 H -6.6853 -1.6703 -1.1713 H 1 UNL1 0.1523
24 H -8.8290 -1.3156 2.0112 H 1 UNL1 0.1585
25 H -7.9886 -2.5404 1.0522 H 1 UNL1 0.1520
26 H -7.0718 -1.2597 1.8491 H 1 UNL1 0.1532
@<TRIPOS>BOND
1 17 7 1
2 16 7 1
3 7 6 1
4 7 3 1
5 21 9 1
6 14 3 1
7 15 6 1
8 23 9 1
9 6 5 1
10 6 1 1
11 3 4 1
12 3 2 1
13 9 22 1
14 9 4 1
15 13 2 1
16 4 5 1
17 4 10 1
18 2 1 1
19 2 12 1
20 5 1 1
21 5 8 1
22 1 11 1
23 19 8 1
24 25 10 1
25 8 20 1
26 8 18 1
27 10 26 1
28 10 24 1

Compound 3

@<TRIPOS>MOLECULE
compuesto_3.out
26 27 0 0 0
SMALL
MULLIKEN_CHARGES

@<TRIPOS>ATOM
1 C -8.6021 0.3558 -5.4823 C.3 1 UNL1 0.0319
2 C -8.0494 -1.0659 -5.2292 C.3 1 UNL1 -0.2026
3 C -8.7184 -0.2830 -4.1032 C.3 1 UNL1 -0.3555
4 C -9.8269 0.1762 -6.4004 C.3 1 UNL1 -0.2675
5 C -9.9559 -1.3235 -6.5783 C.2 1 UNL1 -0.2511
6 C -8.9726 -2.0031 -5.9569 C.2 1 UNL1 0.0926
7 C -8.7793 -3.4700 -5.8888 C.3 1 UNL1 -0.4903
8 C -7.7553 1.6014 -5.7079 C.3 1 UNL1 -0.0004
9 C -8.5069 2.8192 -5.1573 C.3 1 UNL1 -0.4893
10 C -6.3653 1.5076 -5.0642 C.3 1 UNL1 -0.4909
11 H -6.9963 -1.3109 -5.1913 H 1 UNL1 0.1538
12 H -9.6919 -0.6071 -3.7417 H 1 UNL1 0.1605
13 H -8.1106 0.0941 -3.2871 H 1 UNL1 0.1540

```
14 H -10.7379 0.6105 -5.9536 H 1 UNL1 0.1455
15 H -9.6766 0.6768 -7.3718 H 1 UNL1 0.1438
16 H -10.7763 -1.7340 -7.1370 H 1 UNL1 0.1486
17 H -9.5657 -4.0209 -6.4228 H 1 UNL1 0.1603
18 H -7.8178 -3.7783 -6.3200 H 1 UNL1 0.1644
19 H -8.7946 -3.8198 -4.8417 H 1 UNL1 0.1671
20 H -7.6154 1.7263 -6.8137 H 1 UNL1 0.1207
21 H -7.9455 3.7437 -5.3231 H 1 UNL1 0.1476
22 H -9.4890 2.9353 -5.6267 H 1 UNL1 0.1492
23 H -8.6730 2.7246 -4.0774 H 1 UNL1 0.1550
24 H -5.7898 2.4246 -5.2405 H 1 UNL1 0.1495
25 H -6.4303 1.3664 -3.9791 H 1 UNL1 0.1527
26 H -5.7816 0.6745 -5.4680 H 1 UNL1 0.1503
@<TRIPOS>BOND
1 15 4 1
2 16 5 1
3 20 8 1
4 5 4 1
5 5 6 2
6 17 7 1
7 4 14 1
8 4 1 1
9 18 7 1
10 6 7 1
11 6 2 1
12 7 19 1
13 8 1 1
14 8 9 1
15 8 10 1
16 22 9 1
17 1 2 1
18 1 3 1
19 26 10 1
20 21 9 1
21 24 10 1
22 2 11 1
23 2 3 1
24 9 23 1
25 10 25 1
26 3 12 1
27 3 13 1
```

Compound 4

```
@<TRIPOS>MOLECULE
compuesto_4.out
 26 27 0 0 0
SMALL
MULLIKEN_CHARGES

@<TRIPOS>ATOM
1 C -10.1088 0.8924 -5.9739 C.3 1 UNL1 -0.1658
2 C -10.3430 -0.5394 -6.6127 C.3 1 UNL1 0.1866
3 C -9.7164 -1.0831 -5.2653 C.3 1 UNL1 -0.1235
4 C -8.1766 -1.0078 -5.3088 C.3 1 UNL1 -0.2545
5 C -7.7292 0.3434 -5.8245 C.2 1 UNL1 -0.2406
6 C -8.6499 1.2731 -6.1472 C.2 1 UNL1 0.0767
7 C -10.2343 0.2148 -4.5709 C.3 1 UNL1 -0.3041
8 C -11.8284 -0.8898 -6.7259 C.3 1 UNL1 -0.5242
9 C -9.6353 -0.8699 -7.9177 C.3 1 UNL1 -0.5175
10 C -8.3444 2.6356 -6.6623 C.3 1 UNL1 -0.4981
11 H -10.8268 1.6743 -6.2270 H 1 UNL1 0.1324
12 H -10.0925 -2.0403 -4.8989 H 1 UNL1 0.1219
13 H -7.7625 -1.1735 -4.2946 H 1 UNL1 0.1358
14 H -7.7649 -1.8164 -5.9393 H 1 UNL1 0.1354
15 H -6.6618 0.4867 -5.9166 H 1 UNL1 0.1414
16 H -9.5999 0.6335 -3.7867 H 1 UNL1 0.1425
```

```
    17 H        -11.2570   0.1518   -4.1899 H      1  UNL1        0.1324
    18 H        -12.4292   -0.4162   -5.9419 H     1  UNL1        0.1552
    19 H        -12.2455   -0.5616   -7.6843 H     1  UNL1        0.1545
    20 H        -11.9757   -1.9728   -6.6538 H     1  UNL1        0.1557
    21 H         -9.0985   -1.8221   -7.8456 H     1  UNL1        0.1564
    22 H        -10.3291   -0.9480   -8.7613 H     1  UNL1        0.1506
    23 H         -8.9040   -0.1035   -8.1965 H     1  UNL1        0.1654
    24 H         -7.3166   2.7276   -7.0361 H      1  UNL1        0.1592
    25 H         -9.0231   2.9150   -7.4796 H      1  UNL1        0.1618
    26 H         -8.4729   3.3893   -5.8724 H      1  UNL1        0.1644
@<TRIPOS>BOND
     1    22     9    1
     2    23     9    1
     3     9    21    1
     4     9     2    1
     5    19     8    1
     6    25    10    1
     7    24    10    1
     8     8    20    1
     9     8     2    1
    10     8    18    1
    11    10     6    1
    12    10    26    1
    13     2     1    1
    14     2     3    1
    15    11     1    1
    16     6     1    1
    17     6     5    2
    18     1     7    1
    19    14     4    1
    20    15     5    1
    21     5     4    1
    22     4     3    1
    23     4    13    1
    24     3    12    1
    25     3     7    1
    26     7    17    1
    27     7    16    1
```

Compound 5

```
@<TRIPOS>MOLECULE
compuesto_5.out
 26 27 0 0 0
SMALL
MULLIKEN_CHARGES

@<TRIPOS>ATOM
      1 C         -9.2218   -0.0323   -7.1842 C.3    1  UNL1       -0.2753
      2 C         -9.5794   -0.4504   -5.7329 C.3    1  UNL1       -0.1015
      3 C         -8.2625   -0.8132   -4.9451 C.3    1  UNL1        0.1549
      4 C         -7.5910   0.5695   -4.8322 C.2     1  UNL1        0.0488
      5 C         -8.4978   1.3314   -7.0003 C.3     1  UNL1       -0.2668
      6 C         -8.5582   1.5680   -5.4613 C.3     1  UNL1       -0.0927
      7 C         -9.9351   0.9320   -5.1073 C.3     1  UNL1       -0.3052
      8 C         -8.6299   -1.3371   -3.5480 C.3    1  UNL1       -0.5113
      9 C         -7.3848   -1.8339   -5.6800 C.3    1  UNL1       -0.5075
     10 C         -6.4134   0.8478   -4.2826 C.2     1  UNL1       -0.4329
     11 H         -8.5758   -0.7625   -7.6900 H      1  UNL1        0.1336
     12 H        -10.1276   0.0653   -7.8015 H       1  UNL1        0.1276
     13 H        -10.3509   -1.2231   -5.6618 H      1  UNL1        0.1215
     14 H         -9.0037   2.1414   -7.5445 H       1  UNL1        0.1286
     15 H         -7.4577   1.2862   -7.3589 H       1  UNL1        0.1343
     16 H         -8.4194   2.6109   -5.1560 H       1  UNL1        0.1271
     17 H        -10.1475   0.8923   -4.0339 H       1  UNL1        0.1362
     18 H        -10.7805   1.4360   -5.5888 H       1  UNL1        0.1371
     19 H         -9.0988   -0.5567   -2.9347 H      1  UNL1        0.1568
```

```
   20 H        -9.3324  -2.1719  -3.6252 H      1  UNL1       0.1521
   21 H        -7.7485  -1.6908  -3.0009 H      1  UNL1       0.1554
   22 H        -6.7190  -1.3450  -6.4064 H      1  UNL1       0.1590
   23 H        -6.7434  -2.3867  -4.9844 H      1  UNL1       0.1537
   24 H        -7.9890  -2.5725  -6.2218 H      1  UNL1       0.1530
   25 H        -5.9955   1.8392  -4.2277 H      1  UNL1       0.1554
   26 H        -5.7694   0.0925  -3.8512 H      1  UNL1       0.1581
@<TRIPOS>BOND
  1   12    1   1
  2   11    1   1
  3   14    5   1
  4   15    5   1
  5    1    5   1
  6    1    2   1
  7    5    6   1
  8   22    9   1
  9   24    9   1
 10    2   13   1
 11    2    7   1
 12    2    3   1
 13    9   23   1
 14    9    3   1
 15   18    7   1
 16    6   16   1
 17    6    7   1
 18    6    4   1
 19    7   17   1
 20    3    4   1
 21    3    8   1
 22    4   10   2
 23   10   25   1
 24   10   26   1
 25   20    8   1
 26    8   21   1
 27    8   19   1
```

Compound 6

```
@<TRIPOS>MOLECULE
compuesto_6.out
 20 19 0 0 0
SMALL
MULLIKEN_CHARGES

@<TRIPOS>ATOM
    1 C        -6.8697   0.7602  -8.9193 C.3    1  UNL1      -0.2665
    2 C        -6.2783  -0.1072  -7.7830 C.3    1  UNL1       0.0216
    3 C        -5.2969   0.7401  -6.9274 C.3    1  UNL1      -0.4310
    4 C        -4.8102   0.0533  -5.6780 C.2    1  UNL1       0.6417
    5 O        -3.6247   0.5788  -5.2592 O.3    1  UNL1      -0.5155
    6 C        -5.5720  -1.3590  -8.3258 C.3    1  UNL1      -0.4962
    7 O        -5.3403  -0.8414  -5.0554 O.2    1  UNL1      -0.5218
    8 C        -7.9589   0.0555  -9.7322 C.3    1  UNL1      -0.4490
    9 H        -7.3110   1.6822  -8.4841 H      1  UNL1       0.1298
   10 H        -6.0451   1.1009  -9.5804 H      1  UNL1       0.1337
   11 H        -7.1221  -0.4252  -7.1221 H      1  UNL1       0.1320
   12 H        -4.4338   1.0281  -7.5664 H      1  UNL1       0.1898
   13 H        -5.7759   1.6814  -6.5966 H      1  UNL1       0.1833
   14 H        -3.3475   0.1058  -4.4421 H      1  UNL1       0.3355
   15 H        -5.1221  -1.9490  -7.5235 H      1  UNL1       0.1586
   16 H        -6.2824  -2.0330  -8.8377 H      1  UNL1       0.1582
   17 H        -4.7685  -1.0678  -9.0382 H      1  UNL1       0.1524
   18 H        -7.5628  -0.7973 -10.3124 H      1  UNL1       0.1493
   19 H        -8.7680  -0.3188  -9.0780 H      1  UNL1       0.1494
   20 H        -8.4099   0.7550 -10.4571 H      1  UNL1       0.1447
@<TRIPOS>BOND
  1   20    8   1
```

```
   2   18    8   1
   3    8   19   1
   4    8    1   1
   5   10    1   1
   6   17    6   1
   7    1    9   1
   8    1    2   1
   9   16    6   1
  10    6    2   1
  11    6   15   1
  12    2   11   1
  13    2    3   1
  14   12    3   1
  15    3   13   1
  16    3    4   1
  17    4    5   1
  18    4    7   2
  19    5   14   1
```

Compound 7

```
@<TRIPOS>MOLECULE
compuesto_7.out
 26 27 0 0 0
SMALL
MULLIKEN_CHARGES

@<TRIPOS>ATOM
      1 C          -7.2629    0.2711   -9.1354 C.3     1  UNL1        0.0352
      2 C          -6.8701   -1.1366   -8.6363 C.3     1  UNL1       -0.2148
      3 C          -7.8943   -0.3015   -7.8769 C.3     1  UNL1       -0.3478
      4 C          -8.0658    0.0888  -10.4293 C.3     1  UNL1       -0.2722
      5 C          -8.3819   -1.4228  -10.5351 C.3     1  UNL1       -0.3072
      6 C          -7.4076   -2.1314   -9.6125 C.2     1  UNL1        0.1536
      7 C          -7.0500   -3.4106   -9.6941 C.2     1  UNL1       -0.4658
      8 C          -6.3870    1.5158   -9.0840 C.3     1  UNL1       -0.0018
      9 C          -7.2640    2.7545   -8.8527 C.3     1  UNL1       -0.4886
     10 C          -5.2980    1.4174   -8.0084 C.3     1  UNL1       -0.4919
     11 H          -5.9069   -1.3455   -8.1785 H       1  UNL1        0.1591
     12 H          -8.9407   -0.5954   -7.8757 H       1  UNL1        0.1594
     13 H          -7.6180    0.1106   -6.9124 H       1  UNL1        0.1555
     14 H          -8.9716    0.7133  -10.4261 H       1  UNL1        0.1386
     15 H          -7.4678    0.4136  -11.2995 H       1  UNL1        0.1393
     16 H          -8.2912   -1.7781  -11.5749 H       1  UNL1        0.1471
     17 H          -9.4179   -1.6388  -10.2064 H       1  UNL1        0.1502
     18 H          -6.3600   -3.8787   -9.0115 H       1  UNL1        0.1634
     19 H          -7.4201   -4.0886  -10.4445 H       1  UNL1        0.1607
     20 H          -5.8754    1.6210  -10.0782 H       1  UNL1        0.1206
     21 H          -6.6681    3.6731   -8.8338 H       1  UNL1        0.1488
     22 H          -8.0113    2.8690   -9.6447 H       1  UNL1        0.1483
     23 H          -7.8063    2.6900   -7.9018 H       1  UNL1        0.1553
     24 H          -4.6882    2.3261   -7.9767 H       1  UNL1        0.1502
     25 H          -5.7267    1.2807   -7.0095 H       1  UNL1        0.1532
     26 H          -4.6222    0.5760   -8.1968 H       1  UNL1        0.1517
@<TRIPOS>BOND
   1   16    5   1
   2   15    4   1
   3    5    4   1
   4    5   17   1
   5    5    6   1
   6   19    7   1
   7    4   14   1
   8    4    1   1
   9   20    8   1
  10    7    6   2
  11    7   18   1
  12   22    9   1
```

```
  13    6    2   1
  14    1    8   1
  15    1    2   1
  16    1    3   1
  17    8    9   1
  18    8   10   1
  19    9   21   1
  20    9   23   1
  21    2   11   1
  22    2    3   1
  23   26   10   1
  24   10   24   1
  25   10   25   1
  26    3   12   1
  27    3   13   1
```

Compound 8

```
@<TRIPOS>MOLECULE
compuesto_8.out
 25 24 0 0 0
SMALL
MULLIKEN_CHARGES

@<TRIPOS>ATOM
     1 O        -9.4109    0.9451  -12.0316 O.3     1  UNL1      -0.5625
     2 H        -3.8677    1.0715   -6.7700 H       1  UNL1       0.1470
     3 H        -5.1830    1.7052   -5.7575 H       1  UNL1       0.1475
     4 H        -6.6122   -0.2993   -6.3315 H       1  UNL1       0.1265
     5 H        -5.3062   -0.9461   -7.3674 H       1  UNL1       0.1269
     6 H        -6.7630    1.7504   -7.7971 H       1  UNL1       0.1351
     7 H        -5.4579    1.1033   -8.8337 H       1  UNL1       0.1345
     8 H        -8.1847   -0.2628   -8.3492 H       1  UNL1       0.1326
     9 H        -6.8783   -0.9133   -9.3787 H       1  UNL1       0.1359
    10 H        -8.2967    1.7977   -9.8110 H       1  UNL1       0.1578
    11 H        -7.0209    1.1385  -10.8563 H       1  UNL1       0.1556
    12 H        -9.7903   -0.1572  -10.3508 H       1  UNL1       0.1097
    13 H        -9.9068    0.3882  -12.6656 H       1  UNL1       0.3118
    14 C        -9.0786   -2.2884  -11.7630 C.2     1  UNL1      -0.3212
    15 C        -8.3715   -1.1602  -11.6594 C.2     1  UNL1      -0.1967
    16 C        -4.7424    0.7798   -6.1685 C.3     1  UNL1      -0.4635
    17 C        -5.7687   -0.0077   -6.9910 C.3     1  UNL1      -0.2218
    18 C        -6.3036    0.8125   -8.1786 C.3     1  UNL1      -0.2667
    19 C        -7.3362    0.0262   -9.0040 C.3     1  UNL1      -0.2548
    20 C        -7.8610    0.8522  -10.1914 C.3     1  UNL1      -0.3167
    21 C        -8.9128    0.0810  -10.9928 C.3     1  UNL1       0.1756
    22 H        -4.3771    0.1733   -5.3236 H       1  UNL1       0.1449
    23 H        -7.3691   -1.0705  -12.0840 H       1  UNL1       0.1576
    24 H        -8.6697   -3.1664  -12.2427 H       1  UNL1       0.1535
    25 H       -10.0866   -2.3990  -11.3775 H       1  UNL1       0.1514
@<TRIPOS>BOND
   1   13    1   1
   2   24   14   1
   3   23   15   1
   4    1   21   1
   5   14   15   2
   6   14   25   1
   7   15   21   1
   8   21   12   1
   9   21   20   1
  10   11   20   1
  11   20   10   1
  12   20   19   1
  13    9   19   1
  14   19    8   1
  15   19   18   1
  16    7   18   1
```

```
  17   18    6   1
  18   18   17   1
  19    5   17   1
  20   17    4   1
  21   17   16   1
  22    2   16   1
  23   16    3   1
  24   16   22   1
```

Compound 9

```
@<TRIPOS>MOLECULE
compuesto_9.out
 26 27 0 0 0
SMALL
MULLIKEN_CHARGES

@<TRIPOS>ATOM
      1 C          -9.0296    0.9557  -10.7318 C.3     1  UNL1      -0.1793
      2 H          -6.3090    3.1143  -10.2393 H       1  UNL1       0.1590
      3 H          -7.8881    3.4767  -11.0141 H       1  UNL1       0.1616
      4 H          -7.3919    0.2133  -12.7450 H       1  UNL1       0.1627
      5 H          -7.9459   -1.4226  -13.1051 H       1  UNL1       0.1524
      6 H          -6.8610   -1.1689  -11.7596 H       1  UNL1       0.1595
      7 H         -10.4095   -2.0017  -11.7966 H       1  UNL1       0.1556
      8 H         -10.1376   -0.8898  -13.1552 H       1  UNL1       0.1563
      9 H         -11.0759   -0.3674  -11.7642 H       1  UNL1       0.1555
     10 H         -10.7252    0.1900   -9.4708 H       1  UNL1       0.1348
     11 H          -9.3574    0.7457   -8.5034 H       1  UNL1       0.1429
     12 H          -5.8842    0.2803  -10.2892 H       1  UNL1       0.1458
     13 H          -6.3592    0.9064   -8.7087 H       1  UNL1       0.1441
     14 H          -6.8138   -1.7253   -9.6952 H       1  UNL1       0.1290
     15 H          -7.4727   -1.0360   -8.2066 H       1  UNL1       0.1307
     16 H          -9.3385   -1.9486   -9.6758 H       1  UNL1       0.1227
     17 H          -9.6472    1.6827  -11.2684 H       1  UNL1       0.1384
     18 C          -7.2714    2.7302  -10.5378 C.2     1  UNL1      -0.4862
     19 C          -7.7162   -0.7302  -12.2881 C.3     1  UNL1      -0.5192
     20 C         -10.1981   -0.9551  -12.0608 C.3     1  UNL1      -0.5238
     21 C          -9.6357    0.2926   -9.4583 C.3     1  UNL1      -0.3020
     22 C          -7.6641    1.4689  -10.3334 C.2     1  UNL1       0.1526
     23 C          -6.7655    0.4611   -9.6399 C.3     1  UNL1      -0.3030
     24 C          -7.4351   -0.8981   -9.3044 C.3     1  UNL1      -0.2578
     25 C          -8.8540   -0.9889   -9.8766 C.3     1  UNL1      -0.1225
     26 C          -8.9094   -0.4848  -11.3739 C.3     1  UNL1       0.1903
@<TRIPOS>BOND
     1    8   20    1
     2    5   19    1
     3    4   19    1
     4   19    6    1
     5   19   26    1
     6   20    7    1
     7   20    9    1
     8   20   26    1
     9   26    1    1
    10   26   25    1
    11   17    1    1
    12    3   18    1
    13    1   22    1
    14    1   21    1
    15   18   22    2
    16   18    2    1
    17   22   23    1
    18   12   23    1
    19   25   16    1
    20   25   21    1
    21   25   24    1
    22   14   24    1
```

```
   23   23   24   1
   24   23   13   1
   25   10   21   1
   26   21   11   1
   27   24   15   1
```

Compound 10

```
@<TRIPOS>MOLECULE
compuesto_10.out
 26 25 0 0 0
SMALL
MULLIKEN_CHARGES

@<TRIPOS>ATOM
      1 C          -8.9510    0.4149  -11.4624 C.2     1  UNL1       -0.3134
      2 H          -4.4464   -1.6289   -4.2633 H       1  UNL1        0.1618
      3 H          -3.0511   -1.6234   -5.3524 H       1  UNL1        0.1618
      4 H          -4.6598   -1.9887   -5.9779 H       1  UNL1        0.1597
      5 H          -8.0760   -1.9405  -10.3402 H       1  UNL1        0.1565
      6 H          -6.9810   -2.0782   -8.9238 H       1  UNL1        0.1569
      7 H          -2.4908    0.8285   -4.6270 H       1  UNL1        0.1619
      8 H          -3.8827    0.8256   -3.5361 H       1  UNL1        0.1620
      9 H          -3.7357    2.0710   -4.7801 H       1  UNL1        0.1584
     10 H          -5.1772    1.6946   -6.6176 H       1  UNL1        0.1503
     11 H          -6.5634   -0.8701   -6.9527 H       1  UNL1        0.1407
     12 H          -5.1635   -0.8684   -8.0496 H       1  UNL1        0.1406
     13 H          -7.3206    1.3400   -7.9212 H       1  UNL1        0.1513
     14 H          -5.9212    1.3444   -9.0106 H       1  UNL1        0.1513
     15 H          -8.0274    1.8812  -10.2720 H       1  UNL1        0.1413
     16 H          -9.1435   -0.6187  -11.7133 H       1  UNL1        0.1517
     17 H          -9.4613    1.1117  -12.1135 H       1  UNL1        0.1473
     18 C          -4.1128   -1.3522   -5.2727 C.3     1  UNL1       -0.5009
     19 C          -7.4987   -1.4124   -9.5952 C.2     1  UNL1       -0.4076
     20 C          -3.5725    1.0065   -4.5753 C.3     1  UNL1       -0.5041
     21 C          -4.3145    0.1102   -5.5237 C.2     1  UNL1        0.1288
     22 C          -5.0825    0.6147   -6.5012 C.2     1  UNL1       -0.2763
     23 C          -5.8565   -0.2020   -7.4928 C.3     1  UNL1       -0.2210
     24 C          -6.6350    0.6724   -8.4871 C.3     1  UNL1       -0.3095
     25 C          -7.4405   -0.0779   -9.5222 C.2     1  UNL1        0.1003
     26 C          -8.1681    0.8106  -10.4568 C.2     1  UNL1       -0.1497
@<TRIPOS>BOND
    1   17    1   1
    2   16    1   1
    3    1   26   2
    4   26   15   1
    5   26   25   1
    6    5   19   1
    7   19   25   2
    8   19    6   1
    9   25   24   1
   10   14   24   1
   11   24   13   1
   12   24   23   1
   13   12   23   1
   14   23   11   1
   15   23   22   1
   16   10   22   1
   17   22   21   2
   18    4   18   1
   19   21   18   1
   20   21   20   1
   21    3   18   1
   22   18    2   1
   23    9   20   1
   24    7   20   1
   25   20    8   1
```

Compound 11

```
@<TRIPOS>MOLECULE
compuesto_11.out
 26 26 0 0 0
SMALL
MULLIKEN_CHARGES

@<TRIPOS>ATOM
      1 C       -11.1593   -0.0043  -14.3008 C.3     1  UNL1       -0.2560
      2 H        -9.2875    4.0841  -12.8206 H       1  UNL1        0.1620
      3 H        -8.2822    2.8516  -13.6067 H       1  UNL1        0.1626
      4 H        -8.7395    2.6754  -11.8802 H       1  UNL1        0.1638
      5 H       -12.4037    2.3047  -13.7563 H       1  UNL1        0.1601
      6 H       -11.6011    3.9067  -13.3855 H       1  UNL1        0.1564
      7 H       -10.0298   -4.0939  -12.2815 H       1  UNL1        0.1589
      8 H        -9.5634   -3.9769  -13.9972 H       1  UNL1        0.1614
      9 H       -11.2959   -4.0882  -13.5292 H       1  UNL1        0.1618
     10 H        -9.1239    0.5426  -13.8626 H       1  UNL1        0.1362
     11 H        -9.2864    0.3886  -11.4166 H       1  UNL1        0.1333
     12 H       -11.0791    0.3516  -11.4560 H       1  UNL1        0.1454
     13 H        -9.8959   -1.9891  -11.1582 H       1  UNL1        0.1439
     14 H       -11.5853   -2.0151  -14.9971 H       1  UNL1        0.1418
     15 H        -9.8649   -1.5353  -15.1798 H       1  UNL1        0.1446
     16 H       -12.1671    0.0227  -13.8294 H       1  UNL1        0.1404
     17 H       -11.2331    0.5000  -15.2898 H       1  UNL1        0.1322
     18 C        -9.0978    2.9984  -12.8726 C.3     1  UNL1       -0.5086
     19 C       -11.4944    2.8355  -13.4870 C.2     1  UNL1       -0.4499
     20 C       -10.3242    2.2159  -13.2746 C.2     1  UNL1        0.1286
     21 C       -10.3140   -3.6556  -13.2491 C.3     1  UNL1       -0.5006
     22 C       -10.1378    0.7125  -13.3991 C.3     1  UNL1       -0.1130
     23 C       -10.1641    0.0450  -12.0085 C.3     1  UNL1       -0.2447
     24 C       -10.1367   -1.4567  -12.0872 C.2     1  UNL1       -0.2526
     25 C       -10.3943   -2.1526  -13.2054 C.2     1  UNL1        0.0873
     26 C       -10.7473   -1.4713  -14.5050 C.3     1  UNL1       -0.2952
@<TRIPOS>BOND
     1    17     1    1
     2    15    26    1
     3    14    26    1
     4    26     1    1
     5    26    25    1
     6     1    16    1
     7     1    22    1
     8     8    21    1
     9    10    22    1
    10     5    19    1
    11     3    18    1
    12     9    21    1
    13    19     6    1
    14    19    20    2
    15    22    20    1
    16    22    23    1
    17    20    18    1
    18    21    25    1
    19    21     7    1
    20    25    24    2
    21    18     2    1
    22    18     4    1
    23    24    23    1
    24    24    13    1
    25    23    12    1
    26    23    11    1
```

Compound 12

```
@<TRIPOS>MOLECULE
compuesto_12.out
 24 24 0 0 0
```

```
SMALL
MULLIKEN_CHARGES

@<TRIPOS>ATOM
     1 C        -8.9637   0.0801  -0.8023 C.ar   1  UNL1      -0.1563
     2 C        -8.4561  -1.1909  -0.5307 C.ar   1  UNL1      -0.2060
     3 C        -9.1386  -2.0666   0.3253 C.ar   1  UNL1       0.0995
     4 C       -10.3511  -1.6502   0.8978 C.ar   1  UNL1      -0.2104
     5 C       -10.8574  -0.3805   0.6248 C.ar   1  UNL1      -0.1399
     6 C       -10.1710   0.4985  -0.2308 C.ar   1  UNL1      -0.0176
     7 C       -10.7202   1.8707  -0.5351 C.3    1  UNL1      -0.0043
     8 C       -12.0843   1.7660  -1.2352 C.3    1  UNL1      -0.4875
     9 C       -10.8027   2.7008   0.7536 C.3    1  UNL1      -0.4886
    10 C        -8.5799  -3.4213   0.6301 C.3    1  UNL1      -0.4980
    11 H        -8.4071   0.7478  -1.4530 H      1  UNL1       0.1439
    12 H        -7.5183  -1.5069  -0.9841 H      1  UNL1       0.1488
    13 H       -10.9051  -2.3187   1.5535 H      1  UNL1       0.1491
    14 H       -11.7945  -0.0678   1.0829 H      1  UNL1       0.1448
    15 H       -10.0251   2.4017  -1.2327 H      1  UNL1       0.1192
    16 H       -12.4586   2.7520  -1.5267 H      1  UNL1       0.1489
    17 H       -12.0088   1.1515  -2.1422 H      1  UNL1       0.1539
    18 H       -12.8400   1.3012  -0.5937 H      1  UNL1       0.1527
    19 H       -11.1255   3.7245   0.5498 H      1  UNL1       0.1486
    20 H        -9.8208   2.7486   1.2482 H      1  UNL1       0.1543
    21 H       -11.5041   2.2758   1.4774 H      1  UNL1       0.1526
    22 H        -7.4874  -3.4451   0.5875 H      1  UNL1       0.1619
    23 H        -8.8711  -3.7746   1.6343 H      1  UNL1       0.1629
    24 H        -8.9619  -4.1573  -0.1033 H      1  UNL1       0.1673
@<TRIPOS>BOND
    1   17    8   1
    2   16    8   1
    3   11    1   1
    4    8   18   1
    5    8    7   1
    6   15    7   1
    7   12    2   1
    8    1    2   ar
    9    1    6   ar
   10    7    6   1
   11    7    9   1
   12    2    3   ar
   13    6    5   ar
   14   24   10   1
   15    3   10   1
   16    3    4   ar
   17   19    9   1
   18   22   10   1
   19    5    4   ar
   20    5   14   1
   21   10   23   1
   22    9   20   1
   23    9   21   1
   24    4   13   1
```


Compound 13


```
@<TRIPOS>MOLECULE
compuesto_13.out
 29 30 0 0 0
SMALL
MULLIKEN_CHARGES

@<TRIPOS>ATOM
     1 C        -7.6969  -0.8923 -11.3083 C.3    1  UNL1      -0.3729
     2 H        -7.8926   2.6883  -7.5181 H      1  UNL1       0.1628
     3 H        -6.7495   1.4234  -7.1334 H      1  UNL1       0.1524
     4 H        -8.4063   0.8486  -7.2900 H      1  UNL1       0.1978
```

```
     5 H        -7.7744    3.3451  -10.0070 H       1  UNL1        0.1613
     6 H        -9.3155    2.5102   -9.7040 H       1  UNL1        0.1698
     7 H        -8.3912    1.9402  -11.0720 H       1  UNL1        0.1691
     8 H        -5.5657    1.5650   -9.9285 H       1  UNL1        0.1308
     9 H        -5.4739   -0.1027   -8.0594 H       1  UNL1        0.1315
    10 H        -4.8000   -0.8674   -9.5204 H       1  UNL1        0.1261
    11 H        -5.6658    0.0530  -11.8252 H       1  UNL1        0.1292
    12 H        -7.1104    0.9529  -11.9563 H       1  UNL1        0.1279
    13 H        -6.2512   -2.3648   -9.8344 H       1  UNL1        0.1409
    14 H        -6.9321   -2.2360   -8.2443 H       1  UNL1        0.1492
    15 H        -8.5695   -0.4549  -11.8393 H       1  UNL1        0.1490
    16 H        -7.4488   -1.8657  -11.7967 H       1  UNL1        0.1469
    17 C        -9.3190   -1.9644   -9.5738 C.3     1  UNL1       -0.5677
    18 C        -7.6605    1.6005   -7.7066 C.3     1  UNL1       -0.5622
    19 C        -8.2849    2.3442  -10.0439 C.3     1  UNL1       -0.5689
    20 C        -7.6124    1.2410   -9.2033 C.3     1  UNL1        0.4385
    21 O        -8.5108    0.0933   -9.2337 O.3     1  UNL1       -0.5757
    22 C        -6.2952    0.7328   -9.8705 C.3     1  UNL1       -0.1715
    23 C        -5.7341   -0.4664   -9.0714 C.3     1  UNL1       -0.2302
    24 C        -6.6198    0.2196  -11.3101 C.3     1  UNL1       -0.2473
    25 C        -6.7380   -1.6423   -9.1576 C.3     1  UNL1       -0.3673
    26 C        -8.0430   -1.1609   -9.8320 C.3     1  UNL1        0.4480
    27 H        -9.7414   -1.3944   -8.6917 H       1  UNL1        0.2025
    28 H       -10.0419   -1.8073  -10.3814 H       1  UNL1        0.1639
    29 H        -9.2520   -3.0682   -9.4309 H       1  UNL1        0.1660
@<TRIPOS>BOND
     1    12    24    1
     2    15     1    1
     3    11    24    1
     4    16     1    1
     5    24     1    1
     6    24    22    1
     7     1    26    1
     8     7    19    1
     9    28    17    1
    10    19     5    1
    11    19     6    1
    12    19    20    1
    13     8    22    1
    14    22    20    1
    15    22    23    1
    16    13    25    1
    17    26    17    1
    18    26    21    1
    19    26    25    1
    20    17    29    1
    21    17    27    1
    22    10    23    1
    23    21    20    1
    24    20    18    1
    25    25    23    1
    26    25    14    1
    27    23     9    1
    28    18     2    1
    29    18     4    1
    30    18     3    1
```

Compound 14

```
@<TRIPOS>MOLECULE
compuesto_14.out
 29 28 0 0 0
SMALL
MULLIKEN_CHARGES

@<TRIPOS>ATOM
     1 C        -7.2468    0.0097   -9.4289 C.3     1  UNL1        0.4030
```

```
     2 H        -7.4671   2.1241   -8.8997 H       1  UNL1        0.1562
     3 H        -8.0350   0.1206  -11.2908 H       1  UNL1        0.3106
     4 H        -7.7147  -1.5968   -8.1067 H       1  UNL1        0.1760
     5 H        -7.4237  -2.1368   -9.7639 H       1  UNL1        0.1729
     6 H        -8.9792  -1.3737   -9.4130 H       1  UNL1        0.1648
     7 H        -2.2371   0.2522   -5.1820 H       1  UNL1        0.1660
     8 H        -3.3942   1.1875   -4.2392 H       1  UNL1        0.1581
     9 H        -3.0001   1.7189   -5.8872 H       1  UNL1        0.1652
    10 H        -3.2964  -0.1791   -7.6869 H       1  UNL1        0.1513
    11 H        -6.1600  -1.2518   -7.3097 H       1  UNL1        0.1316
    12 H        -4.9654  -1.9005   -8.4323 H       1  UNL1        0.1328
    13 H        -5.4193   1.0498   -8.7755 H       1  UNL1        0.1596
    14 H        -5.2143  -0.1971  -10.0119 H       1  UNL1        0.1698
    15 C        -5.5347  -0.0631   -4.9916 C.3     1  UNL1       -0.4999
    16 C        -9.1265   1.0844   -8.1017 C.2     1  UNL1       -0.3176
    17 C        -7.9847   1.1650   -8.7796 C.2     1  UNL1       -0.2012
    18 O        -7.1840   0.2806  -10.8536 O.3     1  UNL1       -0.5947
    19 C        -7.9007  -1.3479   -9.1719 C.3     1  UNL1       -0.5756
    20 C        -3.1751   0.8361   -5.2527 C.3     1  UNL1       -0.5132
    21 C        -4.3272   0.0862   -5.8655 C.2     1  UNL1        0.1443
    22 C        -4.2242  -0.3344   -7.1354 C.2     1  UNL1       -0.2911
    23 C        -5.3105  -0.9555   -7.9576 C.3     1  UNL1       -0.2125
    24 C        -5.7440   0.0270   -9.0619 C.3     1  UNL1       -0.3420
    25 H        -9.5404   1.9339   -7.5774 H       1  UNL1        0.1478
    26 H        -9.7284   0.1889   -8.0024 H       1  UNL1        0.1492
    27 H        -5.2459  -0.6239   -4.0819 H       1  UNL1        0.1634
    28 H        -5.9079   0.9179   -4.6586 H       1  UNL1        0.1621
    29 H        -6.3865  -0.5561   -5.5008 H       1  UNL1        0.1628
@<TRIPOS>BOND
     1     3    18    1
     2    18     1    1
     3    14    24    1
     4     5    19    1
     5     1    19    1
     6     1    24    1
     7     1    17    1
     8     6    19    1
     9    19     4    1
    10    24    13    1
    11    24    23    1
    12     2    17    1
    13    17    16    2
    14    12    23    1
    15    16    26    1
    16    16    25    1
    17    23    11    1
    18    23    22    1
    19    10    22    1
    20    22    21    2
    21     9    20    1
    22    21    20    1
    23    21    15    1
    24    29    15    1
    25    20     7    1
    26    20     8    1
    27    15    28    1
    28    15    27    1
```

Compound 15

```
@<TRIPOS>MOLECULE
compuesto_15.out
 32 31 0 0 0
SMALL
MULLIKEN_CHARGES

@<TRIPOS>ATOM
```

```
   1 C     -9.3721   1.0777  -12.7652 C.3    1  UNL1     -0.4978
   2 H     -5.3158  -0.6419   -4.9619 H      1  UNL1      0.1251
   3 H     -8.9770  -2.0372  -11.1977 H      1  UNL1      0.1515
   4 H     -8.0420  -1.4026  -12.5777 H      1  UNL1      0.1550
   5 H     -9.8162  -1.6259  -12.7178 H      1  UNL1      0.1539
   6 H     -6.0720   1.4942   -6.0649 H      1  UNL1      0.1847
   7 H     -4.8924   1.1926   -7.3903 H      1  UNL1      0.1878
   8 H     -8.8652  -0.1557   -8.7927 H      1  UNL1      0.1317
   9 H     -7.5760  -1.2213   -9.5615 H      1  UNL1      0.1344
  10 H     -8.2424   1.7021  -10.3935 H      1  UNL1      0.1557
  11 H     -7.0917   0.5786  -11.2210 H      1  UNL1      0.1554
  12 H    -10.1447   0.1418  -10.9400 H      1  UNL1      0.1104
  13 H    -10.2463   0.7481  -13.3994 H      1  UNL1      0.1539
  14 H     -8.4785   1.0868  -13.3812 H      1  UNL1      0.1538
  15 H     -9.5887   2.1070  -12.4323 H      1  UNL1      0.1502
  16 C     -3.8662  -1.2286   -6.4998 C.3    1  UNL1     -0.5051
  17 C     -3.6645   0.7772   -4.9372 C.3    1  UNL1     -0.5062
  18 C     -4.6436  -0.1545   -5.7005 C.3    1  UNL1      0.0643
  19 O     -7.1870  -1.0818   -6.9124 O.2    1  UNL1     -0.5243
  20 C     -8.9843  -1.3266  -12.0473 C.3    1  UNL1     -0.5007
  21 C     -5.5583   0.7022   -6.6587 C.3    1  UNL1     -0.4445
  22 C     -6.6155  -0.1154   -7.3469 C.2    1  UNL1      0.6496
  23 O     -6.8965   0.4604   -8.5603 O.3    1  UNL1     -0.4625
  24 C     -7.9047  -0.1735   -9.3605 C.3    1  UNL1     -0.0050
  25 C     -8.0432   0.6337  -10.6629 C.3    1  UNL1     -0.3398
  26 C     -9.2187   0.1180  -11.5584 C.3    1  UNL1      0.0302
  27 H     -4.2202   1.5385   -4.3462 H      1  UNL1      0.1529
  28 H     -3.0521   0.1880   -4.2525 H      1  UNL1      0.1567
  29 H     -3.0028   1.3031   -5.6511 H      1  UNL1      0.1558
  30 H     -4.5618  -1.9267   -7.0151 H      1  UNL1      0.1632
  31 H     -3.2229  -0.7665   -7.2456 H      1  UNL1      0.1520
  32 H     -3.2318  -1.8332   -5.7959 H      1  UNL1      0.1576
@<TRIPOS>BOND
   1   13    1    1
   2   14    1    1
   3    1   15    1
   4    1   26    1
   5    5   20    1
   6    4   20    1
   7   20   26    1
   8   20    3    1
   9   26   12    1
  10   26   25    1
  11   11   25    1
  12   25   10    1
  13   25   24    1
  14    9   24    1
  15   24    8    1
  16   24   23    1
  17   23   22    1
  18    7   21    1
  19   22   19    2
  20   22   21    1
  21   31   16    1
  22   30   16    1
  23   21    6    1
  24   21   18    1
  25   16   32    1
  26   16   18    1
  27   18    2    1
  28   18   17    1
  29   29   17    1
  30   17   27    1
  31   17   28    1
```

Compound 16

```
@<TRIPOS>MOLECULE
compuesto_16.out
 27 28 0 0 0
SMALL
MULLIKEN_CHARGES

@<TRIPOS>ATOM
     1 C        -11.6476   -1.2493  -13.5009 C.3     1  UNL1       -0.2781
     2 C        -10.6733    1.3528  -14.6932 C.3     1  UNL1       -0.5050
     3 C         -8.7035    1.2580  -13.1846 C.3     1  UNL1       -0.5196
     4 C         -9.3012   -1.5844  -14.5444 C.3     1  UNL1       -0.4740
     5 O         -9.0396   -2.1562  -11.6580 O.2     1  UNL1       -0.4839
     6 H        -12.0267   -1.2531  -14.5411 H       1  UNL1        0.1431
     7 H        -11.8327   -2.2467  -13.0708 H       1  UNL1        0.1399
     8 H        -10.9773   -0.5793  -10.3768 H       1  UNL1        0.1774
     9 H         -9.5202    0.4333  -10.6174 H       1  UNL1        0.1772
    10 H        -12.8527   -0.5135  -11.7846 H       1  UNL1        0.1371
    11 H        -12.9748    0.4983  -13.2633 H       1  UNL1        0.1363
    12 H        -11.3066    1.7240  -11.8568 H       1  UNL1        0.1271
    13 H        -10.0008    1.2304  -15.5650 H       1  UNL1        0.1573
    14 H        -11.6592    0.9769  -15.0097 H       1  UNL1        0.1559
    15 H        -10.8127    2.4356  -14.4776 H       1  UNL1        0.1544
    16 H         -8.1271    0.7390  -12.3905 H       1  UNL1        0.1582
    17 H         -8.0918    1.2063  -14.0977 H       1  UNL1        0.1595
    18 H         -8.8055    2.3246  -12.9001 H       1  UNL1        0.1574
    19 H         -8.2271   -1.3434  -14.4307 H       1  UNL1        0.1609
    20 H         -9.3962   -2.6877  -14.4687 H       1  UNL1        0.1639
    21 H         -9.6223   -1.2759  -15.5579 H       1  UNL1        0.1546
    22 C        -10.1001    0.6615  -13.4462 C.3     1  UNL1        0.1604
    23 C        -11.0581    0.7128  -12.1932 C.3     1  UNL1       -0.0927
    24 C        -12.2857   -0.1200  -12.6512 C.3     1  UNL1       -0.2840
    25 C        -10.3119   -0.1398  -11.1380 C.3     1  UNL1       -0.4589
    26 C         -9.7105   -1.2273  -12.0207 C.2     1  UNL1        0.5282
    27 C        -10.1272   -0.9046  -13.4750 C.3     1  UNL1       -0.0526
@<TRIPOS>BOND
     1    13     2    1
     2    21     4    1
     3    14     2    1
     4     2    15    1
     5     2    22    1
     6     4    20    1
     7     4    19    1
     8     4    27    1
     9     6     1    1
    10    17     3    1
    11     1    27    1
    12     1     7    1
    13     1    24    1
    14    27    22    1
    15    27    26    1
    16    22     3    1
    17    22    23    1
    18    11    24    1
    19     3    18    1
    20     3    16    1
    21    24    23    1
    22    24    10    1
    23    23    12    1
    24    23    25    1
    25    26     5    2
    26    26    25    1
    27    25     9    1
    28    25     8    1
```

Compound 17

```
@<TRIPOS>MOLECULE
```

```
compuesto_17.out
 29 29 0 0 0
SMALL
MULLIKEN_CHARGES

@<TRIPOS>ATOM
     1 C        -8.1201   -0.1052   -9.6643 C.2     1  UNL1       0.5636
     2 C        -7.7878   -1.5340  -10.0692 C.3     1  UNL1      -0.4824
     3 C        -6.6057   -2.1067   -9.2613 C.3     1  UNL1       0.0276
     4 C        -6.6888   -1.5568   -7.8243 C.3     1  UNL1      -0.3177
     5 C        -6.4930   -0.0289   -7.7891 C.3     1  UNL1      -0.2381
     6 C        -6.9521    0.6820   -9.0827 C.3     1  UNL1      -0.2801
     7 C        -7.2078    2.2009   -8.8969 C.3     1  UNL1       0.0348
     8 C        -7.4387    2.9021  -10.2539 C.3     1  UNL1      -0.5039
     9 C        -6.0370    2.9077   -8.1806 C.3     1  UNL1      -0.5001
    10 C        -6.6085   -3.6429   -9.2822 C.3     1  UNL1      -0.5048
    11 O        -9.2319    0.3379   -9.8027 O.2     1  UNL1      -0.5003
    12 H        -8.6973   -2.1604   -9.8953 H       1  UNL1       0.1857
    13 H        -7.5602   -1.5571  -11.1619 H       1  UNL1       0.1755
    14 H        -5.6456   -1.7573   -9.7115 H       1  UNL1       0.1137
    15 H        -7.6871   -1.8241   -7.4054 H       1  UNL1       0.1456
    16 H        -5.9235   -2.0417   -7.1758 H       1  UNL1       0.1405
    17 H        -7.0549    0.3731   -6.9106 H       1  UNL1       0.1401
    18 H        -5.4164    0.2023   -7.6232 H       1  UNL1       0.1358
    19 H        -6.1363    0.5873   -9.8420 H       1  UNL1       0.1575
    20 H        -8.1214    2.3330   -8.2686 H       1  UNL1       0.1255
    21 H        -8.2976    2.4617  -10.8172 H       1  UNL1       0.1661
    22 H        -7.6664    3.9807  -10.1133 H       1  UNL1       0.1526
    23 H        -6.5394    2.8303  -10.9046 H       1  UNL1       0.1459
    24 H        -5.9017    2.5398   -7.1395 H       1  UNL1       0.1475
    25 H        -5.0825    2.7482   -8.7257 H       1  UNL1       0.1501
    26 H        -6.2158    4.0074   -8.1150 H       1  UNL1       0.1540
    27 H        -6.5640   -4.0284  -10.3274 H       1  UNL1       0.1548
    28 H        -5.7264   -4.0495   -8.7421 H       1  UNL1       0.1537
    29 H        -7.5231   -4.0475   -8.8020 H       1  UNL1       0.1566
@<TRIPOS>BOND
    1   13    2    1
    2   23    8    1
    3   21    8    1
    4   27   10    1
    5    8   22    1
    6    8    7    1
    7    2   12    1
    8    2    1    1
    9    2    3    1
   10   19    6    1
   11   11    1    2
   12   14    3    1
   13    1    6    1
   14   10    3    1
   15   10   29    1
   16   10   28    1
   17    3    4    1
   18    6    7    1
   19    6    5    1
   20    7   20    1
   21    7    9    1
   22   25    9    1
   23    9   26    1
   24    9   24    1
   25    4    5    1
   26    4   15    1
   27    4   16    1
   28    5   18    1
   29    5   17    1
```

Compound 18

```
@<TRIPOS>MOLECULE
compuesto_18.out
 29 29 0 0 0
SMALL
MULLIKEN_CHARGES

@<TRIPOS>ATOM
      1 H          -7.3225   -4.0277  -11.2962 H       1  UNL1        0.1550
      2 H          -6.4801   -4.0487   -9.7057 H       1  UNL1        0.1536
      3 H          -8.2725   -4.0529   -9.7651 H       1  UNL1        0.1563
      4 C          -8.8742   -0.1048  -10.6267 C.2     1  UNL1        0.5634
      5 C          -8.5422   -1.5338  -11.0333 C.3     1  UNL1       -0.4821
      6 C          -7.3597   -2.1063  -10.2261 C.3     1  UNL1        0.0276
      7 C          -7.4443   -1.5581   -8.7877 C.3     1  UNL1       -0.3177
      8 C          -7.2465   -0.0293   -8.7536 C.3     1  UNL1       -0.2381
      9 C          -7.7043    0.6818  -10.0483 C.3     1  UNL1       -0.2797
     10 C          -7.9632    2.2014   -9.8629 C.3     1  UNL1        0.0355
     11 C          -8.1919    2.8987  -11.2203 C.3     1  UNL1       -0.5043
     12 C          -6.7917    2.9068   -9.1457 C.3     1  UNL1       -0.5007
     13 C          -7.3620   -3.6433  -10.2491 C.3     1  UNL1       -0.5049
     14 O          -9.9899    0.3369  -10.7685 O.2     1  UNL1       -0.5016
     15 H          -9.4538   -2.1591  -10.8667 H       1  UNL1        0.1857
     16 H          -8.3117   -1.5598  -12.1248 H       1  UNL1        0.1755
     17 H          -6.3959   -1.7569  -10.6763 H       1  UNL1        0.1140
     18 H          -8.4429   -1.8269   -8.3727 H       1  UNL1        0.1456
     19 H          -6.6783   -2.0417   -8.1352 H       1  UNL1        0.1406
     20 H          -7.8125    0.3734   -7.8817 H       1  UNL1        0.1398
     21 H          -6.1648    0.2005   -8.5848 H       1  UNL1        0.1364
     22 H          -6.8894    0.5915  -10.8069 H       1  UNL1        0.1577
     23 H          -8.8761    2.3284   -9.2354 H       1  UNL1        0.1254
     24 H          -9.0525    2.4679  -11.7843 H       1  UNL1        0.1665
     25 H          -8.4146    3.9812  -11.0790 H       1  UNL1        0.1528
     26 H          -7.2914    2.8301  -11.8647 H       1  UNL1        0.1460
     27 H          -6.6667    2.5442   -8.1055 H       1  UNL1        0.1477
     28 H          -5.8374    2.7491   -9.6895 H       1  UNL1        0.1505
     29 H          -6.9642    4.0033   -9.0787 H       1  UNL1        0.1535
@<TRIPOS>BOND
     1    16     5    1
     2    26    11    1
     3    24    11    1
     4     1    13    1
     5    11    25    1
     6    11    10    1
     7     5    15    1
     8     5     4    1
     9     5     6    1
    10    22     9    1
    11    14     4    2
    12    17     6    1
    13     4     9    1
    14    13     6    1
    15    13     3    1
    16    13     2    1
    17     6     7    1
    18     9    10    1
    19     9     8    1
    20    10    23    1
    21    10    12    1
    22    28    12    1
    23    12    29    1
    24    12    27    1
    25     7     8    1
    26     7    18    1
    27     7    19    1
```

```
28   8  21   1
29   8  20   1
```

Compound 19

```
@<TRIPOS>MOLECULE
compuesto_19.out
 29 30 0 0 0
SMALL
MULLIKEN_CHARGES

@<TRIPOS>ATOM
    1 C        7.5611   -0.7301   11.6223 C.3    1  UNL1     -0.2821
    2 C        5.8507    0.8587   10.8704 C.3    1  UNL1      0.1322
    3 O        6.3912   -2.6868    9.9712 O.3    1  UNL1     -0.5619
    4 C        5.1452   -1.1316   12.4338 C.3    1  UNL1     -0.5085
    5 C        6.1899    1.8937   11.9540 C.3    1  UNL1     -0.5069
    6 C        4.4634    1.2279   10.3171 C.3    1  UNL1     -0.5128
    7 H        4.7021   -1.5289    9.7734 H      1  UNL1      0.0965
    8 H        7.7668   -0.3667   12.6408 H      1  UNL1      0.1284
    9 H        7.9383   -1.7583   11.5448 H      1  UNL1      0.1545
   10 H        7.2740   -0.9677    8.3732 H      1  UNL1      0.1564
   11 H        5.7225   -0.1527    8.1507 H      1  UNL1      0.1367
   12 H        8.9000   -0.3360    9.9122 H      1  UNL1      0.1348
   13 H        8.7427    1.0296   11.0023 H      1  UNL1      0.1253
   14 H        7.1447    1.6171    9.1886 H      1  UNL1      0.1191
   15 H        6.0819   -3.1822    9.1875 H      1  UNL1      0.3046
   16 H        4.0853   -0.9662   12.2328 H      1  UNL1      0.1526
   17 H        5.3282   -2.1913   12.6454 H      1  UNL1      0.1674
   18 H        5.3243   -0.5690   13.3451 H      1  UNL1      0.1541
   19 H        6.2557    2.9079   11.5526 H      1  UNL1      0.1515
   20 H        5.4327    1.8888   12.7430 H      1  UNL1      0.1521
   21 H        7.1507    1.6625   12.4212 H      1  UNL1      0.1587
   22 H        3.7298    1.2532   11.1366 H      1  UNL1      0.1543
   23 H        4.5240    2.2347    9.8840 H      1  UNL1      0.1541
   24 H        4.0815    0.5423    9.5390 H      1  UNL1      0.1517
   25 C        6.9614    0.7139    9.7626 C.3    1  UNL1     -0.0959
   26 C        8.1936    0.2002   10.5559 C.3    1  UNL1     -0.2748
   27 C        6.4541   -0.4692    8.9029 C.3    1  UNL1     -0.3722
   28 C        5.7862   -1.3877    9.9527 C.3    1  UNL1      0.1460
   29 C        6.0322   -0.6533   11.3090 C.3    1  UNL1      0.0845
@<TRIPOS>BOND
    1   11   27    1
    2   10   27    1
    3   27   25    1
    4   27   28    1
    5   15    3    1
    6   14   25    1
    7   24    6    1
    8   25   26    1
    9   25    2    1
   10    7   28    1
   11   23    6    1
   12   12   26    1
   13   28    3    1
   14   28   29    1
   15    6    2    1
   16    6   22    1
   17   26   13    1
   18   26    1    1
   19    2   29    1
   20    2    5    1
   21   29    1    1
   22   29    4    1
   23    9    1    1
   24   19    5    1
   25    1    8    1
```

```
   26    5   21   1
   27    5   20   1
   28   16    4   1
   29    4   17   1
   30    4   18   1
```

Compound 20

```
@<TRIPOS>MOLECULE
compuesto_20.out
 31 31 0 0 0
SMALL
MULLIKEN_CHARGES

@<TRIPOS>ATOM
      1 C          5.8656    1.5293   10.9886 C.3     1  UNL1       -0.3047
      2 C          5.3891   -2.2037    9.7823 C.3     1  UNL1        0.0171
      3 C          6.3248   -2.8841   10.8058 C.3     1  UNL1       -0.5009
      4 C          5.5410   -2.9695    8.4530 C.3     1  UNL1       -0.4928
      5 H          4.8483    1.8105   11.3364 H       1  UNL1        0.1363
      6 H          6.5726    1.9799   11.7145 H       1  UNL1        0.1345
      7 H          5.3695   -0.4126   11.7898 H       1  UNL1        0.1338
      8 H          7.0589   -0.2569   11.2486 H       1  UNL1        0.1348
      9 H          6.5637   -0.5936    8.9588 H       1  UNL1        0.1434
     10 H          3.6935    0.2144    9.7524 H       1  UNL1        0.0980
     11 H          5.4194    1.5209    7.5990 H       1  UNL1        0.1586
     12 H          4.1302    2.2398    8.6176 H       1  UNL1        0.1428
     13 H          7.0948    1.7629    9.2171 H       1  UNL1        0.1169
     14 H          6.2955    4.0322    8.5846 H       1  UNL1        0.1523
     15 H          5.1485    4.0549    9.9709 H       1  UNL1        0.1536
     16 H          6.9016    4.0427   10.2287 H       1  UNL1        0.1507
     17 H          3.3091    0.1215    7.4773 H       1  UNL1        0.3087
     18 H          4.3406   -2.3193   10.1487 H       1  UNL1        0.1133
     19 H          6.1662   -3.9766   10.8421 H       1  UNL1        0.1490
     20 H          7.3866   -2.7304   10.5392 H       1  UNL1        0.1515
     21 H          6.1250   -2.5173   11.8298 H       1  UNL1        0.1451
     22 H          5.3948   -4.0542    8.6049 H       1  UNL1        0.1433
     23 H          4.8308   -2.6266    7.6794 H       1  UNL1        0.1711
     24 H          6.5568   -2.8438    8.0364 H       1  UNL1        0.1455
     25 O          3.9981   -0.4578    7.8537 O.3     1  UNL1       -0.5774
     26 C          6.1041    3.6457    9.5922 C.3     1  UNL1       -0.5069
     27 C          6.0916    2.1115    9.5850 C.3     1  UNL1        0.0164
     28 C          5.0097    1.5669    8.6319 C.3     1  UNL1       -0.4023
     29 C          4.5358    0.1546    9.0231 C.3     1  UNL1        0.1887
     30 C          5.6766   -0.6800    9.6311 C.3     1  UNL1       -0.1515
     31 C          6.0199   -0.0045   10.9858 C.3     1  UNL1       -0.2689
@<TRIPOS>BOND
    1   17   25   1
    2   11   28   1
    3   23    4   1
    4   25   29   1
    5   24    4   1
    6    4   22   1
    7    4    2   1
    8   14   26   1
    9   12   28   1
   10   28   29   1
   11   28   27   1
   12    9   30   1
   13   29   30   1
   14   29   10   1
   15   13   27   1
   16   27   26   1
   17   27    1   1
   18   26   15   1
   19   26   16   1
   20   30    2   1
```

```
  21   30   31   1
  22    2   18   1
  23    2    3   1
  24   20    3   1
  25    3   19   1
  26    3   21   1
  27   31    1   1
  28   31    8   1
  29   31    7   1
  30    1    5   1
  31    1    6   1
```

Compound 21

```
@<TRIPOS>MOLECULE
compuesto_21.out
 31 31 0 0 0
SMALL
MULLIKEN_CHARGES

@<TRIPOS>ATOM
      1 C          6.8597    1.5159   12.0206 C.3     1  UNL1      -0.2974
      2 H          6.7320    2.0278   13.0008 H       1  UNL1       0.1314
      3 H          7.9372    1.6018   11.7551 H       1  UNL1       0.1325
      4 H          5.4289   -0.0519   12.5293 H       1  UNL1       0.1475
      5 H          7.1288   -0.4099   12.9370 H       1  UNL1       0.1262
      6 H          7.6901   -0.6969   10.5272 H       1  UNL1       0.1298
      7 H          6.0111   -0.4932    8.7347 H       1  UNL1       0.0888
      8 H          7.2284    1.5610    9.2659 H       1  UNL1       0.1496
      9 H          5.5343    1.9472    8.8333 H       1  UNL1       0.1395
     10 H          4.9393    2.1734   11.2538 H       1  UNL1       0.1247
     11 H          5.7898    4.2013   10.0433 H       1  UNL1       0.1488
     12 H          6.2752    4.2364   11.7597 H       1  UNL1       0.1518
     13 H          7.4702    3.8039   10.4914 H       1  UNL1       0.1508
     14 H          5.1782   -2.2949   11.3463 H       1  UNL1       0.1294
     15 H          7.0032   -2.5940   13.0342 H       1  UNL1       0.1482
     16 H          6.8940   -4.0506   12.0374 H       1  UNL1       0.1496
     17 H          8.1965   -2.8638   11.7281 H       1  UNL1       0.1486
     18 H          5.5455   -2.6538    8.9213 H       1  UNL1       0.1512
     19 H          7.3061   -2.8972    9.1745 H       1  UNL1       0.1466
     20 H          6.1077   -4.0818    9.7612 H       1  UNL1       0.1496
     21 H          3.9427    0.2638    9.2455 H       1  UNL1       0.3053
     22 O          4.4305   -0.1443    9.9729 O.3     1  UNL1      -0.5661
     23 C          6.3058   -2.9994    9.6399 C.3     1  UNL1      -0.4984
     24 C          7.1285   -2.9665   11.9991 C.3     1  UNL1      -0.4984
     25 C          6.2327   -2.2440   10.9783 C.3     1  UNL1       0.0092
     26 C          6.4121    3.6937   10.8029 C.3     1  UNL1      -0.5087
     27 C          6.0135    2.2169   10.9457 C.3     1  UNL1       0.0180
     28 C          6.1741    1.4711    9.6109 C.3     1  UNL1      -0.3955
     29 C          5.8214   -0.0157    9.7252 C.3     1  UNL1       0.1857
     30 C          6.6135   -0.7374   10.8301 C.3     1  UNL1      -0.1407
     31 C          6.4747    0.0346   12.1585 C.3     1  UNL1      -0.2574
@<TRIPOS>BOND
   1    7   29   1
   2    9   28   1
   3   18   23   1
   4   19   23   1
   5   21   22   1
   6    8   28   1
   7   28   29   1
   8   28   27   1
   9   23   20   1
  10   23   25   1
  11   29   22   1
  12   29   30   1
  13   11   26   1
  14   13   26   1
```

```
  15    6   30    1
  16   26   27    1
  17   26   12    1
  18   30   25    1
  19   30   31    1
  20   27   10    1
  21   27    1    1
  22   25   14    1
  23   25   24    1
  24   17   24    1
  25    3    1    1
  26   24   16    1
  27   24   15    1
  28    1   31    1
  29    1    2    1
  30   31    4    1
  31   31    5    1
```

Compound 22

```
@<TRIPOS>MOLECULE
compuesto_22.out
 25 25 0 0 0
SMALL
MULLIKEN_CHARGES

@<TRIPOS>ATOM
      1 C          7.2692    1.5337   11.9205 C.2     1  UNL1        0.5500
      2 H          5.4840    4.1336   10.5458 H       1  UNL1        0.1466
      3 H          6.5522   -3.8850    9.9152 H       1  UNL1        0.1626
      4 H          6.4118   -2.3069    8.9675 H       1  UNL1        0.1609
      5 H          5.3898   -2.7568   13.0019 H       1  UNL1        0.1665
      6 H          7.1568   -2.6350   13.0160 H       1  UNL1        0.1683
      7 H          6.3459   -3.9669   12.2316 H       1  UNL1        0.1624
      8 H          4.9656    2.1156    9.5081 H       1  UNL1        0.1403
      9 H          4.7361   -0.3211    9.5889 H       1  UNL1        0.1546
     10 H          6.4386   -0.0588    9.0847 H       1  UNL1        0.1625
     11 H          5.2895   -0.4683   11.9482 H       1  UNL1        0.1391
     12 H          7.6664   -0.4272   12.7442 H       1  UNL1        0.1804
     13 H          8.2337   -0.1436   11.0474 H       1  UNL1        0.1821
     14 C          6.2178    3.7200   11.2284 C.3     1  UNL1       -0.4358
     15 O          7.9317    2.1277   12.7209 O.2     1  UNL1       -0.4907
     16 C          6.4182   -2.8015    9.9358 C.2     1  UNL1       -0.4384
     17 C          6.2935   -2.9056   12.3955 C.3     1  UNL1       -0.5122
     18 C          6.2919   -2.1580   11.0930 C.2     1  UNL1        0.1247
     19 C          6.3435    2.2386   11.0631 C.2     1  UNL1       -0.1634
     20 C          5.6294    1.5611   10.1498 C.2     1  UNL1       -0.0789
     21 C          5.7137    0.0843    9.9115 C.3     1  UNL1       -0.3068
     22 H          5.9054    3.9787   12.2497 H       1  UNL1        0.1702
     23 H          7.1636    4.2503   11.0077 H       1  UNL1        0.1700
     24 C          6.1242   -0.6513   11.2148 C.3     1  UNL1       -0.0672
     25 C          7.3979    0.0227   11.7621 C.3     1  UNL1       -0.4477
@<TRIPOS>BOND
   1    4   16    1
   2   10   21    1
   3    8   20    1
   4    9   21    1
   5   21   20    1
   6   21   24    1
   7    3   16    1
   8   16   18    2
   9   20   19    2
  10    2   14    1
  11   23   14    1
  12   13   25    1
  13   19   14    1
  14   19    1    1
```

```
15 18 24 1
16 18 17 1
17 24 25 1
18 24 11 1
19 14 22 1
20 25 1 1
21 25 12 1
22 1 15 2
23 7 17 1
24 17 5 1
25 17 6 1
```

Compound 23

```
@<TRIPOS>MOLECULE
compuesto_23.out
 36 36 0 0 0
SMALL
MULLIKEN_CHARGES

@<TRIPOS>ATOM
 1 C 6.8000 1.5734 11.1391 C.3 1 UNL1 -0.3035
 2 H 4.2327 -2.6376 8.6153 H 1 UNL1 0.1582
 3 H 7.6187 -2.7611 10.5307 H 1 UNL1 0.1540
 4 H 6.4171 -3.8959 11.1945 H 1 UNL1 0.1502
 5 H 6.8055 -2.4601 12.1054 H 1 UNL1 0.1464
 6 H 4.6347 -2.0521 10.9574 H 1 UNL1 0.1091
 7 H 6.6996 4.0299 9.7364 H 1 UNL1 0.1575
 8 H 6.1766 4.2827 11.4367 H 1 UNL1 0.1499
 9 H 5.0022 4.4057 10.1219 H 1 UNL1 0.1541
 10 H 4.7258 2.2519 11.3461 H 1 UNL1 0.1121
 11 H 4.2357 2.3229 8.9428 H 1 UNL1 0.1681
 12 H 5.9469 1.8863 8.5529 H 1 UNL1 0.1662
 13 H 3.9086 0.1503 10.0427 H 1 UNL1 0.1182
 14 H 6.7833 -0.5990 9.2848 H 1 UNL1 0.1417
 15 H 7.3569 -0.3829 11.6690 H 1 UNL1 0.1370
 16 H 5.6723 -0.0071 12.1103 H 1 UNL1 0.1346
 17 H 7.6275 1.6997 10.4209 H 1 UNL1 0.1430
 18 H 7.1357 2.0013 12.0924 H 1 UNL1 0.1342
 19 O 4.3048 1.3898 6.6615 O.2 1 UNL1 -0.5275
 20 C 3.8801 -0.8628 5.9555 C.3 1 UNL1 -0.5890
 21 C 4.2140 0.2191 6.9419 C.2 1 UNL1 0.6934
 22 O 4.4197 -0.3672 8.1529 O.3 1 UNL1 -0.4954
 23 C 5.2039 -2.9179 9.0520 C.3 1 UNL1 -0.4865
 24 C 6.6660 -2.8196 11.0893 C.3 1 UNL1 -0.4994
 25 C 5.5436 -2.0773 10.3217 C.3 1 UNL1 0.0117
 26 C 5.8913 3.8603 10.4611 C.3 1 UNL1 -0.5002
 27 C 5.9470 -0.5929 10.0053 C.3 1 UNL1 -0.1620
 28 C 6.4312 0.1108 11.3164 C.3 1 UNL1 -0.2648
 29 H 4.6917 -1.5780 5.8476 H 1 UNL1 0.1968
 30 H 2.9552 -1.3779 6.2495 H 1 UNL1 0.1964
 31 H 3.6981 -0.4251 4.9522 H 1 UNL1 0.1987
 32 H 5.9859 -2.7874 8.2890 H 1 UNL1 0.1500
 33 H 5.1402 -4.0013 9.3114 H 1 UNL1 0.1438
 34 C 5.5784 2.3620 10.6270 C.3 1 UNL1 0.0016
 35 C 5.1411 1.7510 9.2673 C.3 1 UNL1 -0.3812
 36 C 4.8102 0.2602 9.3810 C.3 1 UNL1 0.1828
@<TRIPOS>BOND
 1 31 20 1
 2 29 20 1
 3 20 30 1
 4 20 21 1
 5 19 21 2
 6 21 22 1
 7 22 36 1
 8 32 23 1
 9 12 35 1
```

```
  10    2   23   1
  11   11   35   1
  12   23   33   1
  13   23   25   1
  14   35   36   1
  15   35   34   1
  16   14   27   1
  17   36   27   1
  18   36   13   1
  19    7   26   1
  20   27   25   1
  21   27   28   1
  22    9   26   1
  23   25    6   1
  24   25   24   1
  25   17    1   1
  26   26   34   1
  27   26    8   1
  28    3   24   1
  29   34    1   1
  30   34   10   1
  31   24    4   1
  32   24    5   1
  33    1   28   1
  34    1   18   1
  35   28   15   1
  36   28   16   1
```

Compound 24

```
@<TRIPOS>MOLECULE
compuesto_24.out
 39 41 0 0 0
SMALL
MULLIKEN_CHARGES

@<TRIPOS>ATOM
     1 C        6.3587    1.9152   13.2677 C.3     1  UNL1      -0.2570
     2 C        6.8329    1.2934   11.9516 C.3     1  UNL1       0.0103
     3 C        6.4846   -0.1897   11.7399 C.3     1  UNL1      -0.1645
     4 C        7.8453    0.1805   12.3321 C.3     1  UNL1      -0.2482
     5 C        7.8574    0.1452   13.8320 C.2     1  UNL1       0.0993
     6 H        3.9134   -2.0654   10.1081 H       1  UNL1       0.1468
     7 H        4.5968   -3.6625   10.4414 H       1  UNL1       0.1478
     8 H        8.5520   -0.8074   15.6348 H       1  UNL1       0.1601
     9 H        8.3660   -1.8846   14.2402 H       1  UNL1       0.1660
    10 H        9.7343   -0.7651   14.3189 H       1  UNL1       0.1646
    11 H        7.3062    4.1078    9.9164 H       1  UNL1       0.1482
    12 H        6.2935    4.0757   11.3586 H       1  UNL1       0.1516
    13 H        8.0319    3.7886   11.4953 H       1  UNL1       0.1555
    14 C        7.0206    1.0714   14.3408 C.2     1  UNL1      -0.2575
    15 H        4.6285   -2.4078   11.6838 H       1  UNL1       0.1531
    16 H        7.2379   -2.8319   11.8155 H       1  UNL1       0.1579
    17 H        8.2061   -2.6531   10.3572 H       1  UNL1       0.1463
    18 H        7.0974   -4.0203   10.5143 H       1  UNL1       0.1456
    19 H        6.1157   -2.3111    8.9814 H       1  UNL1       0.1084
    20 H        7.8300    1.7167   10.0970 H       1  UNL1       0.1239
    21 H        4.8098    2.3029   10.3088 H       1  UNL1       0.1349
    22 H        5.7835    2.3180    8.8381 H       1  UNL1       0.1272
    23 H        4.3535    0.1038   10.0026 H       1  UNL1       0.1358
    24 H        5.3216    0.0963    8.5278 H       1  UNL1       0.1256
    25 H        7.3825   -0.4339    9.7910 H       1  UNL1       0.1259
    26 H        5.2569    1.8954   13.3473 H       1  UNL1       0.1440
    27 H        6.6707    2.9710   13.3587 H       1  UNL1       0.1461
    28 H        8.7661   -0.0160   11.7994 H       1  UNL1       0.1562
    29 H        6.7980    1.2557   15.3736 H       1  UNL1       0.1478
    30 C        7.1498    3.5973   10.8725 C.3     1  UNL1      -0.4917
```

```
   31 C        8.6642  -0.8700  14.5456 C.3    1  UNL1      -0.4906
   32 C        4.7348  -2.5864  10.6088 C.3    1  UNL1      -0.4916
   33 C        7.2204  -2.9539  10.7258 C.3    1  UNL1      -0.4947
   34 C        6.0972  -2.1233  10.0853 C.3    1  UNL1      -0.0024
   35 H        5.7566  -0.6722  12.3977 H      1  UNL1       0.1548
   36 C        6.9411   2.0957  10.6668 C.3    1  UNL1      -0.0309
   37 C        5.6752   1.8196   9.8175 C.3    1  UNL1      -0.2822
   38 C        5.3644   0.3207   9.6069 C.3    1  UNL1      -0.2742
   39 C        6.3906  -0.6131  10.2865 C.3    1  UNL1      -0.0985
@<TRIPOS>BOND
   1   24   38   1
   2   22   37   1
   3   19   34   1
   4   38   37   1
   5   38   23   1
   6   38   39   1
   7   25   39   1
   8   37   21   1
   9   37   36   1
  10   11   30   1
  11   34   39   1
  12   34   32   1
  13   34   33   1
  14   20   36   1
  15    6   32   1
  16   39    3   1
  17   17   33   1
  18    7   32   1
  19   18   33   1
  20   32   15   1
  21   36   30   1
  22   36    2   1
  23   33   16   1
  24   30   12   1
  25   30   13   1
  26    3    2   1
  27    3    4   1
  28    3   35   1
  29   28    4   1
  30    2    4   1
  31    2    1   1
  32    4    5   1
  33    1   26   1
  34    1   27   1
  35    1   14   1
  36    5   14   2
  37    5   31   1
  38    9   31   1
  39   10   31   1
  40   14   29   1
  41   31    8   1
```

Compound 25

```
@<TRIPOS>MOLECULE
compuesto_25.out
 39 42 0 0 0
SMALL
MULLIKEN_CHARGES

@<TRIPOS>ATOM
    1 H        3.1536   0.0169  -16.6099 H     1  UNL1      0.1522
    2 H        4.3486   1.2843  -16.8872 H     1  UNL1      0.1466
    3 H        5.2422  -2.3682  -17.0259 H     1  UNL1      0.1458
    4 H        5.6828  -2.5405  -15.3207 H     1  UNL1      0.1478
    5 H        3.9855  -2.3354  -15.7882 H     1  UNL1      0.1569
    6 C        3.6722  -1.7111  -13.0240 C.3   1  UNL1     -0.2306
```

```
     7 H          5.8202   -0.5555  -13.8503 H       1  UNL1        0.1323
     8 H          6.2535    1.7362  -14.8159 H       1  UNL1        0.1279
     9 H          4.5129    2.0599  -14.8507 H       1  UNL1        0.1366
    10 H          6.1423    1.3660  -12.3136 H       1  UNL1        0.1372
    11 H          5.6456    2.9976  -12.7877 H       1  UNL1        0.1279
    12 H          1.8544    0.2108  -10.8462 H       1  UNL1        0.1424
    13 H          3.2462    2.3837  -12.6331 H       1  UNL1        0.1257
    14 H          2.8224   -0.1747  -14.4354 H       1  UNL1        0.1308
    15 H          1.2915    0.0336  -12.5190 H       1  UNL1        0.1394
    16 H          2.2323   -2.4177  -11.3449 H       1  UNL1        0.1501
    17 H          3.9373   -2.6436  -13.4697 H       1  UNL1        0.1543
    18 C          4.0858    2.1142  -10.6620 C.3     1  UNL1       -0.4854
    19 C          5.0119   -2.0197  -16.0128 C.3     1  UNL1       -0.4968
    20 C          4.2008    0.1994  -16.8758 C.3     1  UNL1       -0.4928
    21 C          5.1616   -0.4966  -15.9042 C.3     1  UNL1       -0.0034
    22 C          5.2565   -1.3168  -10.8107 C.3     1  UNL1       -0.4585
    23 C          4.1371   -1.0463  -11.7216 C.3     1  UNL1        0.0158
    24 C          5.0205   -0.0241  -14.4315 C.3     1  UNL1       -0.1144
    25 C          5.2983    1.4895  -14.3152 C.3     1  UNL1       -0.2779
    26 C          5.3566    1.9328  -12.8478 C.3     1  UNL1       -0.2790
    27 C          4.0040    1.7200  -12.1384 C.3     1  UNL1       -0.0518
    28 C          3.6759   -0.3539  -13.7671 C.3     1  UNL1       -0.1040
    29 C          3.5410    0.3042  -12.3307 C.3     1  UNL1        0.0429
    30 C          2.1243   -0.1570  -11.8411 C.3     1  UNL1       -0.3090
    31 C          2.7016   -1.5829  -11.8155 C.3     1  UNL1       -0.1914
    32 H          6.2050   -0.9237  -11.2048 H       1  UNL1        0.1603
    33 H          5.0888   -0.8641   -9.8226 H       1  UNL1        0.1596
    34 H          5.3937   -2.3987  -10.6534 H       1  UNL1        0.1574
    35 H          6.2091   -0.2387  -16.2171 H       1  UNL1        0.1079
    36 H          4.3436   -0.1631  -17.9009 H       1  UNL1        0.1470
    37 H          3.1082    2.0285  -10.1737 H       1  UNL1        0.1499
    38 H          4.7808    1.4663  -10.1170 H       1  UNL1        0.1539
    39 H          4.4237    3.1487  -10.5434 H       1  UNL1        0.1464
@<TRIPOS>BOND
     1    36    20    1
     2     3    19    1
     3     2    20    1
     4    20     1    1
     5    20    21    1
     6    35    21    1
     7    19    21    1
     8    19     5    1
     9    19     4    1
    10    21    24    1
    11     9    25    1
    12     8    25    1
    13    14    28    1
    14    24    25    1
    15    24     7    1
    16    24    28    1
    17    25    26    1
    18    28     6    1
    19    28    29    1
    20    17     6    1
    21     6    31    1
    22     6    23    1
    23    26    11    1
    24    26    10    1
    25    26    27    1
    26    13    27    1
    27    15    30    1
    28    29    27    1
    29    29    30    1
    30    29    23    1
    31    27    18    1
    32    30    31    1
```

```
   33   30   12    1
   34   31   23    1
   35   31   16    1
   36   23   22    1
   37   32   22    1
   38   22   34    1
   39   22   33    1
   40   18   39    1
   41   18   37    1
   42   18   38    1
```

Compound 26

```
@<TRIPOS>MOLECULE
compuesto_26.out
 33 35 0 0 0
SMALL
MULLIKEN_CHARGES

@<TRIPOS>ATOM
      1 C          4.8432    0.8433  -16.5720 C.2     1  UNL1       -0.2534
      2 C          3.7283   -0.1352  -16.2996 C.3     1  UNL1       -0.2527
      3 C          3.7060   -0.5343  -14.8089 C.3     1  UNL1       -0.1393
      4 C          5.1574   -0.8901  -14.3339 C.3     1  UNL1       -0.1270
      5 C          5.3597    0.6602  -14.1828 C.3     1  UNL1       -0.2046
      6 C          5.6272    1.2430  -15.5519 C.2     1  UNL1        0.0845
      7 C          5.2112   -1.6027  -12.9680 C.3     1  UNL1       -0.0374
      8 C          4.0018   -1.1898  -12.0992 C.3     1  UNL1       -0.2794
      9 C          3.6206    0.2778  -12.3579 C.3     1  UNL1       -0.3136
     10 C          3.8247    0.6955  -13.8368 C.3     1  UNL1        0.1579
     11 C          6.7442    2.2225  -15.6917 C.3     1  UNL1       -0.4979
     12 C          3.0686    1.9656  -14.1575 C.3     1  UNL1       -0.5204
     13 C          5.2752   -3.1233  -13.1369 C.3     1  UNL1       -0.4960
     14 H          2.9208   -1.2579  -14.5761 H       1  UNL1        0.1273
     15 H          6.0466    0.9924  -13.4003 H       1  UNL1        0.1361
     16 H          5.8087   -1.3593  -15.0795 H       1  UNL1        0.1331
     17 H          4.9714    1.2064  -17.5871 H       1  UNL1        0.1430
     18 H          2.7538    0.3076  -16.5874 H       1  UNL1        0.1378
     19 H          3.8658   -1.0459  -16.9175 H       1  UNL1        0.1366
     20 H          6.1468   -1.2706  -12.4511 H       1  UNL1        0.1172
     21 H          3.1343   -1.8340  -12.3467 H       1  UNL1        0.1345
     22 H          4.2091   -1.3596  -11.0247 H       1  UNL1        0.1277
     23 H          2.5650    0.4324  -12.0679 H       1  UNL1        0.1357
     24 H          4.2314    0.9448  -11.7173 H       1  UNL1        0.1373
     25 H          6.5749    3.1025  -15.0536 H       1  UNL1        0.1628
     26 H          7.7025    1.7677  -15.4010 H       1  UNL1        0.1634
     27 H          6.8358    2.5921  -16.7344 H       1  UNL1        0.1589
     28 H          1.9863    1.8170  -14.0662 H       1  UNL1        0.1530
     29 H          3.3655    2.7736  -13.4736 H       1  UNL1        0.1533
     30 H          3.2603    2.3310  -15.1788 H       1  UNL1        0.1696
     31 H          5.2918   -3.6313  -12.1616 H       1  UNL1        0.1485
     32 H          4.4024   -3.4974  -13.6982 H       1  UNL1        0.1535
     33 H          6.1776   -3.4214  -13.6893 H       1  UNL1        0.1500
@<TRIPOS>BOND
    1   17    1    1
    2   19    2    1
    3   27   11    1
    4   18    2    1
    5    1    2    1
    6    1    6    2
    7    2    3    1
    8   11    6    1
    9   11   26    1
   10   11   25    1
   11    6    5    1
   12   30   12    1
   13   16    4    1
```

```
 14   3  14   1
 15   3   4   1
 16   3  10   1
 17   4   5   1
 18   4   7   1
 19   5  10   1
 20   5  15   1
 21  12  28   1
 22  12  10   1
 23  12  29   1
 24  10   9   1
 25  32  13   1
 26  33  13   1
 27  13   7   1
 28  13  31   1
 29   7  20   1
 30   7   8   1
 31   9   8   1
 32   9  23   1
 33   9  24   1
 34  21   8   1
 35   8  22   1
```

Compound 27

```
@<TRIPOS>MOLECULE
compuesto_27.out
 39 41 0 0 0
SMALL
MULLIKEN_CHARGES

@<TRIPOS>ATOM
   1 C    2.2567  -0.0098   -9.9242 C.3   1  UNL1   -0.2972
   2 C    3.7951  -0.0607  -10.1895 C.3   1  UNL1    0.1190
   3 C    3.9897  -0.8787  -11.4462 C.2   1  UNL1   -0.0720
   4 H    3.9940   3.4228   -9.6290 H     1  UNL1    0.1489
   5 H    5.7580   0.9299  -11.9331 H     1  UNL1    0.1643
   6 H    6.2850   1.5388  -10.3741 H     1  UNL1    0.1502
   7 H    5.6187   2.6525  -11.5770 H     1  UNL1    0.1487
   8 H    2.2961   0.9630  -13.5120 H     1  UNL1    0.1421
   9 H    3.9514   1.5875  -13.5160 H     1  UNL1    0.1398
  10 H    1.0129   0.6835  -11.5946 H     1  UNL1    0.1324
  11 H    1.1752   1.8657  -10.2913 H     1  UNL1    0.1237
  12 H    2.0463   0.2186   -8.8679 H     1  UNL1    0.1294
  13 H    1.7852  -0.9811  -10.1338 H     1  UNL1    0.1362
  14 H    2.8147   2.7950  -11.7446 H     1  UNL1    0.1167
  15 C    3.1114  -1.4662  -14.9220 C.3   1  UNL1   -0.4870
  16 C    4.4080  -2.3246  -11.4960 C.3   1  UNL1   -0.2619
  17 C    4.2982  -2.7058  -12.9949 C.3   1  UNL1   -0.2851
  18 C    4.1060  -1.3657  -13.7696 C.3   1  UNL1   -0.0359
  19 C    4.5879  -0.5891   -9.0003 C.3   1  UNL1   -0.5055
  20 C    3.9937   2.3715   -9.3292 C.3   1  UNL1   -0.5165
  21 C    5.5184   1.6521  -11.1463 C.3   1  UNL1   -0.5157
  22 C    3.2288   1.0036  -12.9152 C.3   1  UNL1   -0.2853
  23 C    3.7421  -0.3792  -12.6763 C.2   1  UNL1   -0.0438
  24 C    4.1228   1.4458  -10.5521 C.3   1  UNL1    0.1603
  25 C    2.9584   1.7151  -11.5666 C.3   1  UNL1   -0.1066
  26 C    1.7211   1.0966  -10.8604 C.3   1  UNL1   -0.2710
  27 H    3.0734   2.1961   -8.7647 H     1  UNL1    0.1549
  28 H    4.8294   2.2282   -8.6367 H     1  UNL1    0.1508
  29 H    4.5146   0.0796   -8.1355 H     1  UNL1    0.1539
  30 H    4.2290  -1.5720   -8.6820 H     1  UNL1    0.1506
  31 H    5.6512  -0.6822   -9.2455 H     1  UNL1    0.1555
  32 H    5.0940  -1.0482  -14.1900 H     1  UNL1    0.1200
  33 H    3.4399  -3.3766  -13.1584 H     1  UNL1    0.1350
  34 H    5.1856  -3.2528  -13.3418 H     1  UNL1    0.1272
  35 H    5.4329  -2.4696  -11.1150 H     1  UNL1    0.1368
```

```
    36 H          3.7446   -2.9492  -10.8740 H       1  UNL1        0.1363
    37 H          2.8181   -0.4767  -15.2922 H       1  UNL1        0.1505
    38 H          3.5492   -2.0093  -15.7675 H       1  UNL1        0.1463
    39 H          2.1931   -1.9871  -14.6292 H       1  UNL1        0.1542
@<TRIPOS>BOND
     1    38    15    1
     2    37    15    1
     3    15    39    1
     4    15    18    1
     5    32    18    1
     6    18    17    1
     7    18    23    1
     8     9    22    1
     9     8    22    1
    10    34    17    1
    11    33    17    1
    12    17    16    1
    13    22    23    1
    14    22    25    1
    15    23     3    2
    16     5    21    1
    17    14    25    1
    18    10    26    1
    19     7    21    1
    20    25    26    1
    21    25    24    1
    22    16     3    1
    23    16    35    1
    24    16    36    1
    25     3     2    1
    26    21    24    1
    27    21     6    1
    28    26    11    1
    29    26     1    1
    30    24     2    1
    31    24    20    1
    32     2     1    1
    33     2    19    1
    34    13     1    1
    35     1    12    1
    36     4    20    1
    37    20    27    1
    38    20    28    1
    39    31    19    1
    40    19    30    1
    41    19    29    1
```

Compound 28

```
@<TRIPOS>MOLECULE
compuesto_28.out
 39 41 0 0 0
SMALL
MULLIKEN_CHARGES

@<TRIPOS>ATOM
      1 C          3.0361   -0.0293  -10.7759 C.3     1  UNL1       -0.1418
      2 H          5.4735    0.5083  -12.7711 H       1  UNL1        0.1374
      3 H          3.8354    2.7116   -9.7469 H       1  UNL1        0.1482
      4 H          5.4220    2.2914  -10.4105 H       1  UNL1        0.1448
      5 H          5.1128   -0.0199  -10.0750 H       1  UNL1        0.1413
      6 H          3.9450    0.5079   -8.8561 H       1  UNL1        0.1275
      7 C          4.3488   -0.9404  -15.7417 C.3     1  UNL1       -0.4929
      8 C          5.1187    1.4085  -15.2497 C.3     1  UNL1       -0.4957
      9 C          3.9170    3.0983  -12.6754 C.2     1  UNL1       -0.4742
     10 H          2.1146   -0.1300  -10.1847 H       1  UNL1        0.1262
     11 H          1.8847    1.1599  -12.3070 H       1  UNL1        0.1386
```

```
   12 H        2.7897  -0.4993  -13.7835 H     1  UNL1       0.1319
   13 C        2.1390  -2.2119  -11.7731 C.3   1  UNL1      -0.5184
   14 C        5.2332  -0.0666  -14.8447 C.3   1  UNL1      -0.0051
   15 C        4.6571  -1.9989  -11.5726 C.3   1  UNL1      -0.3099
   16 C        5.4398  -1.6711  -12.8556 C.3   1  UNL1      -0.2725
   17 C        4.9324  -0.2873  -13.3435 C.3   1  UNL1      -0.1070
   18 C        3.3205  -1.2576  -11.7031 C.3   1  UNL1       0.1291
   19 C        3.4481  -0.2744  -12.9334 C.3   1  UNL1      -0.1363
   20 C        2.9194   0.8839  -12.0437 C.3   1  UNL1      -0.1784
   21 C        3.7665   2.1003  -11.8079 C.2   1  UNL1       0.1459
   22 C        4.3601   2.0075  -10.4172 C.3   1  UNL1      -0.3063
   23 C        4.1754   0.5540   -9.9304 C.3   1  UNL1      -0.2607
   24 H        5.2375  -2.4492  -13.6150 H     1  UNL1       0.1327
   25 H        6.5244  -1.6691  -12.6853 H     1  UNL1       0.1253
   26 H        5.2053  -1.6682  -10.6714 H     1  UNL1       0.1385
   27 H        4.5204  -3.0860  -11.4589 H     1  UNL1       0.1359
   28 H        6.3000  -0.3709  -15.0142 H     1  UNL1       0.1097
   29 H        2.2923  -2.9626  -12.5570 H     1  UNL1       0.1576
   30 H        1.9936  -2.7415  -10.8241 H     1  UNL1       0.1548
   31 H        1.2024  -1.6939  -12.0046 H     1  UNL1       0.1545
   32 H        3.4562   3.0982  -13.6555 H     1  UNL1       0.1640
   33 H        4.5132   3.9753  -12.5014 H     1  UNL1       0.1588
   34 H        5.3822   1.5490  -16.3031 H     1  UNL1       0.1449
   35 H        4.1028   1.7944  -15.1104 H     1  UNL1       0.1555
   36 H        5.7869   2.0453  -14.6587 H     1  UNL1       0.1515
   37 H        4.6029  -0.8144  -16.7967 H     1  UNL1       0.1459
   38 H        4.4599  -2.0009  -15.4933 H     1  UNL1       0.1472
   39 H        3.2874  -0.6896  -15.6228 H     1  UNL1       0.1517
@<TRIPOS>BOND
     1    37     7    1
     2    34     8    1
     3     7    39    1
     4     7    38    1
     5     7    14    1
     6     8    35    1
     7     8    14    1
     8     8    36    1
     9    28    14    1
    10    14    17    1
    11    12    19    1
    12    32     9    1
    13    24    16    1
    14    17    19    1
    15    17    16    1
    16    17     2    1
    17    19    20    1
    18    19    18    1
    19    16    25    1
    20    16    15    1
    21     9    33    1
    22     9    21    2
    23    29    13    1
    24    11    20    1
    25    20    21    1
    26    20     1    1
    27    31    13    1
    28    21    22    1
    29    13    18    1
    30    13    30    1
    31    18    15    1
    32    18     1    1
    33    15    27    1
    34    15    26    1
    35     1    10    1
    36     1    23    1
    37    22     4    1
```

```
   38    22    23    1
   39    22     3    1
   40     5    23    1
   41    23     6    1
```

Compound 29

```
@<TRIPOS>MOLECULE
compuesto_29.out
 39 39 0 0 0
SMALL
MULLIKEN_CHARGES

@<TRIPOS>ATOM
      1 C          5.0709    0.5780  -14.1253 C.3     1  UNL1       -0.1716
      2 C          4.4913    1.4051  -12.9262 C.3     1  UNL1        0.1658
      3 C          5.3103    2.7070  -12.7746 C.3     1  UNL1       -0.5169
      4 C          3.0068    1.7044  -13.0424 C.2     1  UNL1       -0.1178
      5 C          2.3313    2.5335  -12.2428 C.2     1  UNL1       -0.3713
      6 C          3.8219   -2.9669  -13.0719 C.2     1  UNL1        0.1280
      7 C          2.5067   -3.0969  -13.2796 C.2     1  UNL1       -0.4444
      8 C          4.7158   -4.1683  -12.9414 C.3     1  UNL1       -0.5111
      9 C          5.1661    1.2764  -15.4729 C.2     1  UNL1        0.1495
     10 C          4.3039    2.1889  -15.9333 C.2     1  UNL1       -0.4547
     11 C          6.3390    0.8461  -16.3187 C.3     1  UNL1       -0.5156
     12 H          6.1334    0.3684  -13.8275 H       1  UNL1        0.1378
     13 H          3.3306   -0.6842  -14.5598 H       1  UNL1        0.1456
     14 H          4.8706   -1.3840  -15.0705 H       1  UNL1        0.1327
     15 H          5.5982   -1.7898  -12.7697 H       1  UNL1        0.1281
     16 H          2.8790   -0.6618  -11.8325 H       1  UNL1        0.1448
     17 H          4.1301   -1.3914  -10.8158 H       1  UNL1        0.1290
     18 H          4.3034    1.0930  -10.7575 H       1  UNL1        0.1397
     19 H          5.7640    0.3865  -11.4563 H       1  UNL1        0.1339
     20 H          6.3927    2.5075  -12.7563 H       1  UNL1        0.1499
     21 H          5.1221    3.4007  -13.6068 H       1  UNL1        0.1665
     22 C          4.6831    0.5445  -11.6422 C.3     1  UNL1       -0.3086
     23 C          3.9744   -0.8125  -11.7472 C.3     1  UNL1       -0.2604
     24 C          4.5059   -1.6145  -12.9510 C.3     1  UNL1       -0.1147
     25 C          4.3881   -0.8033  -14.2599 C.3     1  UNL1       -0.2701
     26 H          5.0683    3.2420  -11.8425 H       1  UNL1        0.1534
     27 H          2.4650    1.1725  -13.8287 H       1  UNL1        0.1402
     28 H          1.2732    2.7057  -12.3534 H       1  UNL1        0.1473
     29 H          2.7926    3.0822  -11.4334 H       1  UNL1        0.1493
     30 H          2.0257   -4.0636  -13.3629 H       1  UNL1        0.1571
     31 H          1.8447   -2.2527  -13.3780 H       1  UNL1        0.1597
     32 H          5.2107   -4.1822  -11.9592 H       1  UNL1        0.1636
     33 H          4.1713   -5.1169  -13.0363 H       1  UNL1        0.1626
     34 H          5.4936   -4.1666  -13.7252 H       1  UNL1        0.1633
     35 H          4.4076    2.6461  -16.9036 H       1  UNL1        0.1556
     36 H          3.4398    2.5314  -15.3817 H       1  UNL1        0.1662
     37 H          6.3116   -0.2369  -16.5186 H       1  UNL1        0.1626
     38 H          6.3594    1.3606  -17.3024 H       1  UNL1        0.1635
     39 H          7.2947    1.0741  -15.8227 H       1  UNL1        0.1611
@<TRIPOS>BOND
    1    38    11    1
    2    35    10    1
    3    37    11    1
    4    11    39    1
    5    11     9    1
    6    10     9    2
    7    10    36    1
    8     9     1    1
    9    14    25    1
   10    13    25    1
   11    25     1    1
   12    25    24    1
   13     1    12    1
```

```
    14    1    2   1
    15   27    4   1
    16   34    8   1
    17   21    3   1
    18   31    7   1
    19   30    7   1
    20    7    6   2
    21    6   24   1
    22    6    8   1
    23    4    2   1
    24    4    5   2
    25   33    8   1
    26   24   15   1
    27   24   23   1
    28    8   32   1
    29    2    3   1
    30    2   22   1
    31    3   20   1
    32    3   26   1
    33   28    5   1
    34    5   29   1
    35   16   23   1
    36   23   22   1
    37   23   17   1
    38   22   19   1
    39   22   18   1
```

Compound 30

```
@<TRIPOS>MOLECULE
compuesto_30.out
 39 40 0 0 0
SMALL
MULLIKEN_CHARGES

@<TRIPOS>ATOM
      1 C          5.2284    0.7420  -15.5513 C.3     1  UNL1        0.1899
      2 C          4.6275   -0.6908  -15.6962 C.3     1  UNL1       -0.3571
      3 C          3.9053   -0.4950  -14.3353 C.3     1  UNL1       -0.1361
      4 C          4.9218    0.6506  -14.0060 C.3     1  UNL1       -0.1346
      5 C          4.0370    0.4934  -11.3172 C.3     1  UNL1        0.1255
      6 C          4.5003    1.8558  -11.7691 C.3     1  UNL1       -0.3112
      7 C          4.4180    1.9124  -13.3114 C.3     1  UNL1       -0.2535
      8 C          2.5616    0.3052  -11.2299 C.3     1  UNL1       -0.4941
      9 C          4.9684   -0.4646  -11.1647 C.3     1  UNL1       -0.2786
     10 C          4.7141   -1.9345  -11.2229 C.3     1  UNL1       -0.2117
     11 C          4.8012   -2.3510  -12.7104 C.3     1  UNL1       -0.3030
     12 C          3.6901   -1.7409  -13.5286 C.2     1  UNL1        0.1423
     13 C          2.4897   -2.3333  -13.5696 C.2     1  UNL1       -0.4707
     14 C          4.3980    1.7790  -16.3078 C.3     1  UNL1       -0.5156
     15 C          6.6990    0.8691  -15.9077 C.3     1  UNL1       -0.5242
     16 H          5.8063    0.2592  -13.4625 H       1  UNL1        0.1431
     17 H          2.9034   -0.0274  -14.4988 H       1  UNL1        0.1360
     18 H          3.9938   -0.8762  -16.5595 H       1  UNL1        0.1426
     19 H          5.3900   -1.4788  -15.6819 H       1  UNL1        0.1458
     20 H          3.8899    2.6673  -11.3299 H       1  UNL1        0.1390
     21 H          5.5378    2.0569  -11.4363 H       1  UNL1        0.1412
     22 H          3.3740    2.1227  -13.6103 H       1  UNL1        0.1327
     23 H          5.0157    2.7749  -13.6674 H       1  UNL1        0.1320
     24 H          2.0887    0.4516  -12.2133 H       1  UNL1        0.1672
     25 H          2.2863   -0.7060  -10.9012 H       1  UNL1        0.1639
     26 H          2.1016    1.0211  -10.5386 H       1  UNL1        0.1552
     27 H          6.0272   -0.2035  -11.1581 H       1  UNL1        0.1438
     28 H          5.4650   -2.4935  -10.6332 H       1  UNL1        0.1259
     29 H          3.7273   -2.2060  -10.8014 H       1  UNL1        0.1363
     30 H          4.7604   -3.4554  -12.7882 H       1  UNL1        0.1405
     31 H          5.7923   -2.0515  -13.1230 H       1  UNL1        0.1459
```

```
    32 H          1.6573   -1.9599  -14.1424 H      1  UNL1        0.1550
    33 H          2.2569   -3.2382  -13.0385 H      1  UNL1        0.1566
    34 H          4.7143    2.8015  -16.0832 H      1  UNL1        0.1527
    35 H          4.4843    1.6386  -17.3904 H      1  UNL1        0.1540
    36 H          3.3324    1.7082  -16.0594 H      1  UNL1        0.1549
    37 H          7.0919    1.8495  -15.6192 H      1  UNL1        0.1565
    38 H          7.3083    0.1103  -15.4065 H      1  UNL1        0.1547
    39 H          6.8568    0.7538  -16.9861 H      1  UNL1        0.1570
@<TRIPOS>BOND
     1    35    14    1
     2    39    15    1
     3    18     2    1
     4    14    34    1
     5    14    36    1
     6    14     1    1
     7    15    37    1
     8    15     1    1
     9    15    38    1
    10     2    19    1
    11     2     1    1
    12     2     3    1
    13     1     4    1
    14    17     3    1
    15     3     4    1
    16     3    12    1
    17    32    13    1
    18     4    16    1
    19     4     7    1
    20    23     7    1
    21    22     7    1
    22    13    12    2
    23    13    33    1
    24    12    11    1
    25     7     6    1
    26    31    11    1
    27    30    11    1
    28    11    10    1
    29    24     8    1
    30     6    21    1
    31     6    20    1
    32     6     5    1
    33     5     8    1
    34     5     9    1
    35     8    25    1
    36     8    26    1
    37    10     9    1
    38    10    29    1
    39    10    28    1
    40     9    27    1
```

Compound 31

```
@<TRIPOS>MOLECULE
compuesto_31.out
 39 41 0 0 0
SMALL
MULLIKEN_CHARGES

@<TRIPOS>ATOM
      1 C          5.4170   -1.3561  -15.8449 C.3    1  UNL1       -0.2662
      2 C          3.9966    1.9975  -13.3650 C.3    1  UNL1       -0.0355
      3 C          2.5816   -2.2878  -13.9787 C.2    1  UNL1       -0.4558
      4 C          5.1569   -2.1360  -12.3390 C.3    1  UNL1       -0.5048
      5 C          6.5844    0.5591  -12.6721 C.3    1  UNL1       -0.5206
      6 C          3.2555    3.1583  -14.0410 C.3    1  UNL1       -0.4997
      7 H          5.4819   -2.4558  -15.8909 H      1  UNL1        0.1297
      8 H          5.7161   -0.9888  -16.8418 H      1  UNL1        0.1282
```

```
     9 H          3.2851   -1.3142  -16.3187 H       1  UNL1        0.1227
    10 H          7.1243   -0.1574  -15.2225 H       1  UNL1        0.1313
    11 H          6.9007   -1.6310  -14.2752 H       1  UNL1        0.1344
    12 H          4.2404    1.0170  -16.4963 H       1  UNL1        0.1275
    13 H          2.8672    0.9522  -15.3985 H       1  UNL1        0.1393
    14 H          5.5689    1.8553  -14.8476 H       1  UNL1        0.1189
    15 H          4.3203    0.1647  -11.1511 H       1  UNL1        0.1332
    16 H          2.8947   -0.7904  -11.5584 H       1  UNL1        0.1347
    17 H          2.1801    0.7961  -13.2661 H       1  UNL1        0.1415
    18 H          2.5250    1.6942  -11.7862 H       1  UNL1        0.1246
    19 H          4.7236    2.4323  -12.6346 H       1  UNL1        0.1145
    20 H          2.2857   -2.6827  -13.0217 H       1  UNL1        0.1560
    21 H          1.9752   -2.6599  -14.7873 H       1  UNL1        0.1534
    22 H          5.8623   -1.8200  -11.5654 H       1  UNL1        0.1501
    23 H          4.4117   -2.7853  -11.8694 H       1  UNL1        0.1527
    24 H          5.7074   -2.7549  -13.0582 H       1  UNL1        0.1567
    25 H          7.1993   -0.2413  -12.2491 H       1  UNL1        0.1525
    26 H          7.2636    1.2790  -13.1395 H       1  UNL1        0.1506
    27 H          2.4577    2.7984  -14.7015 H       1  UNL1        0.1515
    28 C          4.4977   -0.9290  -13.0331 C.3     1  UNL1        0.0984
    29 C          6.3567   -0.8006  -14.7626 C.3     1  UNL1       -0.3065
    30 C          3.5934   -1.4391  -14.1584 C.2     1  UNL1        0.0855
    31 C          3.9697   -0.9214  -15.5358 C.3     1  UNL1       -0.0980
    32 C          4.8113    1.1767  -14.3948 C.3     1  UNL1       -0.1272
    33 C          3.9139    0.6174  -15.5219 C.3     1  UNL1       -0.2851
    34 C          5.5742    0.0079  -13.6892 C.3     1  UNL1        0.1305
    35 C          3.0024    1.1088  -12.5932 C.3     1  UNL1       -0.2811
    36 C          3.6697   -0.1327  -11.9947 C.3     1  UNL1       -0.2916
    37 H          6.0946    1.0699  -11.8343 H       1  UNL1        0.1580
    38 H          2.7916    3.8177  -13.3019 H       1  UNL1        0.1470
    39 H          3.9276    3.7699  -14.6505 H       1  UNL1        0.1485
@<TRIPOS>BOND
     1     8     1    1
     2    12    33    1
     3     9    31    1
     4     7     1    1
     5     1    31    1
     6     1    29    1
     7    31    33    1
     8    31    30    1
     9    33    13    1
    10    33    32    1
    11    10    29    1
    12    14    32    1
    13    21     3    1
    14    29    11    1
    15    29    34    1
    16    27     6    1
    17    39     6    1
    18    32    34    1
    19    32     2    1
    20    30     3    2
    21    30    28    1
    22     6     2    1
    23     6    38    1
    24     3    20    1
    25    34    28    1
    26    34     5    1
    27     2    19    1
    28     2    35    1
    29    17    35    1
    30    26     5    1
    31    24     4    1
    32    28     4    1
    33    28    36    1
    34     5    25    1
```

```
35    5   37   1
36   35   36   1
37   35   18   1
38    4   23   1
39    4   22   1
40   36   16   1
41   36   15   1
```

Compound 32

```
@<TRIPOS>MOLECULE
compuesto_32.out
 39 40 0 0 0
SMALL
MULLIKEN_CHARGES

@<TRIPOS>ATOM
      1 H          4.5053    3.1892  -15.6032 H       1  UNL1        0.1566
      2 H          3.1962    2.6812  -14.4792 H       1  UNL1        0.1586
      3 H          6.4147    0.2791  -15.4278 H       1  UNL1        0.1638
      4 H          6.5905    2.0086  -15.7524 H       1  UNL1        0.1629
      5 H          7.1119    1.3496  -14.1958 H       1  UNL1        0.1640
      6 C          3.3163   -0.0245   -8.6058 C.3     1  UNL1       -0.2884
      7 C          3.8514   -1.2978   -9.2946 C.3     1  UNL1       -0.2693
      8 C          3.7570   -0.9710  -10.7724 C.2    1  UNL1       -0.0553
      9 C          3.8962   -2.0364  -11.8264 C.3    1  UNL1       -0.0226
     10 C          3.1164   -1.6625  -13.1071 C.3    1  UNL1       -0.2792
     11 C          3.8472   -0.6840  -14.0341 C.3    1  UNL1       -0.2705
     12 C          4.5333    0.5038  -13.3299 C.3    1  UNL1       -0.1024
     13 C          3.6494    1.1362  -12.2338 C.3    1  UNL1       -0.2763
     14 C          3.6300    0.3591  -10.9576 C.2    1  UNL1       -0.0533
     15 C          3.5337    1.1149   -9.6360 C.3     1  UNL1       -0.0364
     16 C          2.4217    2.1618   -9.5774 C.3     1  UNL1       -0.4877
     17 C          4.9675    1.5041  -14.3950 C.2    1  UNL1        0.1341
     18 C          3.4264   -3.4184  -11.3379 C.3    1  UNL1       -0.4914
     19 C          4.1889    2.4966  -14.8369 C.2    1  UNL1       -0.4557
     20 C          6.3341    1.2759  -14.9685 C.3    1  UNL1       -0.5134
     21 H          2.6111    1.2886  -12.5926 H       1  UNL1        0.1500
     22 H          4.0442    2.1541  -12.0201 H       1  UNL1        0.1448
     23 H          5.4578    0.1281  -12.8165 H       1  UNL1        0.1368
     24 H          3.1161   -0.3009  -14.7767 H       1  UNL1        0.1374
     25 H          4.6159   -1.2364  -14.6122 H       1  UNL1        0.1276
     26 H          2.1246   -1.2564  -12.8204 H       1  UNL1        0.1427
     27 H          2.9003   -2.5757  -13.6978 H       1  UNL1        0.1287
     28 H          4.9883   -2.1183  -12.0732 H       1  UNL1        0.1254
     29 H          3.2634   -2.1767   -8.9933 H       1  UNL1        0.1373
     30 H          4.9051   -1.5039   -9.0336 H       1  UNL1        0.1374
     31 H          3.7930    0.1764   -7.6375 H       1  UNL1        0.1290
     32 H          2.2330   -0.1503   -8.4161 H       1  UNL1        0.1384
     33 H          4.5177    1.6123   -9.4529 H       1  UNL1        0.1217
     34 H          2.3533    2.6000   -8.5708 H       1  UNL1        0.1484
     35 H          2.5969    2.9832  -10.2829 H       1  UNL1        0.1493
     36 H          1.4423    1.7262   -9.8202 H       1  UNL1        0.1550
     37 H          3.5311   -4.1759  -12.1240 H       1  UNL1        0.1472
     38 H          4.0198   -3.7684  -10.4842 H       1  UNL1        0.1491
     39 H          2.3670   -3.3650  -11.0405 H       1  UNL1        0.1556
@<TRIPOS>BOND
     1    4   20   1
     2    1   19   1
     3    3   20   1
     4   20   17   1
     5   20    5   1
     6   19    2   1
     7   19   17   2
     8   24   11   1
     9   25   11   1
    10   17   12   1
```

```
11 11 12 1
12 11 10 1
13 27 10 1
14 12 23 1
15 12 13 1
16 10 26 1
17 10 9 1
18 21 13 1
19 13 22 1
20 13 14 1
21 37 18 1
22 28 9 1
23 9 18 1
24 9 8 1
25 18 39 1
26 18 38 1
27 14 8 2
28 14 15 1
29 8 7 1
30 35 16 1
31 36 16 1
32 15 16 1
33 15 33 1
34 15 6 1
35 16 34 1
36 7 30 1
37 7 29 1
38 7 6 1
39 6 32 1
40 6 31 1
```

Compound 33

```
@<TRIPOS>MOLECULE
compuesto_33.out
 39 39 0 0 0
SMALL
MULLIKEN_CHARGES

@<TRIPOS>ATOM
 1 C 2.8893 1.5406 -13.0696 C.3 1 UNL1 -0.3143
 2 C 3.1264 0.7519 -11.7333 C.3 1 UNL1 0.2366
 3 C 4.2607 -0.2245 -11.9679 C.2 1 UNL1 -0.2052
 4 C 4.1961 -1.5585 -11.9550 C.2 1 UNL1 -0.1420
 5 C 4.6240 1.4241 -14.8915 C.2 1 UNL1 0.0820
 6 C 5.2612 0.7959 -16.1114 C.3 1 UNL1 -0.2960
 7 C 5.3110 2.6751 -14.4265 C.3 1 UNL1 -0.5001
 8 H 6.2639 1.2403 -16.2787 H 1 UNL1 0.1401
 9 H 4.6637 1.0761 -17.0043 H 1 UNL1 0.1408
 10 H 6.1922 -1.0582 -16.7858 H 1 UNL1 0.1309
 11 H 4.4713 -1.2059 -16.4285 H 1 UNL1 0.1330
 12 H 3.3282 -2.1148 -11.6167 H 1 UNL1 0.1369
 13 H 6.4830 -0.6020 -14.1662 H 1 UNL1 0.1507
 14 H 3.0700 0.0035 -14.6179 H 1 UNL1 0.1526
 15 H 5.1826 0.2789 -12.2787 H 1 UNL1 0.1530
 16 H 1.8022 1.6320 -13.2579 H 1 UNL1 0.1422
 17 H 3.2462 2.5859 -12.9390 H 1 UNL1 0.1399
 18 H 6.2958 -2.0653 -12.2205 H 1 UNL1 0.1495
 19 H 5.2163 -3.4554 -12.2551 H 1 UNL1 0.1430
 20 H 4.2372 -3.1653 -15.7967 H 1 UNL1 0.1569
 21 H 4.2671 -4.1796 -14.3515 H 1 UNL1 0.1567
 22 H 3.1185 -2.8378 -14.4618 H 1 UNL1 0.1646
 23 H 3.4602 2.7808 -10.9296 H 1 UNL1 0.1509
 24 H 2.8936 1.6306 -9.7262 H 1 UNL1 0.1561
 25 H 4.5607 1.5751 -10.2751 H 1 UNL1 0.1571
 26 H 1.4964 -0.6603 -12.1144 H 1 UNL1 0.1565
 27 H 1.9339 -0.5183 -10.4057 H 1 UNL1 0.1546
```

```
  28 H        1.0061   0.7648  -11.1908 H     1  UNL1     0.1514
  29 H        4.8062   3.1242  -13.5569 H     1  UNL1     0.1606
  30 H        6.3441   2.4655  -14.1207 H     1  UNL1     0.1594
  31 H        5.3408   3.4356  -15.2153 H     1  UNL1     0.1560
  32 C        1.8179   0.0443  -11.3381 C.3   1  UNL1    -0.5257
  33 C        3.5311   1.7368  -10.6097 C.3   1  UNL1    -0.5304
  34 C        4.1464  -3.1484  -14.7029 C.3   1  UNL1    -0.4926
  35 C        5.2956  -2.3971  -12.5608 C.3   1  UNL1    -0.3088
  36 C        5.1333  -2.2279  -14.0590 C.2   1  UNL1     0.0931
  37 C        3.5377   0.9277  -14.2785 C.2   1  UNL1    -0.2199
  38 C        5.7470  -1.2144  -14.6887 C.2   1  UNL1    -0.2425
  39 C        5.4107  -0.7384  -16.0674 C.3   1  UNL1    -0.2276
@<TRIPOS>BOND
   1    9    6    1
   2   10   39    1
   3   11   39    1
   4    8    6    1
   5    6   39    1
   6    6    5    1
   7   39   38    1
   8   20   34    1
   9   31    7    1
  10    5    7    1
  11    5   37    2
  12   34   22    1
  13   34   21    1
  14   34   36    1
  15   38   13    1
  16   38   36    2
  17   14   37    1
  18    7   30    1
  19    7   29    1
  20   37    1    1
  21   36   35    1
  22   16    1    1
  23    1   17    1
  24    1    2    1
  25   35   19    1
  26   35   18    1
  27   35    4    1
  28   15    3    1
  29   26   32    1
  30    3    4    2
  31    3    2    1
  32    4   12    1
  33    2   32    1
  34    2   33    1
  35   32   28    1
  36   32   27    1
  37   23   33    1
  38   33   25    1
  39   33   24    1
```

Compound 34

```
@<TRIPOS>MOLECULE
compuesto_34.out
 39 41 0 0 0
SMALL
MULLIKEN_CHARGES

@<TRIPOS>ATOM
   1 H        1.9233  -2.2260  -12.0313 H     1  UNL1     0.1558
   2 C        4.0344  -0.4267  -14.1411 C.3   1  UNL1    -0.2043
   3 H        3.1581   3.9770  -14.6372 H     1  UNL1     0.1555
   4 H        3.6786   3.2482  -16.1939 H     1  UNL1     0.1588
   5 H        4.9602  -2.6956  -15.3506 H     1  UNL1     0.1580
```

```
 6 H      6.4203  -2.7001  -14.3610 H     1  UNL1     0.1490
 7 H      6.5418  -2.9461  -16.1181 H     1  UNL1     0.1456
 8 H      7.2679  -0.6557  -15.4567 H     1  UNL1     0.1106
 9 H      4.8311  -1.0066  -17.2592 H     1  UNL1     0.1355
10 H      6.4409  -0.3652  -17.6428 H     1  UNL1     0.1276
11 H      5.5388   1.8069  -17.2930 H     1  UNL1     0.1263
12 H      4.0155   1.0744  -16.7516 H     1  UNL1     0.1388
13 H      5.6951   2.8253  -12.6654 H     1  UNL1     0.1413
14 H      4.0006   3.3478  -12.5897 H     1  UNL1     0.1430
15 H      5.1906   0.7454  -11.7908 H     1  UNL1     0.1305
16 H      3.7215   1.5263  -11.2257 H     1  UNL1     0.1275
17 H      2.3755   0.6561  -13.0719 H     1  UNL1     0.1493
18 H      3.4240  -0.5787  -15.0405 H     1  UNL1     0.1535
19 H      6.0746  -0.0053  -13.5353 H     1  UNL1     0.1203
20 H      6.4952   1.7958  -15.0934 H     1  UNL1     0.1312
21 C      4.6691  -1.8371  -11.9858 C.3   1  UNL1    -0.4809
22 C      2.3836  -2.0822  -13.0162 C.3   1  UNL1    -0.4941
23 C      3.6351  -1.2240  -12.8988 C.3   1  UNL1     0.1130
24 C      3.7710   3.2373  -15.1237 C.2   1  UNL1    -0.4683
25 C      6.0116  -2.3916  -15.3312 C.3   1  UNL1    -0.4874
26 C      6.1737  -0.8908  -15.5286 C.3   1  UNL1    -0.0449
27 C      5.6439  -0.3610  -16.8820 C.3   1  UNL1    -0.2899
28 C      5.1125   1.0622  -16.6042 C.3   1  UNL1    -0.2617
29 C      5.4650  -0.0226  -14.4598 C.3   1  UNL1    -0.0753
30 C      5.4534   1.3867  -15.1257 C.3   1  UNL1    -0.1366
31 C      4.5636   2.4052  -14.4440 C.2   1  UNL1     0.1394
32 C      4.6494   2.5150  -12.9372 C.3   1  UNL1    -0.3104
33 C      4.2612   1.2474  -12.1512 C.3   1  UNL1    -0.2236
34 C      3.3996   0.2895  -12.9479 C.3   1  UNL1    -0.2055
35 H      1.6179  -1.6303  -13.6739 H     1  UNL1     0.1544
36 H      2.6249  -3.0669  -13.4383 H     1  UNL1     0.1542
37 H      4.8802  -2.8847  -12.2940 H     1  UNL1     0.1562
38 H      5.6281  -1.2999  -11.9886 H     1  UNL1     0.1536
39 H      4.2979  -1.8597  -10.9521 H     1  UNL1     0.1538
@<TRIPOS>BOND
 1   10   27   1
 2   11   28   1
 3    9   27   1
 4   27   28   1
 5   27   26   1
 6   12   28   1
 7   28   30   1
 8    4   24   1
 9    7   25   1
10   26    8   1
11   26   25   1
12   26   29   1
13    5   25   1
14   25    6   1
15   30   20   1
16   30   29   1
17   30   31   1
18   24    3   1
19   24   31   2
20   18    2   1
21   29    2   1
22   29   19   1
23   31   32   1
24    2   34   1
25    2   23   1
26   35   22   1
27   36   22   1
28   17   34   1
29   22   23   1
30   22    1   1
31   34   23   1
```

```
 32   34   33   1
 33   32   13   1
 34   32   14   1
 35   32   33   1
 36   23   21   1
 37   37   21   1
 38   33   15   1
 39   33   16   1
 40   38   21   1
 41   21   39   1
```

Compound 35

```
@<TRIPOS>MOLECULE
compuesto_35.out
 39 41 0 0 0
SMALL
MULLIKEN_CHARGES

@<TRIPOS>ATOM
   1 H    6.1934  -1.9008  -15.8739 H    1  UNL1   0.1516
   2 H    4.9196  -2.1422  -17.0704 H    1  UNL1   0.1479
   3 C    3.6139  -2.1325  -14.5769 C.3  1  UNL1  -0.2829
   4 H    5.9799  -0.4658  -14.0849 H    1  UNL1   0.1310
   5 C    5.3312  -1.4229  -16.3567 C.3  1  UNL1  -0.4972
   6 H    4.3023  -2.9906  -14.4955 H    1  UNL1   0.1321
   7 H    2.7180  -2.4904  -15.1194 H    1  UNL1   0.1265
   8 H    2.1717  -1.3479  -13.1210 H    1  UNL1   0.1370
   9 H    3.4045  -2.3775  -12.4120 H    1  UNL1   0.1335
  10 H    3.4908  -0.3853  -15.8725 H    1  UNL1   0.1168
  11 H    6.6120   0.7340  -10.7675 H    1  UNL1   0.1389
  12 H    5.2874   2.7978  -11.3414 H    1  UNL1   0.1331
  13 H    6.4616   2.4324  -12.6115 H    1  UNL1   0.1356
  14 H    3.9904   3.0341  -13.3408 H    1  UNL1   0.1176
  15 H    4.3620   1.7153  -15.3744 H    1  UNL1   0.1322
  16 H    6.0021   1.7545  -14.7078 H    1  UNL1   0.1324
  17 H    5.8074  -2.4348  -11.8941 H    1  UNL1   0.1637
  18 H    6.1869  -1.6234  -10.3674 H    1  UNL1   0.1550
  19 H    4.5307  -2.1343  -10.6964 H    1  UNL1   0.1601
  20 H    2.4083   0.9819  -14.7866 H    1  UNL1   0.1576
  21 H    1.3305   0.3523  -13.5182 H    1  UNL1   0.1514
  22 H    1.6188   2.1082  -13.6648 H    1  UNL1   0.1482
  23 H    2.0592   0.3674  -11.0640 H    1  UNL1   0.1513
  24 H    3.5413   1.2131  -10.5790 H    1  UNL1   0.1656
  25 H    2.1829   2.1438  -11.2563 H    1  UNL1   0.1486
  26 H    5.7081  -0.5523  -16.9105 H    1  UNL1   0.1503
  27 C    3.2837   1.0056  -12.7646 C.3  1  UNL1   0.1698
  28 C    2.7404   1.1985  -11.3436 C.3  1  UNL1  -0.5178
  29 C    2.0938   1.1161  -13.7379 C.3  1  UNL1  -0.5160
  30 C    5.4316  -1.7074  -11.1591 C.3  1  UNL1  -0.4905
  31 C    4.9688   1.4163  -14.4985 C.3  1  UNL1  -0.3054
  32 C    4.4015   2.0219  -13.1848 C.3  1  UNL1  -0.1065
  33 C    5.5316   2.0600  -12.1304 C.3  1  UNL1  -0.2618
  34 C    5.8100   0.7232  -11.4984 C.2  1  UNL1  -0.2451
  35 C    5.1308  -0.3967  -11.8112 C.2  1  UNL1   0.0529
  36 C    4.2830  -0.9691  -15.3415 C.3  1  UNL1  -0.0433
  37 C    4.9398  -0.1039  -14.2484 C.3  1  UNL1  -0.1130
  38 C    4.1006  -0.3331  -12.9310 C.3  1  UNL1   0.0278
  39 C    3.2511  -1.5943  -13.1736 C.3  1  UNL1  -0.2890
@<TRIPOS>BOND
  1    2    5   1
  2   26    5   1
  3    5    1   1
  4    5   36   1
  5   10   36   1
  6   15   31   1
  7   36    3   1
```

```
   8   36   37   1
   9    7    3   1
  10   20   29   1
  11   16   31   1
  12    3    6   1
  13    3   39   1
  14   31   37   1
  15   31   32   1
  16   37    4   1
  17   37   38   1
  18   29   22   1
  19   29   21   1
  20   29   27   1
  21   14   32   1
  22   32   27   1
  23   32   33   1
  24   39    8   1
  25   39   38   1
  26   39    9   1
  27   38   27   1
  28   38   35   1
  29   27   28   1
  30   13   33   1
  31   33   34   1
  32   33   12   1
  33   17   30   1
  34   35   34   2
  35   35   30   1
  36   34   11   1
  37   28   25   1
  38   28   23   1
  39   28   24   1
  40   30   19   1
  41   30   18   1
```

Compound 36

```
@<TRIPOS>MOLECULE
compuesto_36.out
 39 40 0 0 0
SMALL
MULLIKEN_CHARGES

@<TRIPOS>ATOM
      1 C          4.8247    1.1208  -13.3629 C.3     1  UNL1       -0.3029
      2 H          5.5614    1.6549  -13.9967 H       1  UNL1        0.1409
      3 H          4.2275    0.4632  -14.0446 H       1  UNL1        0.1543
      4 H          6.1054    0.9607  -11.6422 H       1  UNL1        0.1315
      5 H          6.3321   -0.3453  -12.7975 H       1  UNL1        0.1299
      6 H          4.9774   -0.5903  -10.4290 H       1  UNL1        0.1269
      7 H          1.9627   -1.4068  -10.1791 H       1  UNL1        0.1474
      8 H          1.8743    2.4584   -8.9839 H       1  UNL1        0.1446
      9 H          3.2875    1.9366   -8.0354 H       1  UNL1        0.1423
     10 H          3.7710    3.2553  -10.2289 H       1  UNL1        0.1316
     11 H          4.7034    1.7676   -9.9660 H       1  UNL1        0.1383
     12 H          4.2558    3.7350  -13.9610 H       1  UNL1        0.1573
     13 H          3.0718    4.0438  -12.6058 H       1  UNL1        0.1595
     14 H          5.7531   -2.3632  -11.8572 H       1  UNL1        0.1056
     15 H          1.0967   -1.1383   -7.9842 H       1  UNL1        0.1597
     16 H          2.0938   -0.0820   -6.9707 H       1  UNL1        0.1615
     17 H          0.5863    0.5382   -7.7014 H       1  UNL1        0.1615
     18 H          4.3688   -3.4130  -13.6544 H       1  UNL1        0.1456
     19 H          3.1870   -2.0903  -13.5640 H       1  UNL1        0.1505
     20 H          4.8575   -1.7720  -14.1044 H       1  UNL1        0.1477
     21 H          4.2708   -4.1214  -11.1966 H       1  UNL1        0.1445
     22 H          4.1231   -2.8723   -9.9418 H       1  UNL1        0.1519
     23 H          2.8458   -3.0726  -11.1765 H       1  UNL1        0.1521
```

```
    24 C          3.9352   -3.0847   -11.0091 C.3     1  UNL1       -0.4958
    25 C          4.2461   -2.3452   -13.3915 C.3     1  UNL1       -0.4912
    26 C          1.4621   -0.1084    -7.8747 C.3     1  UNL1       -0.5055
    27 H          2.5675   -0.3532   -12.2902 H       1  UNL1        0.1255
    28 H          2.0810    1.8778   -11.5904 H       1  UNL1        0.1396
    29 C          4.6660   -2.0991   -11.9332 C.3     1  UNL1       -0.0016
    30 C          3.7439    3.3480   -13.1039 C.2     1  UNL1       -0.4695
    31 C          3.6811    2.1508   -10.1614 C.3     1  UNL1       -0.2470
    32 C          2.7541    1.7790    -8.9955 C.3     1  UNL1       -0.3012
    33 C          2.2383    0.3649    -9.0750 C.2     1  UNL1        0.1010
    34 C          2.4072   -0.4143   -10.1596 C.2     1  UNL1       -0.2846
    35 C          3.8988    2.0882   -12.6779 C.2     1  UNL1        0.1168
    36 C          3.1463    1.5600   -11.4790 C.3     1  UNL1       -0.1362
    37 C          3.1516    0.0093   -11.4077 C.3     1  UNL1       -0.0544
    38 C          4.5781   -0.6053   -11.4755 C.3     1  UNL1       -0.1128
    39 C          5.5476    0.2715   -12.3123 C.3     1  UNL1       -0.2653
@<TRIPOS>BOND
     1    20    25    1
     2     3     1    1
     3     2     1    1
     4    12    30    1
     5    18    25    1
     6    19    25    1
     7    25    29    1
     8     1    35    1
     9     1    39    1
    10    30    35    2
    11    30    13    1
    12     5    39    1
    13    35    36    1
    14    39     4    1
    15    39    38    1
    16    27    37    1
    17    29    14    1
    18    29    38    1
    19    29    24    1
    20    28    36    1
    21    36    37    1
    22    36    31    1
    23    38    37    1
    24    38     6    1
    25    37    34    1
    26    21    24    1
    27    23    24    1
    28    24    22    1
    29    10    31    1
    30     7    34    1
    31    31    11    1
    32    31    32    1
    33    34    33    2
    34    33    32    1
    35    33    26    1
    36    32     8    1
    37    32     9    1
    38    15    26    1
    39    26    17    1
    40    26    16    1
```

Compound 37

```
@<TRIPOS>MOLECULE
compuesto_37.out
 39 39 0 0 0
SMALL
MULLIKEN_CHARGES

@<TRIPOS>ATOM
```

```
     1 C        4.5953    1.1146  -14.2334 C.2     1  UNL1       -0.2397
     2 H        3.7632   -3.6251  -12.8606 H       1  UNL1        0.1505
     3 H        5.4458   -3.9741  -13.2329 H       1  UNL1        0.1490
     4 H        2.3682    0.4188   -8.3135 H       1  UNL1        0.1578
     5 H        2.5448   -1.0771   -9.2360 H       1  UNL1        0.1633
     6 H        0.9668   -0.2863   -9.1259 H       1  UNL1        0.1604
     7 H        6.1081   -1.6088  -12.8720 H       1  UNL1        0.1170
     8 H        5.5547    4.3862  -13.7986 H       1  UNL1        0.1573
     9 H        5.3531    3.4409  -15.3102 H       1  UNL1        0.1556
    10 H        3.2969    3.7582  -11.2808 H       1  UNL1        0.1308
    11 H        2.7330    2.5176  -12.4085 H       1  UNL1        0.1427
    12 H        3.3919    2.2013   -9.4362 H       1  UNL1        0.1461
    13 H        1.8361    0.8250  -12.5626 H       1  UNL1        0.1536
    14 H        0.7936   -0.2545  -11.6205 H       1  UNL1        0.1392
    15 H        5.2834    1.5308  -11.5411 H       1  UNL1        0.1556
    16 H        5.5175    3.3007  -11.6580 H       1  UNL1        0.1434
    17 H        2.6767   -1.9184  -11.4036 H       1  UNL1        0.1346
    18 H        2.2310   -1.6046  -13.0813 H       1  UNL1        0.1296
    19 H        4.4985   -0.0310  -11.6921 H       1  UNL1        0.1545
    20 H        3.9948   -0.8394  -14.6374 H       1  UNL1        0.1322
    21 H        4.5956    1.2872  -15.3241 H       1  UNL1        0.1481
    22 H        4.5099   -2.9207  -14.2972 H       1  UNL1        0.1477
    23 H        4.5262   -2.7771  -10.4855 H       1  UNL1        0.1500
    24 H        6.1808   -3.1651  -10.9378 H       1  UNL1        0.1493
    25 H        5.7736   -1.5379  -10.3856 H       1  UNL1        0.1500
    26 C        5.4162   -2.3824  -10.9838 C.3     1  UNL1       -0.4976
    27 C        4.6852   -3.1842  -13.2500 C.3     1  UNL1       -0.4936
    28 C        2.0440   -0.1007   -9.2229 C.3     1  UNL1       -0.5043
    29 C        5.1402   -1.9679  -12.4334 C.3     1  UNL1       -0.0014
    30 C        5.2982    3.4308  -14.2310 C.2     1  UNL1       -0.4449
    31 C        3.4261    2.6911  -11.5554 C.3     1  UNL1       -0.2221
    32 C        3.0484    1.8209  -10.3976 C.2     1  UNL1       -0.2743
    33 C        2.3533    0.6743  -10.4684 C.2     1  UNL1        0.1109
    34 C        1.8481    0.0629  -11.7476 C.3     1  UNL1       -0.3064
    35 C        4.9498    2.3520  -13.5109 C.2     1  UNL1        0.1399
    36 C        4.8832    2.4564  -12.0095 C.3     1  UNL1       -0.3015
    37 C        2.6965   -1.1411  -12.1914 C.3     1  UNL1       -0.2432
    38 C        4.1627   -0.7527  -12.4863 C.3     1  UNL1       -0.1242
    39 C        4.2577   -0.1235  -13.8494 C.2     1  UNL1       -0.1159
@<TRIPOS>BOND
     1    21     1    1
     2     9    30    1
     3    20    39    1
     4    22    27    1
     5     1    39    2
     6     1    35    1
     7    30     8    1
     8    30    35    2
     9    39    38    1
    10    35    36    1
    11    27     3    1
    12    27     2    1
    13    27    29    1
    14    18    37    1
    15     7    29    1
    16    13    34    1
    17    38    29    1
    18    38    37    1
    19    38    19    1
    20    29    26    1
    21    11    31    1
    22    37    34    1
    23    37    17    1
    24    36    16    1
    25    36    31    1
    26    36    15    1
```

```
  27   34   14   1
  28   34   33   1
  29   31   10   1
  30   31   32   1
  31   26   24   1
  32   26   23   1
  33   26   25   1
  34   33   32   2
  35   33   28   1
  36   32   12   1
  37    5   28   1
  38   28    6   1
  39   28    4   1
```

Compound 38

```
@<TRIPOS>MOLECULE
compuesto_38.out
 39 40 0 0 0
SMALL
MULLIKEN_CHARGES

@<TRIPOS>ATOM
      1 C          6.0731    0.9337   -14.4273 C.3     1  UNL1       -0.2571
      2 H          6.6441   -0.7316   -16.4711 H       1  UNL1        0.1586
      3 H          5.5319   -1.1660   -17.8354 H       1  UNL1        0.1558
      4 H          2.5413   -0.9626   -16.1132 H       1  UNL1        0.1634
      5 H          3.1736   -0.7616   -17.7583 H       1  UNL1        0.1632
      6 H          2.7441    0.6749   -16.7816 H       1  UNL1        0.1632
      7 H          2.3462   -2.2410   -12.3431 H       1  UNL1        0.1488
      8 H          2.2354   -2.3711   -10.5802 H       1  UNL1        0.1462
      9 H          3.7159   -2.8802   -11.4036 H       1  UNL1        0.1494
     10 H          5.7594   -2.1025   -11.7054 H       1  UNL1        0.1497
     11 H          6.1965   -0.6574   -10.7621 H       1  UNL1        0.1519
     12 H          6.7539   -0.8392   -12.4310 H       1  UNL1        0.1555
     13 H          4.2823    3.1581   -11.6351 H       1  UNL1        0.1403
     14 H          3.0722    1.7132    -9.7487 H       1  UNL1        0.1371
     15 H          2.0318    2.4564   -10.9889 H       1  UNL1        0.1313
     16 H          1.5017    0.0020   -10.5847 H       1  UNL1        0.1287
     17 H          1.8855    0.3842   -12.2823 H       1  UNL1        0.1378
     18 H          3.9214   -0.6504   -10.2083 H       1  UNL1        0.1241
     19 H          6.9521    1.5832   -12.5264 H       1  UNL1        0.1422
     20 H          5.8940    2.7813   -13.2905 H       1  UNL1        0.1369
     21 H          4.6917   -1.6784   -13.9283 H       1  UNL1        0.1445
     22 H          3.1565   -0.8010   -13.8061 H       1  UNL1        0.1383
     23 H          3.9777    1.2104   -14.7489 H       1  UNL1        0.1379
     24 H          6.4172    1.6190   -15.2285 H       1  UNL1        0.1316
     25 H          6.8227    0.1186   -14.3657 H       1  UNL1        0.1350
     26 C          5.6321   -0.7390   -16.8508 C.2     1  UNL1       -0.4544
     27 C          3.1992   -0.3306   -16.7350 C.3     1  UNL1       -0.5117
     28 C          4.5848   -0.2638   -16.1648 C.2     1  UNL1        0.1332
     29 C          2.9197   -2.1209   -11.4091 C.3     1  UNL1       -0.4872
     30 C          5.8959   -1.0107   -11.7637 C.3     1  UNL1       -0.5094
     31 C          4.0562    2.0892   -11.6046 C.2     1  UNL1       -0.2391
     32 C          2.8327    1.7034   -10.8447 C.3     1  UNL1       -0.2423
     33 C          2.3180    0.3243   -11.2619 C.3     1  UNL1       -0.2839
     34 C          3.4707   -0.6983   -11.2380 C.3     1  UNL1       -0.0701
     35 C          6.0004    1.6939   -13.0873 C.3     1  UNL1       -0.2870
     36 C          4.8453    1.2089   -12.2398 C.2     1  UNL1        0.0116
     37 C          4.6058   -0.2975   -12.2424 C.3     1  UNL1        0.1436
     38 C          4.2542   -0.6858   -13.7072 C.3     1  UNL1       -0.3119
     39 C          4.6925    0.3449   -14.7757 C.3     1  UNL1       -0.1058
@<TRIPOS>BOND
     1    3   26   1
     2    5   27   1
     3   26    2   1
     4   26   28   2
```

```
    5    6   27    1
    6   27   28    1
    7   27    4    1
    8   28   39    1
    9   24    1    1
   10   39   23    1
   11   39    1    1
   12   39   38    1
   13    1   25    1
   14    1   35    1
   15   21   38    1
   16   22   38    1
   17   38   37    1
   18   20   35    1
   19   35   19    1
   20   35   36    1
   21   12   30    1
   22    7   29    1
   23   17   33    1
   24   37   36    1
   25   37   30    1
   26   37   34    1
   27   36   31    2
   28   30   10    1
   29   30   11    1
   30   13   31    1
   31   31   32    1
   32   29    9    1
   33   29   34    1
   34   29    8    1
   35   33   34    1
   36   33   32    1
   37   33   16    1
   38   34   18    1
   39   15   32    1
   40   32   14    1
```

Compound 39

```
@<TRIPOS>MOLECULE
compuesto_39.out
 39 40 0 0 0
SMALL
MULLIKEN_CHARGES

@<TRIPOS>ATOM
      1 C          5.2502    1.4296  -14.9186 C.3     1  UNL1       -0.2663
      2 H          4.5737   -0.9624  -17.8576 H       1  UNL1        0.1626
      3 H          6.2851   -0.8671  -18.3277 H       1  UNL1        0.1621
      4 H          3.5218    2.9773  -11.4651 H       1  UNL1        0.1278
      5 H          2.2319    1.8377  -11.8885 H       1  UNL1        0.1374
      6 H          4.4045    1.5972   -9.7206 H       1  UNL1        0.1354
      7 H          2.6326    1.4031   -9.5563 H       1  UNL1        0.1324
      8 H          3.6879   -0.8480   -9.3967 H       1  UNL1        0.1401
      9 H          4.5569    3.0981  -13.7163 H       1  UNL1        0.1283
     10 H          3.2428    2.1144  -14.3904 H       1  UNL1        0.1338
     11 H          5.2407    1.2566  -12.2051 H       1  UNL1        0.1299
     12 H          5.8461   -0.8169  -13.3438 H       1  UNL1        0.1436
     13 H          4.6220   -1.8903  -14.0695 H       1  UNL1        0.1405
     14 H          3.9124   -0.1159  -15.6458 H       1  UNL1        0.1322
     15 H          5.2402    2.0007  -15.8715 H       1  UNL1        0.1270
     16 H          6.2807    1.5183  -14.5128 H       1  UNL1        0.1388
     17 C          4.0179   -2.4610  -11.4905 C.3     1  UNL1       -0.4864
     18 C          2.3824   -0.4428  -13.3956 C.3     1  UNL1       -0.4993
     19 C          7.0557   -1.2406  -15.8506 C.2     1  UNL1       -0.4492
     20 C          5.5313   -0.4551  -17.6353 C.3     1  UNL1       -0.5103
     21 C          5.9079   -0.6373  -16.1860 C.2     1  UNL1        0.1275
```

```
  22 C        3.2927   1.8958  -11.5702 C.3    1  UNL1      -0.2854
  23 C        3.4960   1.1878  -10.2214 C.3    1  UNL1      -0.2388
  24 C        3.6795  -0.2988  -10.3456 C.2    1  UNL1      -0.2347
  25 C        3.8550  -0.9640  -11.4997 C.2    1  UNL1       0.0227
  26 C        4.2508   2.0517  -13.9320 C.3    1  UNL1      -0.2812
  27 C        4.9292  -0.0548  -15.1792 C.3    1  UNL1      -0.1014
  28 H        1.6176  -0.1833  -12.6437 H      1  UNL1       0.1557
  29 H        3.2556  -2.9485  -12.1231 H      1  UNL1       0.1596
  30 H        5.0206  -2.7533  -11.8552 H      1  UNL1       0.1608
  31 H        3.9024  -2.8918  -10.4826 H      1  UNL1       0.1537
  32 H        7.7368  -1.6307  -16.6041 H      1  UNL1       0.1568
  33 H        2.1765   0.1652  -14.2896 H      1  UNL1       0.1507
  34 H        2.2042  -1.4968  -13.6750 H      1  UNL1       0.1479
  35 H        5.4193   0.6130  -17.8730 H      1  UNL1       0.1626
  36 H        7.3734  -1.3714  -14.8356 H      1  UNL1       0.1583
  37 C        4.2001   1.2454  -12.6248 C.3    1  UNL1      -0.0956
  38 C        3.8152  -0.2467  -12.8526 C.3    1  UNL1       0.1415
  39 C        4.8563  -0.8302  -13.8450 C.3    1  UNL1      -0.3210
@<TRIPOS>BOND
   1    3   20    1
   2   35   20    1
   3    2   20    1
   4   20   21    1
   5   32   19    1
   6   21   19    2
   7   21   27    1
   8   15    1    1
   9   19   36    1
  10   14   27    1
  11   27    1    1
  12   27   39    1
  13    1   16    1
  14    1   26    1
  15   10   26    1
  16   33   18    1
  17   13   39    1
  18   26    9    1
  19   26   37    1
  20   39   12    1
  21   39   38    1
  22   34   18    1
  23   18   38    1
  24   18   28    1
  25   38   37    1
  26   38   25    1
  27   37   11    1
  28   37   22    1
  29   29   17    1
  30    5   22    1
  31   30   17    1
  32   22    4    1
  33   22   23    1
  34   25   17    1
  35   25   24    2
  36   17   31    1
  37   24   23    1
  38   24    8    1
  39   23    6    1
  40   23    7    1
```

Compound 40

```
@<TRIPOS>MOLECULE
compuesto_40.out
 39 40 0 0 0
SMALL
MULLIKEN_CHARGES
```

```
@<TRIPOS>ATOM
     1 C          4.3022    2.0465  -14.2589 C.2     1  UNL1        0.1329
     2 C          4.7603    1.7249  -12.8450 C.3     1  UNL1       -0.1215
     3 C          4.6807    0.2122  -12.4634 C.3     1  UNL1       -0.0720
     4 C          5.0509   -0.7456  -13.6258 C.3     1  UNL1       -0.1299
     5 C          4.2716   -0.3879  -14.8956 C.3     1  UNL1       -0.2476
     6 H          2.2356    0.2989   -8.7224 H       1  UNL1        0.1597
     7 H          1.1597    1.1986   -9.8233 H       1  UNL1        0.1625
     8 H          6.2553   -4.0507  -13.5097 H      1  UNL1        0.1496
     9 H          6.3226   -2.9268  -14.8827 H      1  UNL1        0.1512
    10 H          7.1922   -2.5528  -13.3735 H      1  UNL1        0.1456
    11 H          3.7542   -4.0458  -13.3131 H      1  UNL1        0.1453
    12 H          2.8242   -2.5414  -13.4220 H      1  UNL1        0.1503
    13 H          3.7446   -3.1248  -14.8261 H      1  UNL1        0.1500
    14 C          4.6348    1.0355  -15.3293 C.3     1  UNL1       -0.3171
    15 H          1.3938   -0.5519  -10.0342 H      1  UNL1        0.1589
    16 H          5.0261   -2.3287  -12.1314 H      1  UNL1        0.1163
    17 H          3.5263    4.0147  -13.9217 H      1  UNL1        0.1588
    18 H          3.4703    3.4434  -15.6279 H      1  UNL1        0.1568
    19 H          4.6192    3.5652  -11.6505 H      1  UNL1        0.1270
    20 H          3.0425    2.8716  -12.1161 H      1  UNL1        0.1483
    21 H          4.8495    1.6669   -9.9372 H       1  UNL1        0.1445
    22 H          3.3489    2.6033   -9.7139 H       1  UNL1        0.1386
    23 H          2.8549   -0.9955  -11.9603 H      1  UNL1        0.1517
    24 H          6.1370   -0.5155  -13.8480 H      1  UNL1        0.1252
    25 H          3.1787   -0.4599  -14.7190 H      1  UNL1        0.1444
    26 H          4.5260   -1.0910  -15.7146 H      1  UNL1        0.1276
    27 H          4.1174    1.2716  -16.2826 H      1  UNL1        0.1446
    28 H          5.7222    1.1037  -15.5499 H      1  UNL1        0.1451
    29 C          3.7652   -3.0234  -13.7281 C.3     1  UNL1       -0.4900
    30 C          6.2656   -2.9810  -13.7829 C.3     1  UNL1       -0.5010
    31 C          1.9045    0.3857   -9.7696 C.3     1  UNL1       -0.4949
    32 H          5.4879    0.0595  -11.6930 H      1  UNL1        0.1216
    33 H          5.8535    1.9838  -12.8420 H      1  UNL1        0.1284
    34 C          5.0104   -2.2574  -13.2480 C.3     1  UNL1        0.0006
    35 C          3.7452    3.2121  -14.6137 C.2     1  UNL1       -0.4754
    36 C          4.0702    2.6106  -11.7715 C.3     1  UNL1       -0.2587
    37 C          3.8769    1.9202  -10.4079 C.3     1  UNL1       -0.2959
    38 C          3.0691    0.6717  -10.6652 C.2     1  UNL1        0.0759
    39 C          3.4160   -0.1003  -11.7060 C.2     1  UNL1       -0.2572
@<TRIPOS>BOND
     1    27    14    1
     2    26     5    1
     3    18    35    1
     4    28    14    1
     5    14     5    1
     6    14     1    1
     7     5    25    1
     8     5     4    1
     9     9    30    1
    10    13    29    1
    11    35     1    2
    12    35    17    1
    13     1     2    1
    14    24     4    1
    15    30     8    1
    16    30    10    1
    17    30    34    1
    18    29    12    1
    19    29    11    1
    20    29    34    1
    21     4    34    1
    22     4     3    1
    23    34    16    1
    24     2    33    1
```

```
   25    2    3   1
   26    2   36   1
   27    3   39   1
   28    3   32   1
   29   20   36   1
   30   23   39   1
   31   36   19   1
   32   36   37   1
   33   39   38   2
   34   38   37   1
   35   38   31   1
   36   37   21   1
   37   37   22   1
   38   15   31   1
   39    7   31   1
   40   31    6   1
```

Compound 41

```
@<TRIPOS>MOLECULE
compuesto_41.out
 39 40 0 0 0
SMALL
MULLIKEN_CHARGES

@<TRIPOS>ATOM
      1 H          3.7165    2.7411  -16.2888 H      1  UNL1        0.1554
      2 H          3.4312    2.2590  -14.5557 H      1  UNL1        0.1621
      3 H          5.5867   -0.0075  -17.2526 H      1  UNL1        0.1634
      4 H          5.3642    1.7061  -17.6513 H      1  UNL1        0.1627
      5 H          6.8039    1.1915  -16.7355 H      1  UNL1        0.1623
      6 C          5.4107    0.2720  -14.4371 C.3    1  UNL1       -0.1127
      7 H          2.8580   -1.6457   -9.4925 H      1  UNL1        0.1361
      8 H          5.6039   -1.2583  -10.8326 H      1  UNL1        0.1389
      9 H          4.6500   -2.7583  -10.7542 H      1  UNL1        0.1314
     10 H          5.9877    0.4892  -12.3363 H      1  UNL1        0.1356
     11 H          5.1774    1.9017  -12.9910 H      1  UNL1        0.1384
     12 H          5.2876   -1.6597  -15.4580 H      1  UNL1        0.1237
     13 H          5.8734   -1.9939  -13.0151 H      1  UNL1        0.1373
     14 H          4.5016   -2.9798  -13.5352 H      1  UNL1        0.1350
     15 H          3.7082   -1.0028  -14.9697 H      1  UNL1        0.1405
     16 H          6.5214    0.1566  -14.5203 H      1  UNL1        0.1254
     17 C          2.5089   -1.9050  -12.3212 C.3    1  UNL1       -0.5229
     18 H          3.0241    0.4531  -13.1360 H      1  UNL1        0.1430
     19 C          5.7222    1.0173  -16.8626 C.3    1  UNL1       -0.5130
     20 C          4.0007    2.1050  -15.4566 C.2    1  UNL1       -0.4529
     21 C          4.9830    1.2020  -15.5650 C.2    1  UNL1        0.1335
     22 C          3.0373    2.4049  -11.2697 C.3    1  UNL1       -0.4976
     23 C          3.4625    0.9651  -11.1204 C.2    1  UNL1        0.0663
     24 C          3.4319    0.3711   -9.9186 C.2    1  UNL1       -0.2472
     25 C          3.7914   -1.0612   -9.6822 C.3    1  UNL1       -0.2258
     26 C          4.5685   -1.6581  -10.8630 C.3    1  UNL1       -0.3208
     27 C          5.1534    0.7950  -13.0028 C.3    1  UNL1       -0.2748
     28 C          3.8458    0.2453  -12.3977 C.3    1  UNL1       -0.1433
     29 C          3.9305   -1.3070  -12.2297 C.3    1  UNL1        0.1923
     30 C          4.8128   -1.9314  -13.3490 C.3    1  UNL1       -0.3236
     31 C          4.7632   -1.1167  -14.6453 C.3    1  UNL1       -0.2540
     32 H          4.4082   -1.1538   -8.7630 H      1  UNL1        0.1282
     33 H          3.1107    0.9315   -9.0357 H      1  UNL1        0.1410
     34 H          2.6732    2.8336  -10.3284 H      1  UNL1        0.1554
     35 H          3.8795    3.0399  -11.5993 H      1  UNL1        0.1616
     36 H          2.2244    2.5026  -12.0054 H      1  UNL1        0.1596
     37 H          2.0865   -1.7873  -13.3339 H      1  UNL1        0.1503
     38 H          2.5127   -2.9829  -12.0907 H      1  UNL1        0.1491
     39 H          1.8131   -1.4173  -11.6243 H      1  UNL1        0.1599
@<TRIPOS>BOND
    1    4   19   1
```

```
2 3 19 1
3 19 5 1
4 19 21 1
5 1 20 1
6 21 20 2
7 21 6 1
8 12 31 1
9 20 2 1
10 15 31 1
11 31 6 1
12 31 30 1
13 16 6 1
14 6 27 1
15 14 30 1
16 30 13 1
17 30 29 1
18 37 17 1
19 18 28 1
20 27 11 1
21 27 28 1
22 27 10 1
23 28 29 1
24 28 23 1
25 17 29 1
26 17 38 1
27 17 39 1
28 29 26 1
29 36 22 1
30 35 22 1
31 22 23 1
32 22 34 1
33 23 24 2
34 26 8 1
35 26 9 1
36 26 25 1
37 24 25 1
38 24 33 1
39 25 7 1
40 25 32 1
```

Compound 42

```
@<TRIPOS>MOLECULE
compuesto_42.out
 39 40 0 0 0
SMALL
MULLIKEN_CHARGES

@<TRIPOS>ATOM
    1 C     4.5693  -0.4535  -12.1725 C.2   1  UNL1   -0.2521
    2 C     5.5328   1.3727  -15.5107 C.3   1  UNL1   -0.2753
    3 C     4.1321   0.1515  -11.0559 C.2   1  UNL1    0.0736
    4 C     3.0423   1.1879  -11.1834 C.3   1  UNL1   -0.2915
    5 C     3.3155   2.1199  -12.3821 C.3   1  UNL1   -0.2550
    6 C     4.6307  -0.1889   -9.6834 C.3   1  UNL1   -0.4938
    7 C     4.7586   3.5973  -14.5884 C.3   1  UNL1   -0.4808
    8 H     2.8510  -0.3545  -13.4444 H     1  UNL1    0.1339
    9 C     4.6403  -2.3322  -14.6269 C.3   1  UNL1    0.0015
   10 C     4.9468  -2.9949  -15.9859 C.3   1  UNL1   -0.4972
   11 C     3.3702  -2.9855  -14.0482 C.3   1  UNL1   -0.4965
   12 H     6.5055   1.8966  -15.6502 H     1  UNL1    0.1371
   13 H     4.9972   1.4238  -16.4866 H     1  UNL1    0.1386
   14 H     6.5287  -0.1336  -14.2824 H     1  UNL1    0.1412
   15 H     6.3122  -0.5837  -15.9962 H     1  UNL1    0.1264
   16 H     3.7792  -0.5983  -15.5820 H     1  UNL1    0.1283
   17 H     5.3456  -1.2169  -12.1435 H     1  UNL1    0.1502
   18 H     2.0755   0.6510  -11.3125 H     1  UNL1    0.1437
```

19	H	2.9457	1.7878	-10.2545	H	1	UNL1	0.1373
20	H	2.3649	2.5792	-12.7281	H	1	UNL1	0.1361
21	H	3.9819	2.9357	-12.0188	H	1	UNL1	0.1427
22	H	5.4081	-0.9730	-9.6990	H	1	UNL1	0.1590
23	H	5.0680	0.7048	-9.1988	H	1	UNL1	0.1620
24	H	3.7968	-0.5556	-9.0544	H	1	UNL1	0.1592
25	H	4.0280	4.1194	-13.9419	H	1	UNL1	0.1538
26	H	5.7678	3.9706	-14.3266	H	1	UNL1	0.1579
27	H	4.5361	3.9076	-15.6274	H	1	UNL1	0.1549
28	H	5.4930	-2.5624	-13.9431	H	1	UNL1	0.1122
29	H	5.9187	-2.6745	-16.4036	H	1	UNL1	0.1462
30	H	4.9983	-4.0978	-15.8893	H	1	UNL1	0.1478
31	H	4.1628	-2.7670	-16.7298	H	1	UNL1	0.1502
32	H	3.1727	-2.6679	-13.0067	H	1	UNL1	0.1550
33	H	2.4819	-2.7409	-14.6575	H	1	UNL1	0.1490
34	H	3.4621	-4.0890	-14.0233	H	1	UNL1	0.1461
35	C	4.7106	2.0967	-14.4734	C.2	1	UNL1	0.0069
36	C	4.0084	1.4299	-13.5391	C.2	1	UNL1	-0.0529
37	C	3.9381	-0.0856	-13.4934	C.3	1	UNL1	-0.0660
38	C	4.5059	-0.7897	-14.7496	C.3	1	UNL1	-0.1214
39	C	5.8176	-0.0891	-15.1354	C.3	1	UNL1	-0.2684

@<TRIPOS>BOND

1	31	10	1
2	13	2	1
3	29	10	1
4	15	39	1
5	10	30	1
6	10	9	1
7	12	2	1
8	27	7	1
9	16	38	1
10	2	39	1
11	2	35	1
12	39	38	1
13	39	14	1
14	38	9	1
15	38	37	1
16	33	11	1
17	9	11	1
18	9	28	1
19	7	35	1
20	7	26	1
21	7	25	1
22	35	36	2
23	11	34	1
24	11	32	1
25	36	37	1
26	36	5	1
27	37	8	1
28	37	1	1
29	20	5	1
30	5	21	1
31	5	4	1
32	1	17	1
33	1	3	2
34	18	4	1
35	4	3	1
36	4	19	1
37	3	6	1
38	22	6	1
39	6	23	1
40	6	24	1

Compound 43

@<TRIPOS>MOLECULE
compuesto_43.out

```
 40 42 0 0 0
SMALL
MULLIKEN_CHARGES

@<TRIPOS>ATOM
     1 C          3.2328    1.3531  -10.8929 C.3     1  UNL1       -0.2240
     2 C          2.6310    0.0286  -11.2973 C.3     1  UNL1       -0.2103
     3 C          3.2133   -0.6664  -12.5161 C.3     1  UNL1       -0.2072
     4 H          6.3369    1.7435  -14.2169 H       1  UNL1        0.1364
     5 H          5.3703    1.9768  -15.7015 H       1  UNL1        0.1341
     6 H          2.8445    3.3989  -11.4673 H       1  UNL1        0.1410
     7 H          1.9901    2.2136  -12.4751 H       1  UNL1        0.1532
     8 H          4.3179    1.3029  -10.6727 H       1  UNL1        0.1379
     9 H          2.7838    1.6605   -9.9376 H       1  UNL1        0.1265
    10 H          2.4901   -1.0984  -13.2277 H       1  UNL1        0.1708
    11 H          1.5386    0.0297  -11.1848 H       1  UNL1        0.1456
    12 O          3.8823   -1.5116  -15.0971 O.3     1  UNL1       -0.5900
    13 C          5.7475   -2.2332  -13.7754 C.3     1  UNL1       -0.5798
    14 H          3.3265    1.1846  -14.5939 H       1  UNL1        0.1555
    15 H          5.2375    0.0568  -12.4393 H       1  UNL1        0.1417
    16 C          4.6361   -1.4243  -10.3842 C.3     1  UNL1       -0.4852
    17 C          2.4461   -2.5403  -10.9086 C.3     1  UNL1       -0.4942
    18 C          3.3290   -1.3276  -11.1327 C.3     1  UNL1        0.1183
    19 C          4.6977    3.6225  -13.2204 C.2     1  UNL1       -0.4668
    20 C          4.9896   -1.0256  -14.3148 C.3     1  UNL1        0.3965
    21 C          5.8404   -0.1120  -15.2297 C.3     1  UNL1       -0.3876
    22 C          5.5012    1.3297  -14.8206 C.3     1  UNL1       -0.2519
    23 C          4.4260   -0.0932  -13.2026 C.3     1  UNL1       -0.1164
    24 C          4.2262    1.2646  -13.9336 C.3     1  UNL1       -0.1230
    25 C          4.0299    2.4763  -13.0467 C.2     1  UNL1        0.1223
    26 C          2.9627    2.4124  -11.9764 C.3     1  UNL1       -0.3047
    27 H          6.9134   -0.2972  -15.0828 H       1  UNL1        0.1459
    28 H          5.6231   -0.3065  -16.2972 H       1  UNL1        0.1502
    29 H          5.4438    3.8135  -13.9627 H       1  UNL1        0.1574
    30 H          4.5392    4.4907  -12.6089 H       1  UNL1        0.1553
    31 H          2.1959   -2.6378   -9.8379 H       1  UNL1        0.1560
    32 H          1.4891   -2.4709  -11.4518 H       1  UNL1        0.1551
    33 H          2.9437   -3.4536  -11.2834 H       1  UNL1        0.1581
    34 H          5.1922   -2.3175  -10.6451 H       1  UNL1        0.1518
    35 H          5.2998   -0.5644  -10.5667 H       1  UNL1        0.1560
    36 H          4.4660   -1.5187   -9.3101 H       1  UNL1        0.1559
    37 H          6.5717   -1.9151  -13.1225 H       1  UNL1        0.1665
    38 H          5.0751   -2.8655  -13.1738 H       1  UNL1        0.1825
    39 H          6.1452   -2.8521  -14.5987 H       1  UNL1        0.1598
    40 H          4.2060   -2.0239  -15.8678 H       1  UNL1        0.3108
@<TRIPOS>BOND
     1    28    21    1
     2    40    12    1
     3     5    22    1
     4    21    27    1
     5    21    22    1
     6    21    20    1
     7    12    20    1
     8    22     4    1
     9    22    24    1
    10    39    13    1
    11    14    24    1
    12    20    13    1
    13    20    23    1
    14    29    19    1
    15    24    23    1
    16    24    25    1
    17    13    38    1
    18    13    37    1
    19    10     3    1
    20    19    25    2
```

```
  21   19   30   1
  22   23    3   1
  23   23   15   1
  24   25   26   1
  25    3    2   1
  26    3   18   1
  27    7   26   1
  28   26    6   1
  29   26    1   1
  30   32   17   1
  31    2   11   1
  32    2   18   1
  33    2    1   1
  34   33   17   1
  35   18   17   1
  36   18   16   1
  37   17   31   1
  38    1    8   1
  39    1    9   1
  40   34   16   1
  41   35   16   1
  42   16   36   1
```

Compound 44

```
@<TRIPOS>MOLECULE
compuesto_44.out
 40 42 0 0 0
SMALL
MULLIKEN_CHARGES

@<TRIPOS>ATOM
      1 C          3.4238    0.3401  -12.6516 C.3     1  UNL1       -0.1481
      2 H          6.2208    0.7716  -15.7505 H       1  UNL1        0.1458
      3 H          5.0590    1.1382  -16.9501 H       1  UNL1        0.1456
      4 H          2.3909   -1.3815  -11.9023 H       1  UNL1        0.1352
      5 H          4.1591   -1.4042  -11.5713 H       1  UNL1        0.1413
      6 C          5.9724   -2.8573  -12.9885 C.3     1  UNL1       -0.5320
      7 H          6.7007   -1.7508  -15.3483 H       1  UNL1        0.1383
      8 H          2.4453    0.6249  -13.0947 H       1  UNL1        0.1372
      9 H          5.5037    0.3229  -13.4018 H       1  UNL1        0.1407
     10 C          2.6142    2.0849  -10.9048 C.3     1  UNL1       -0.5232
     11 C          4.7206    0.7703  -10.3925 C.3     1  UNL1       -0.5149
     12 C          3.2329    1.9834  -15.3137 C.2     1  UNL1       -0.4436
     13 O          5.0738   -3.1324  -15.2993 O.3     1  UNL1       -0.4897
     14 C          4.6271    2.1560  -12.5456 C.3     1  UNL1       -0.3553
     15 C          3.8089    1.3413  -11.4930 C.3     1  UNL1        0.1891
     16 C          3.5828   -2.0467  -13.5777 C.3     1  UNL1       -0.3140
     17 C          5.0077   -2.3173  -14.0155 C.3     1  UNL1        0.2800
     18 C          5.6028   -1.7916  -15.2758 C.3     1  UNL1        0.0348
     19 C          4.8769   -0.8694  -16.2361 C.3     1  UNL1       -0.2817
     20 C          5.1620    0.6096  -15.9891 C.3     1  UNL1       -0.3070
     21 C          4.2654    1.2243  -14.9441 C.2     1  UNL1        0.1173
     22 C          4.5851    0.9360  -13.5057 C.3     1  UNL1       -0.1311
     23 C          3.4004   -1.1499  -12.3458 C.3     1  UNL1       -0.2572
     24 H          3.7689   -1.0569  -16.3244 H       1  UNL1        0.1640
     25 H          5.2623   -1.1524  -17.2261 H       1  UNL1        0.1452
     26 H          2.9575   -1.6636  -14.4277 H       1  UNL1        0.1679
     27 H          3.1891   -3.0520  -13.3347 H       1  UNL1        0.1524
     28 H          4.0553    3.0126  -12.9483 H       1  UNL1        0.1526
     29 H          5.6303    2.4672  -12.2117 H       1  UNL1        0.1436
     30 H          2.9949    2.1974  -16.3523 H       1  UNL1        0.1579
     31 H          2.5605    2.4366  -14.5920 H       1  UNL1        0.1586
     32 H          4.1507    0.0796   -9.7659 H       1  UNL1        0.1535
     33 H          5.5876    0.2469  -10.8124 H       1  UNL1        0.1535
     34 H          5.1515    1.5719   -9.7339 H       1  UNL1        0.1570
     35 H          1.9492    2.5161  -11.7034 H       1  UNL1        0.1588
```

```
    36 H        1.9921    1.4078  -10.3111 H      1  UNL1       0.1539
    37 H        2.9944    2.8845  -10.2170 H      1  UNL1       0.1601
    38 H        6.1270   -2.1603  -12.1754 H      1  UNL1       0.1649
    39 H        5.5844   -3.8026  -12.5789 H      1  UNL1       0.1756
    40 H        6.9907   -3.0795  -13.3586 H      1  UNL1       0.1730
@<TRIPOS>BOND
     1    25    19    1
     2     3    20    1
     3    30    12    1
     4    24    19    1
     5    19    20    1
     6    19    18    1
     7    20     2    1
     8    20    21    1
     9     7    18    1
    10    12    21    2
    11    12    31    1
    12    13    18    1
    13    13    17    1
    14    18    17    1
    15    21    22    1
    16    26    16    1
    17    17    16    1
    18    17     6    1
    19    16    27    1
    20    16    23    1
    21    22     9    1
    22    22     1    1
    23    22    14    1
    24    40     6    1
    25     8     1    1
    26     6    39    1
    27     6    38    1
    28    28    14    1
    29     1    23    1
    30     1    15    1
    31    14    29    1
    32    14    15    1
    33    23     4    1
    34    23     5    1
    35    35    10    1
    36    15    10    1
    37    15    11    1
    38    10    36    1
    39    10    37    1
    40    33    11    1
    41    11    32    1
    42    11    34    1
```

Compound 45

```
@<TRIPOS>MOLECULE
compuesto_45.out
 42 43 0 0 0
SMALL
MULLIKEN_CHARGES

@<TRIPOS>ATOM
     1 H        6.3079    2.4454  -11.1006 H      1  UNL1       0.1494
     2 H        4.6247    2.5554  -10.5697 H      1  UNL1       0.1550
     3 H        5.3956    3.9228  -11.4146 H      1  UNL1       0.1485
     4 C        4.2480    0.3540  -14.2557 C.3    1  UNL1      -0.2469
     5 C        3.7106   -1.0780  -14.2576 C.3    1  UNL1      -0.3665
     6 C        4.5134   -2.0044  -13.3314 C.3    1  UNL1       0.4009
     7 C        4.3645   -1.5363  -11.8559 C.3    1  UNL1      -0.1538
     8 C        4.1829    0.0190  -11.7307 C.3    1  UNL1      -0.0598
     9 C        4.8379    0.7959  -12.8983 C.3    1  UNL1      -0.1311
```

```
10 C     3.2529  -2.2985  -11.0981 C.3    1  UNL1     -0.2401
11 C     2.6491  -1.4993   -9.9275 C.3    1  UNL1     -0.3021
12 C     2.0506  -0.2405  -10.5184 C.2    1  UNL1      0.0851
13 C     2.7596   0.4241  -11.4345 C.2    1  UNL1     -0.2655
14 C     4.8205   2.3469  -12.7541 C.3    1  UNL1      0.0002
15 H     4.7565   0.2984  -10.8008 H      1  UNL1      0.1246
16 C     0.6839   0.1629  -10.0783 C.3    1  UNL1     -0.4986
17 H     5.3258  -1.7806  -11.3353 H      1  UNL1      0.1267
18 O     3.9309  -3.3226  -13.4407 O.3    1  UNL1     -0.5985
19 C     5.9735  -2.1763  -13.7810 C.3    1  UNL1     -0.5815
20 C     5.6797   3.0169  -13.8485 C.3    1  UNL1     -0.4996
21 C     5.3151   2.8291  -11.3786 C.3    1  UNL1     -0.4989
22 H     5.0384   0.4573  -15.0185 H      1  UNL1      0.1233
23 H     3.4276   1.0252  -14.5751 H      1  UNL1      0.1321
24 H     3.7320  -1.4743  -15.2810 H      1  UNL1      0.1331
25 H     2.6529  -1.0969  -13.9262 H      1  UNL1      0.1672
26 H     5.9199   0.4981  -12.8839 H      1  UNL1      0.1219
27 H     2.4073  -2.5344  -11.7775 H      1  UNL1      0.1536
28 H     3.6470  -3.2738  -10.7611 H      1  UNL1      0.1346
29 H     1.8837  -2.0980   -9.3951 H      1  UNL1      0.1392
30 H     3.4260  -1.2467   -9.1834 H      1  UNL1      0.1425
31 H     2.3688   1.3138  -11.9151 H      1  UNL1      0.1492
32 H     3.7679   2.7001  -12.8862 H      1  UNL1      0.1146
33 H    -0.0636  -0.6227  -10.2359 H      1  UNL1      0.1616
34 H     0.6673   0.3868   -8.9900 H      1  UNL1      0.1614
35 H     0.3388   1.0550  -10.6311 H      1  UNL1      0.1591
36 H     4.1036  -3.6759  -14.3365 H      1  UNL1      0.3105
37 H     6.5429  -1.2477  -13.6276 H      1  UNL1      0.1706
38 H     6.4819  -2.9418  -13.1781 H      1  UNL1      0.1692
39 H     6.0367  -2.5008  -14.8387 H      1  UNL1      0.1605
40 H     5.6607   4.1152  -13.6977 H      1  UNL1      0.1501
41 H     6.7283   2.6964  -13.7930 H      1  UNL1      0.1488
42 H     5.3291   2.7782  -14.8691 H      1  UNL1      0.1492
@<TRIPOS>BOND
 1  24   5  1
 2  22   4  1
 3  42  20  1
 4  39  19  1
 5  23   4  1
 6  36  18  1
 7   5   4  1
 8   5  25  1
 9   5   6  1
10   4   9  1
11  20  41  1
12  20  40  1
13  20  14  1
14  19  37  1
15  19   6  1
16  19  38  1
17  18   6  1
18   6   7  1
19   9  26  1
20   9  14  1
21   9   8  1
22  32  14  1
23  14  21  1
24  31  13  1
25   7   8  1
26   7  17  1
27   7  10  1
28  27  10  1
29   8  13  1
30   8  15  1
31  13  12  2
32   3  21  1
```

```
33 21 1 1
34 21 2 1
35 10 28 1
36 10 11 1
37 35 16 1
38 12 16 1
39 12 11 1
40 33 16 1
41 16 34 1
42 11 29 1
43 11 30 1
```

Compound 46

```
@<TRIPOS>MOLECULE
compuesto_46.out
 42 43 0 0 0
SMALL
MULLIKEN_CHARGES

@<TRIPOS>ATOM
 1 H 4.7139 2.5515 -10.8014 H 1 UNL1 0.1560
 2 H 6.3936 2.4346 -11.4004 H 1 UNL1 0.1497
 3 H 4.2102 -3.6562 -14.6496 H 1 UNL1 0.3103
 4 C 4.3290 0.3753 -14.5261 C.3 1 UNL1 -0.2472
 5 C 4.8900 2.3576 -13.0040 C.3 1 UNL1 -0.0007
 6 H 4.8460 0.2858 -11.0732 H 1 UNL1 0.1248
 7 C 0.7770 0.1192 -10.3403 C.3 1 UNL1 -0.4987
 8 C 6.0697 -2.1517 -14.0863 C.3 1 UNL1 -0.5812
 9 C 5.7470 3.0415 -14.0936 C.3 1 UNL1 -0.5000
 10 C 5.3917 2.8258 -11.6262 C.3 1 UNL1 -0.4983
 11 H 5.4252 -1.7834 -11.6325 H 1 UNL1 0.1267
 12 O 4.0340 -3.3127 -13.7499 O.3 1 UNL1 -0.5991
 13 H 5.1176 0.4899 -15.2887 H 1 UNL1 0.1231
 14 H 3.5055 1.0447 -14.8385 H 1 UNL1 0.1322
 15 H 3.8185 -1.4454 -15.5677 H 1 UNL1 0.1331
 16 H 2.7437 -1.0877 -14.2046 H 1 UNL1 0.1675
 17 H 6.0025 0.5160 -13.1558 H 1 UNL1 0.1218
 18 H 2.5105 -2.5501 -12.0714 H 1 UNL1 0.1535
 19 H 3.7574 -3.2912 -11.0669 H 1 UNL1 0.1346
 20 H 1.9902 -2.1403 -9.6844 H 1 UNL1 0.1392
 21 H 3.5274 -1.2832 -9.4670 H 1 UNL1 0.1425
 22 H 2.4506 1.2994 -12.1713 H 1 UNL1 0.1492
 23 H 3.8353 2.7076 -13.1317 H 1 UNL1 0.1147
 24 H 0.0335 -0.6643 -10.5158 H 1 UNL1 0.1616
 25 H 0.7583 0.3210 -9.2519 H 1 UNL1 0.1612
 26 H 0.4278 1.0220 -10.8773 H 1 UNL1 0.1594
 27 H 6.6330 -1.2191 -13.9671 H 1 UNL1 0.1701
 28 H 6.5856 -2.9153 -13.4877 H 1 UNL1 0.1700
 29 H 6.1286 -2.4727 -15.1445 H 1 UNL1 0.1598
 30 H 5.4694 3.9178 -11.6501 H 1 UNL1 0.1480
 31 C 4.2714 0.0137 -12.0038 C.3 1 UNL1 -0.0600
 32 C 4.4630 -1.5405 -12.1454 C.3 1 UNL1 -0.1532
 33 C 4.6111 -1.9905 -13.6274 C.3 1 UNL1 0.4013
 34 C 3.7993 -1.0593 -14.5409 C.3 1 UNL1 -0.3665
 35 C 2.7509 -1.5317 -10.2124 C.3 1 UNL1 -0.3022
 36 C 3.3566 -2.3156 -11.3928 C.3 1 UNL1 -0.2400
 37 C 4.9192 0.8073 -13.1658 C.3 1 UNL1 -0.1306
 38 C 2.8482 0.4073 -11.7011 C.2 1 UNL1 -0.2653
 39 C 2.1444 -0.2699 -10.7893 C.2 1 UNL1 0.0852
 40 H 5.7231 4.1377 -13.9293 H 1 UNL1 0.1500
 41 H 6.7967 2.7249 -14.0435 H 1 UNL1 0.1488
 42 H 5.3962 2.8175 -15.1148 H 1 UNL1 0.1491
@<TRIPOS>BOND
 1 15 34 1
 2 13 4 1
 3 29 8 1
```

```
    4   42    9   1
    5   14    4   1
    6    3   12   1
    7   34    4   1
    8   34   16   1
    9   34   33   1
   10    4   37   1
   11    9   41   1
   12    9   40   1
   13    9    5   1
   14    8   27   1
   15    8   33   1
   16    8   28   1
   17   12   33   1
   18   33   32   1
   19   37   17   1
   20   37    5   1
   21   37   31   1
   22   23    5   1
   23    5   10   1
   24   22   38   1
   25   32   31   1
   26   32   11   1
   27   32   36   1
   28   18   36   1
   29   31   38   1
   30   31    6   1
   31   38   39   2
   32   30   10   1
   33   10    2   1
   34   10    1   1
   35   36   19   1
   36   36   35   1
   37   26    7   1
   38   39    7   1
   39   39   35   1
   40   24    7   1
   41    7   25   1
   42   35   20   1
   43   35   21   1
```

Compound 47

```
@<TRIPOS>MOLECULE
compuesto_47.out
 42 44 0 0 0
SMALL
MULLIKEN_CHARGES

@<TRIPOS>ATOM
      1 H          1.6187   -1.1750  -14.1055 H     1  UNL1        0.1511
      2 H          1.9968   -2.7523  -13.5010 H     1  UNL1        0.1533
      3 H          2.2146   -1.3627  -12.4086 H     1  UNL1        0.1602
      4 H          3.0349    3.5433  -14.5902 H     1  UNL1        0.1533
      5 H          1.8407    2.7535  -13.5718 H     1  UNL1        0.1502
      6 H          5.2688   -3.0630  -14.6192 H     1  UNL1        0.1563
      7 H          4.7569   -3.3038  -12.9006 H     1  UNL1        0.1857
      8 H          3.7249   -3.7414  -14.2238 H     1  UNL1        0.1372
      9 C          4.0607    0.0050  -11.6938 C.3   1  UNL1       -0.3569
     10 C          4.5547    1.4143  -14.2194 C.3   1  UNL1       -0.1141
     11 C          3.4712    0.6653  -15.0287 C.3   1  UNL1       -0.2984
     12 C          5.5786    0.3715  -13.6867 C.3   1  UNL1        0.0772
     13 C          4.7356   -0.6948  -12.8881 C.3   1  UNL1        0.2902
     14 C          6.2912   -0.3609  -14.8541 C.3   1  UNL1       -0.2970
     15 C          3.8246   -1.5598  -13.8612 C.3   1  UNL1        0.1658
     16 C          3.8596   -0.8148  -15.2405 C.3   1  UNL1       -0.1079
     17 C          5.2610   -0.8672  -15.8665 C.3   1  UNL1       -0.2696
```

```
18 C    3.2230  1.2339  -12.0419 C.3   1  UNL1   -0.2645
19 C    3.8999  2.1921  -13.0531 C.3   1  UNL1   -0.0458
20 O    5.6562  -1.6544 -12.3031 O.3   1  UNL1   -0.5992
21 C    6.6816  1.0394  -12.8407 C.3   1  UNL1   -0.5214
22 C    2.8576  3.2044  -13.5563 C.3   1  UNL1   -0.4970
23 C    4.4237  -2.9762 -13.9220 C.3   1  UNL1   -0.5080
24 C    2.3455  -1.7109 -13.4428 C.3   1  UNL1   -0.5222
25 H    5.4873  -1.9081 -16.1777 H     1  UNL1   0.1300
26 H    5.3061  -0.2495 -16.7785 H     1  UNL1   0.1273
27 H    3.1320  -1.2959 -15.9273 H     1  UNL1   0.1186
28 H    7.0185  0.2849  -15.3728 H     1  UNL1   0.1280
29 H    6.8731  -1.2153 -14.4249 H     1  UNL1   0.1485
30 H    3.2571  1.1444  -16.0071 H     1  UNL1   0.1267
31 H    2.5190  0.7137  -14.4684 H     1  UNL1   0.1422
32 H    5.0910  2.1490  -14.8617 H     1  UNL1   0.1227
33 H    4.8638  0.2887  -10.9991 H     1  UNL1   0.1374
34 H    3.4708  -0.7282 -11.0993 H     1  UNL1   0.1501
35 H    2.2451  0.9141  -12.4577 H     1  UNL1   0.1370
36 H    3.0218  1.7964  -11.1088 H     1  UNL1   0.1255
37 H    4.7038  2.7518  -12.5177 H     1  UNL1   0.1152
38 H    6.3352  -1.2194 -11.7715 H     1  UNL1   0.3030
39 H    7.4469  0.2892  -12.6272 H     1  UNL1   0.1493
40 H    7.1468  1.8544  -13.4112 H     1  UNL1   0.1575
41 H    6.3850  1.4965  -11.8822 H     1  UNL1   0.1556
42 H    2.8376  4.0703  -12.8722 H     1  UNL1   0.1471
@<TRIPOS>BOND
1  26  17  1
2  25  17  1
3  30  11  1
4  27  16  1
5  17  16  1
6  17  14  1
7  28  14  1
8  16  11  1
9  16  15  1
10  11  31  1
11  11  10  1
12  32  10  1
13  14  29  1
14  14  12  1
15  6  23  1
16  4  22  1
17  8  23  1
18  10  12  1
19  10  19  1
20  1  24  1
21  23  15  1
22  23  7  1
23  15  24  1
24  15  13  1
25  12  13  1
26  12  21  1
27  5  22  1
28  22  19  1
29  22  42  1
30  2  24  1
31  24  3  1
32  40  21  1
33  19  37  1
34  19  18  1
35  13  20  1
36  13  9  1
37  21  39  1
38  21  41  1
39  35  18  1
40  20  38  1
```

41	18	9	1
42	18	36	1
43	9	34	1
44	9	33	1
